# Generalizing the inverse FFT off the unit circle

**DOI:** 10.1038/s41598-019-50234-9

**Published:** 2019-10-08

**Authors:** Vladimir Sukhoy, Alexander Stoytchev

**Affiliations:** Department of Electrical and Computer Engineering, Iowa State University, Ames, IA 50011 USA

**Keywords:** Mathematics and computing, Electrical and electronic engineering

## Abstract

This paper describes the first algorithm for computing the inverse chirp z-transform (ICZT) in *O*(*n* log *n*) time. This matches the computational complexity of the chirp z-transform (CZT) algorithm that was discovered 50 years ago. Despite multiple previous attempts, an efficient ICZT algorithm remained elusive until now. Because the ICZT can be viewed as a generalization of the inverse fast Fourier transform (IFFT) off the unit circle in the complex plane, it has numerous practical applications in a wide variety of disciplines. This generalization enables exponentially growing or exponentially decaying frequency components, which cannot be done with the IFFT. The ICZT algorithm was derived using the properties of structured matrices and its numerical accuracy was evaluated using automated tests. A modification of the CZT algorithm, which improves its numerical stability for a subset of the parameter space, is also described and evaluated.

## Introduction

**Figure 1:**
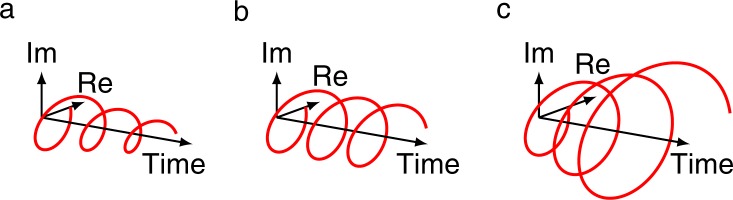
Visualization of three different types of frequency components that can be used with the CZT and the ICZT: (**a**) an exponentially decaying frequency component, (**b**) a frequency component with a fixed magnitude, and (**c**) an exponentially growing frequency component. Each point on the chirp contour determines a frequency component, where its type depends on the location of that point with respect to the unit circle. The FFT and the IFFT use only fixed-magnitude frequency components that are determined by the *n*-th roots of unity, which lie on the unit circle.

The Fourier transform and its inverse appear in many natural phenomena and have numerous applications. The fast Fourier transform (FFT) and the inverse FFT (or IFFT) algorithms compute the discrete versions of these transforms. Both of these algorithms run in $O(n\,\log \,n)$ time, which makes them practical. A generalization of the FFT off the unit circle, called the *chirp z-transform* (CZT), was published in 1969. A fast *inverse chirp z-transform* (ICZT) algorithm that generalizes the IFFT in a similar way has remained elusive for 50 years, despite multiple previous attempts. Here we describe the first ICZT algorithm that runs in $O(n\,\log \,n)$ time. It enables applications with spectral frequency components that are not constrained to have fixed magnitudes but also could decay or grow exponentially (see Fig. 1).

The CZT can use sample points from the entire complex plane and not only from the unit circle. More specifically, the transform distributes the samples along a logarithmic spiral contour (i.e., chirp contour) that is defined by the formula ${A}^{-j}\,{W}^{jk}$, where *j* denotes a zero-based input sample index and *k* denotes a zero-based output sample index. The complex numbers *A* and *W* specify the location and the direction of the spiral contour and also the spacing of the sample points along the contour.

An efficient algorithm for computing the forward chirp z-transform was described 50 years ago^1–5^. It was derived using an index substitution, which was originally proposed by Bluestein^1,5^, to compute the transform using fast convolution. It runs in $O(n\,\log \,n)$ time, where *n* is the size of the transform^4,6–8^. Various optimizations have been proposed for the CZT algorithm^9^. Its computational complexity, however, remains fixed at $O(n\,\log \,n)$, which matches the complexity of the FFT algorithm.

The ICZT is the inverse of the CZT. That is, the ICZT maps the output of the CZT back to the input. Because the CZT is a linear transform, it can be expressed using the product of the CZT transformation matrix with the input vector. This matrix can be inverted using a standard algorithm. In algorithmic form, however, this process may require up to $O({n}^{3})$ operations.

Even though there are matrix inversion algorithms^10^ that run faster than $O({n}^{3})$, at least *n*^2^ operations are necessary to compute each element of an *n*-by-*n* matrix. Thus, $O({n}^{2})$ is a lower bound for the complexity of any ICZT algorithm that works with an *n*-by-*n* matrix in memory.

Just like the FFT and the IFFT have the same computational complexity^11–14^ it is desirable to have an ICZT algorithm that matches the computational complexity of the CZT algorithm, i.e., $O(n\,\log \,n)$. This requirement rules out any method that needs to compute each element of the transformation matrix. This paper describes the first ICZT algorithm that runs in $O(n\,\log \,n)$ time. It states a working algorithm, explains how it was derived, and evaluates its numerical precision using automated test cases.

## Related Work

Several attempts to derive an efficient ICZT algorithm have been made^15–18^. In some cases^15^, a modified version of the forward CZT algorithm, in which the logarithmic spiral contour was traversed in the opposite direction, was described as the ICZT algorithm. However, this method does not really invert the CZT. It works only in some special cases, e.g., when $A=1$ and $W={e}^{-\frac{2\pi i}{n}}$. That is, in the cases when the CZT reduces to the DFT. In the general case, i.e., when $A,W\in {\mathbb{C}}\backslash \{0\}$, that method generates a transform that does not invert the CZT.

This paper describes an $O(n\,\log \,n)$ algorithm that computes the ICZT. The algorithm was derived by expressing the CZT formula using structured matrix multiplication and then finding a way to efficiently invert the matrices in the underlying matrix equation. The essence of the ICZT computation reduces to inverting a specially constructed Vandermonde matrix ***W***. This problem, in turn, reduces to inverting a symmetric Toeplitz matrix $\hat{{\boldsymbol{W}}}$ that is derived from ***W***.

The Gohberg–Semencul formula^19–21^ expresses the inverse of a Toeplitz matrix as the difference of two products of Toeplitz matrices. Each of the four matrices in this formula is either an upper-triangular or a lower-triangular Toeplitz matrix that is generated by either a vector **u** or a vector **v**. In the case of the ICZT, a symmetric Toeplitz matrix needs to be inverted. This leads to a simplified formula that expresses the inverse using only one generating vector that is also called **u**.

In the ICZT case, it turned out that each element of the generating vector **u** can be expressed as a function of the transform parameter *W*. This formula led to an efficient ICZT algorithm. One building block of this algorithm is the multiplication of a Toeplitz matrix by a vector, which can be done in $O(n\,\log \,n)$, without storing the full Toeplitz matrix in memory^22–26^. The supplementary information for this paper gives the pseudo-code for two different algorithms — based on these references — that can compute a Toeplitz–vector product in $O(n\,\log \,n)$ time. Each of these algorithms can be used as a subroutine by the ICZT algorithm.

## The CZT in Structured Matrix Notation

Structured matrices can be described with significantly fewer parameters than the number of their elements^26,27^. Some examples include: Toeplitz, Hankel, Vandermonde, Cauchy, and circulant matrices^26,28^. Diagonal matrices are structured matrices as well, i.e., an *N*-by-*N* diagonal matrix may have no more than *N* non-zero elements. Supplementary Fig. S1 illustrates the shapes of the structured matrices used in this paper and also shows their generating vectors.

The CZT is defined^4^ using the following formula:

1 $${{\rm{X}}}_{k}=\mathop{\sum }\limits_{j=0}^{N-1}\,{{\rm{x}}}_{j}\,{A}^{-j}\,{W}^{jk},\,k=0,1,\,\ldots ,\,M-1.$$

**Figure 2:**
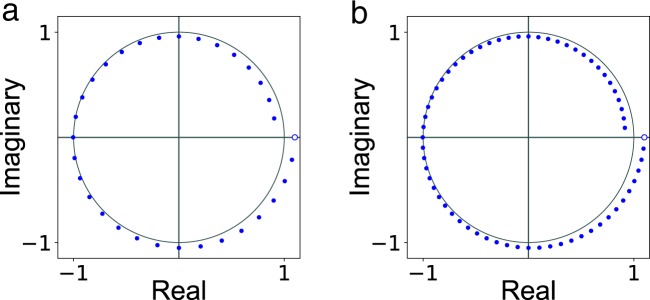
Chirp contour with $M=32$ points (**a**) and $M=64$ points (**b**). The contour is specified by $A=1.1$ and $W=\sqrt[M]{1.2}\times \exp (\frac{i2\pi }{M})$, which are the transform parameters. The unfilled circle indicates the starting point, which is equal to *A*. The end point is equal to $A\,{W}^{-(M-\mathrm{1)}}$. The blue points are given by the complex sequence ${{\rm{z}}}_{0},{{\rm{z}}}_{1},\ldots ,{{\rm{z}}}_{M-1}$, where ${{\rm{z}}}_{k}=A\,{W}^{-k}$. The *k*-th element of the CZT output vector is the z-transform at z_*k*_ of the input vector **x**, i.e., ${{\rm{X}}}_{k}={\sum }_{j=0}^{N-1}\,{{\rm{x}}}_{j}\,{{\rm{z}}}_{k}^{-j}$.

The complex numbers *A* and *W* are parameters of the transform that define the logarithmic spiral contour and the locations of the samples on it (e.g., see Fig. 2). The integer *N* specifies the size of the input vector **x**. Similarly, the integer *M* specifies the size of the output vector **X**. In general, *N* may not be equal to *M*. That is, the dimensionality of the input may not be equal to the dimensionality of the output. To analyze the complexity of the CZT algorithm it is often convenient to set $n=\,{\rm{\max }}(M,N)$.

Let ${\bf{A}}={\rm{diag}}({A}^{-0},{A}^{-1},{A}^{-2},\ldots ,{A}^{-(N-1)})$ be a diagonal matrix of size *N*-by-*N*. Then, the CZT can also be expressed with the following matrix equation:

2 $${\bf{X}}={\boldsymbol{W}}\,{\bf{A}}\,{\bf{x}}.$$

In this case, ***W*** is an *M*-by-*N* matrix that is defined as:

3 $${\boldsymbol{W}}=\mathop{\underbrace{\,[\begin{array}{ccccc}{W}^{0\cdot 0} & {W}^{1\cdot 0} & {W}^{2\cdot 0} & \ldots  & {W}^{(N-1)\cdot 0}\\ {W}^{0\cdot 1} & {W}^{1\cdot 1} & {W}^{2\cdot 1} & \ldots  & {W}^{(N-1)\cdot 1}\\ {W}^{0\cdot 2} & {W}^{1\cdot 2} & {W}^{2\cdot 2} & \ldots  & {W}^{(N-1)\cdot 2}\\ \vdots  & \vdots  & \vdots  & \ddots  & \vdots \\ {W}^{0\cdot (M-1)} & {W}^{1\cdot (M-1)} & {W}^{2\cdot (M-1)} & \ldots  & {W}^{(N-1)\cdot (M-1)}\end{array}]\,}}\limits_{{\rm{V}}{\rm{a}}{\rm{n}}{\rm{d}}{\rm{e}}{\rm{r}}{\rm{m}}{\rm{o}}{\rm{n}}{\rm{d}}{\rm{e}}\,{\rm{m}}{\rm{a}}{\rm{t}}{\rm{r}}{\rm{i}}{\rm{x}}}.$$

The matrix ***W*** is Vandermonde (i.e., each row of ***W*** forms a geometric progression). In this special case, the common ratio of each of these progressions is equal to the corresponding integer power of the parameter *W*. The negative integer powers of the transform parameter *A*, which are arranged along the diagonal of the matrix **A**, scale the columns of ***W***.

Because ***W*** is a special case of a Vandermonde matrix, it can be expressed as a product of a diagonal matrix, a Toeplitz matrix $\hat{{\boldsymbol{W}}}$, and another diagonal matrix. It is possible to express^4^ the power of the parameter *W* in each element of the matrix ***W*** using the following equation:

4 $$jk=\frac{{j}^{2}+{k}^{2}-{(k-j)}^{2}}{2}.$$

This substitution was first proposed by Bluestein^5^.

Equation (4) implies that for each $k\in \{0,1,\ldots ,M-1\}$ the right-hand side of Eq. (1) can be expressed^4^ as follows:

5 $$\begin{array}{ccc}{{\rm{X}}}_{k} & = & \mathop{\sum }\limits_{j=0}^{N-1}\,{{\rm{x}}}_{j}\,{A}^{-j}\,{W}^{jk}\\  & = & \mathop{\sum }\limits_{j=0}^{N-1}\,{{\rm{x}}}_{j}\,{A}^{-j}\,{W}^{\frac{{j}^{2}+{k}^{2}-{(k-j)}^{2}}{2}}\\  & = & \mathop{\sum }\limits_{j=0}^{N-1}\,{{\rm{x}}}_{j}\,{A}^{-j}\,{W}^{\frac{{j}^{2}}{2}}\,{W}^{\frac{{k}^{2}}{2}}\,{W}^{-\frac{{(k-j)}^{2}}{2}}.\end{array}$$

The terms of this formula can be rearranged so that it can be mapped to matrix products more easily, i.e.,

6 $${{\rm{X}}}_{k}={W}^{\frac{{k}^{2}}{2}}\,(\mathop{\sum }\limits_{j=0}^{N-1}\,{W}^{-\frac{{(k-j)}^{2}}{2}}\,({W}^{\frac{{j}^{2}}{2}}\,({A}^{-j}\,{{\rm{x}}}_{j}))).$$

In Eq. (6), the term ${W}^{\frac{{k}^{2}}{2}}$ maps to an *M*-by-*M* diagonal matrix **P**. Similarly, the term ${W}^{\frac{{j}^{2}}{2}}$ maps to a diagonal matrix **Q** that has *N* rows and *N* columns. That is,

7 $${\bf{P}}={\rm{d}}{\rm{i}}{\rm{a}}{\rm{g}}({W}^{\frac{{0}^{2}}{2}},{W}^{\frac{{1}^{2}}{2}},\ldots ,{W}^{\frac{{(M-1)}^{2}}{2}})\,{\rm{a}}{\rm{n}}{\rm{d}}\,{\bf{Q}}={\rm{d}}{\rm{i}}{\rm{a}}{\rm{g}}({W}^{\frac{{0}^{2}}{2}},{W}^{\frac{{1}^{2}}{2}},\ldots ,{W}^{\frac{{(N-1)}^{2}}{2}}).$$

The term *A*^−*j*^ maps to the following *N*-by-*N* diagonal matrix:

8 $${\bf{A}}={\rm{diag}}({A}^{-0},{A}^{-1},\ldots ,{A}^{-(N-\mathrm{1)}}).$$

Finally, ${W}^{-\frac{{(k-j)}^{2}}{2}}$ maps to an *M*-by-*N* Toeplitz matrix $\hat{{\boldsymbol{W}}}$:

9 $$\hat{{\boldsymbol{W}}}{\boldsymbol{=}}\mathop{\underbrace{\,[\begin{array}{cccc}{W}^{-\frac{{(0-0)}^{2}}{2}} & {W}^{-\frac{{(0-1)}^{2}}{2}} & \ldots  & {W}^{-\frac{{(0-(N-1))}^{2}}{2}}\\ {W}^{-\frac{{(1-0)}^{2}}{2}} & {W}^{-\frac{{(1-1)}^{2}}{2}} & \ldots  & {W}^{-\frac{{(1-(N-1))}^{2}}{2}}\\ \vdots  & \vdots  & \ddots  & \vdots \\ {W}^{-\frac{{((M-1)-0)}^{2}}{2}} & {W}^{-\frac{{((M-1)-1)}^{2}}{2}} & \ldots  & {W}^{-\frac{{((M-1)-(N-1))}^{2}}{2}}\end{array}]\,}}\limits_{{\rm{T}}{\rm{o}}{\rm{e}}{\rm{p}}{\rm{l}}{\rm{i}}{\rm{t}}{\rm{z}}\,{\rm{m}}{\rm{a}}{\rm{t}}{\rm{r}}{\rm{i}}{\rm{x}}}.$$

Since ${\boldsymbol{W}}={\bf{P}}\,\hat{{\boldsymbol{W}}}\,{\bf{Q}}$, the CZT algorithm can be viewed as an efficient implementation of the following matrix equation:

10 $${\bf{X}}={\bf{P}}\,\hat{{\boldsymbol{W}}}\,{\bf{Q}}\,{\bf{A}}\,{\bf{x}}.$$

As mentioned above, **x** is the input vector to the CZT and **X** is the output vector of the CZT. Supplementary Appendix A gives an example.

Because **P**, **Q**, and **A** are diagonal matrices, any product between any of them and a vector can be computed in $O(n)$ time. Only the matrix $\hat{{\boldsymbol{W}}}$ is a Toeplitz matrix, i.e., each of its diagonals contains the same value. As described in the literature^26,29^, the product of a Toeplitz matrix with a vector can be computed in $O(n\,\log \,n)$ time (see Supplementary Appendices B, C and D). Thus, the output vector **X** can be computed in $O(n\,\log \,n)$ time if the multiplications are performed from right to left, i.e.,

11 $${\bf{X}}={\bf{P}}(\hat{{\boldsymbol{W}}}({\bf{Q}}({\bf{A}}\,{\bf{x}}\mathrm{))).}$$

Algorithm 1 gives the pseudo-code for the CZT algorithm, which computes Eq. (11) in $O(n\,\log \,n)$ time using structured matrix multiplication. To multiply the Toeplitz matrix $\hat{{\boldsymbol{W}}}$ by a vector, the algorithm uses the circulant embedding function ToeplitzMultiplyE that is described in Supplementary Appendix B. An alternative implementation could replace line 14 with a call to ToeplitzMultiplyP, which is described in Supplementary Appendix C.

**Algorithm 1:**
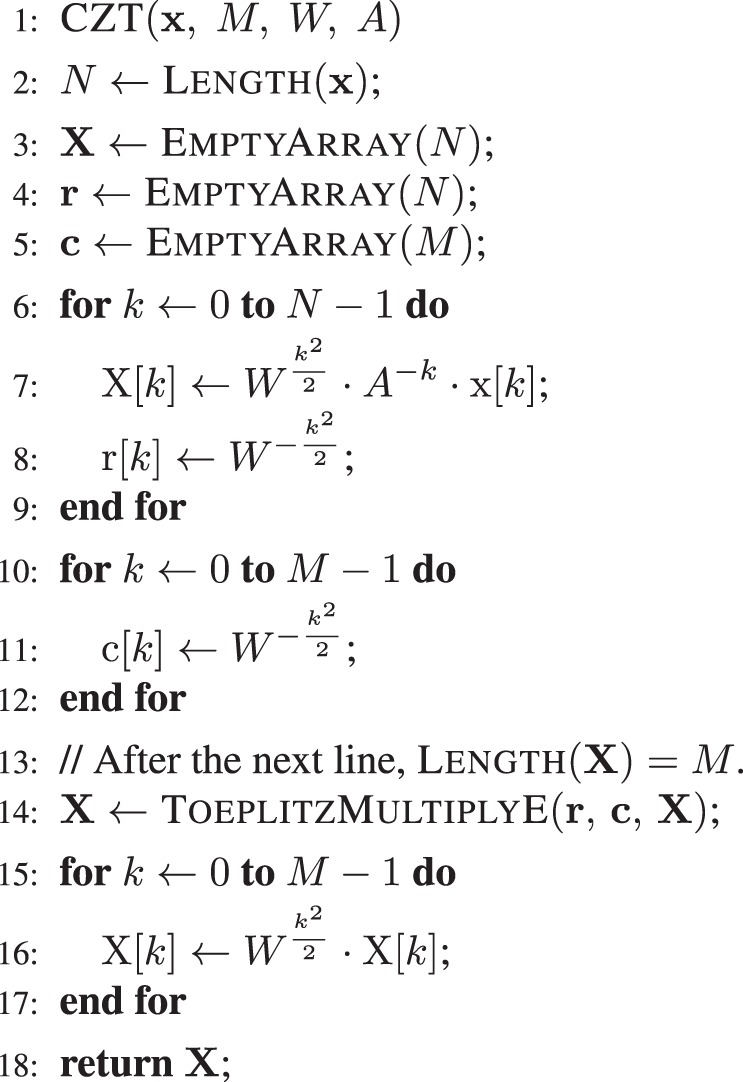
CZT Algorithm. Runs in $O(n\,\log \,n)$ time.

## The ICZT in Structured Matrix Notation

A formula for the inverse chirp z-transform exists only when *M* = *N* and can be derived by inverting the matrices in Eq. (10), i.e.,

12 $${\bf{x}}={{\bf{A}}}^{-1}\,{{\bf{Q}}}^{-1}\,{\hat{{\boldsymbol{W}}}}^{-1}\,{{\bf{P}}}^{-1}\,{\bf{X}}\mathrm{.}$$

Each matrix in Eq. (12) is diagonal, except for ${\hat{{\boldsymbol{W}}}}^{-1}$. Thus, deriving an efficient ICZT algorithm reduces to finding an efficient method for inverting the symmetric Toeplitz matrix $\hat{{\boldsymbol{W}}}$. The method used here is illustrated with the following example.

Let **T** be a non-singular 3-by-3 Toeplitz matrix generated by five complex numbers *a*, *b*, *c*, *d*, and *e*. Let $\hat{{\boldsymbol{W}}}$ be a symmetric 3-by-3 Toeplitz matrix, generated by *a*, *b*, and *c*. That is,

13 $${\bf{T}}=[\begin{array}{ccc}a & b & c\\ d & a & b\\ e & d & a\end{array}],\,\hat{{\boldsymbol{W}}}=[\begin{array}{ccc}a & b & c\\ b & a & b\\ c & b & a\end{array}].$$

The Gohberg–Semencul formula^19,20^ states that the inverse matrix **T**^−1^ can be expressed using the following equation:

14 $${{\rm{u}}}_{0}\,{{\bf{T}}}^{-1}=\mathop{\underbrace{\,[\begin{array}{ccc}{{\rm{u}}}_{0} & 0 & 0\\ {{\rm{u}}}_{1} & {{\rm{u}}}_{0} & 0\\ {{\rm{u}}}_{2} & {{\rm{u}}}_{1} & {{\rm{u}}}_{0}\end{array}]\,}}\limits_{\pmb{\mathscr{A}}}\,\mathop{\underbrace{\,[\begin{array}{ccc}{{\rm{v}}}_{2} & {{\rm{v}}}_{1} & {{\rm{v}}}_{0}\\ 0 & {{\rm{v}}}_{2} & {{\rm{v}}}_{1}\\ 0 & 0 & {{\rm{v}}}_{2}\end{array}]\,}}\limits_{\pmb{\mathscr{C}}}-\mathop{\underbrace{\,[\begin{array}{ccc}0 & 0 & 0\\ {{\rm{v}}}_{0} & 0 & 0\\ {{\rm{v}}}_{1} & {{\rm{v}}}_{0} & 0\end{array}]\,}}\limits_{\pmb{\mathscr{B}}}\,\mathop{\underbrace{\,[\begin{array}{ccc}0 & {{\rm{u}}}_{2} & {{\rm{u}}}_{1}\\ 0 & 0 & {{\rm{u}}}_{2}\\ 0 & 0 & 0\end{array}]\,}}\limits_{\pmb{\mathscr{D}}},$$

where ${\bf{u}}=({{\rm{u}}}_{0},{{\rm{u}}}_{1},{{\rm{u}}}_{2})$ is a three-element vector such that ${{\rm{u}}}_{0}\ne 0$ and ${\bf{v}}=({{\rm{v}}}_{0},{{\rm{v}}}_{1},{{\rm{v}}}_{2})$ is another three-element vector. These two vectors are determined by the numbers *a*, *b*, *c*, *d*, and *e* that generate the matrix **T**. However, expressing the elements of **u** and **v** explicitly as functions of these five numbers can be difficult. Also, **u** and **v** may not be unique.

In other words, Eq. (14) states the inverse of a 3-by-3 Toeplitz matrix **T** using four structured matrices: (1) a lower-triangular Toeplitz matrix $\pmb{\mathscr{A}}$ generated by the vector **u**, (2) an upper-triangular Toeplitz matrix $\pmb{\mathscr{C}}$ generated by the reverse of the vector **v**, (3) a lower-triangular Toeplitz matrix $\pmb{\mathscr{B}}$ generated by the vector $\mathrm{(0},{{\rm{v}}}_{0},{{\rm{v}}}_{1})$, which is obtained by shifting **v** to the right by one element, and (4) an upper-triangular Toeplitz matrix $\pmb{\mathscr{D}}$ generated by the vector $\mathrm{(0},{{\rm{u}}}_{2},{{\rm{u}}}_{1})$, which is obtained by shifting the reverse of **u** to the right by one element.

Supplementary Appendix E proves that the inverse of the symmetric Toeplitz matrix $\hat{{\boldsymbol{W}}}$ can be expressed as follows:

15 $${{\rm{u}}}_{0}\,{\hat{{\boldsymbol{W}}}}^{-1}=\mathop{\underbrace{\,[\begin{array}{ccc}{{\rm{u}}}_{0} & 0 & 0\\ {{\rm{u}}}_{1} & {{\rm{u}}}_{0} & 0\\ {{\rm{u}}}_{2} & {{\rm{u}}}_{1} & {{\rm{u}}}_{0}\end{array}]\,}}\limits_{\pmb{\mathscr{A}}}\,\mathop{\underbrace{\,[\begin{array}{ccc}{{\rm{u}}}_{0} & {{\rm{u}}}_{1} & {{\rm{u}}}_{2}\\ 0 & {{\rm{u}}}_{0} & {{\rm{u}}}_{1}\\ 0 & 0 & {{\rm{u}}}_{0}\end{array}]\,}}\limits_{{\pmb{\mathscr{A}}}^{T}}-\mathop{\underbrace{\,[\begin{array}{ccc}0 & 0 & 0\\ {{\rm{u}}}_{2} & 0 & 0\\ {{\rm{u}}}_{1} & {{\rm{u}}}_{2} & 0\end{array}]\,}}\limits_{{\pmb{\mathscr{D}}}^{T}}\,\mathop{\underbrace{\,[\begin{array}{ccc}0 & {{\rm{u}}}_{2} & {{\rm{u}}}_{1}\\ 0 & 0 & {{\rm{u}}}_{2}\\ 0 & 0 & 0\end{array}]\,}}\limits_{\pmb{\mathscr{D}}}.$$

In this case, the first two matrices are transposes of each other and so are the last two matrices. Furthermore, all four matrices are determined by one generating vector, also called **u**, which is unique. The vector **v** is not needed because it is equal to the reverse of **u** (see Supplementary Appendix E).

In general, if $\hat{{\boldsymbol{W}}}$ is a symmetric *n*-by-*n* Toeplitz matrix, then its inverse is given by the following formula:

16 $${\hat{{\boldsymbol{W}}}}^{-1}=\frac{1}{{{\rm{u}}}_{0}}\,(\pmb{\mathscr{A}}\,{\pmb{\mathscr{A}}}^{T}-{\pmb{\mathscr{D}}}^{T}\,\pmb{\mathscr{D}}),$$

where

17 $$\pmb{\mathscr{A}}\,=\,[\begin{array}{cccccc}{{\rm{u}}}_{0} & 0 & 0 & \ldots  & 0 & 0\\ {{\rm{u}}}_{1} & {{\rm{u}}}_{0} & 0 & \ldots  & 0 & 0\\ {{\rm{u}}}_{2} & {{\rm{u}}}_{1} & {{\rm{u}}}_{0} & \ldots  & 0 & 0\\ \vdots  & \vdots  & \vdots  & \ddots  & \vdots  & \vdots \\ {{\rm{u}}}_{n-2} & {{\rm{u}}}_{n-3} & {{\rm{u}}}_{n-4} & \ldots  & {{\rm{u}}}_{0} & 0\\ {{\rm{u}}}_{n-1} & {{\rm{u}}}_{n-2} & {{\rm{u}}}_{n-3} & \ldots  & {{\rm{u}}}_{1} & {{\rm{u}}}_{0}\end{array}]\,,\,\pmb{\mathscr{D}}\,=\,[\begin{array}{cccccc}0 & {{\rm{u}}}_{n-1} & {{\rm{u}}}_{n-2} & \ldots  & {{\rm{u}}}_{2} & {{\rm{u}}}_{1}\\ 0 & 0 & {{\rm{u}}}_{n-1} & \ldots  & {{\rm{u}}}_{3} & {{\rm{u}}}_{2}\\ 0 & 0 & 0 & \ldots  & {{\rm{u}}}_{4} & {{\rm{u}}}_{3}\\ \vdots  & \vdots  & \vdots  & \ddots  & \vdots  & \vdots \\ 0 & 0 & 0 & \ldots  & 0 & {{\rm{u}}}_{n-1}\\ 0 & 0 & 0 & \ldots  & 0 & 0\end{array}].$$

If the generating vector **u** is known, then the product of the matrix ${\hat{{\boldsymbol{W}}}}^{-1}$ with a vector can be computed in $O(n\,\log \,n)$ time by implementing Eq. (16) using structured matrix multiplication. For example, this can be achieved by applying four times the algorithm described in Supplementary Appendix B or the algorithm described in Supplementary Appendix C.

**Algorithm 2:**
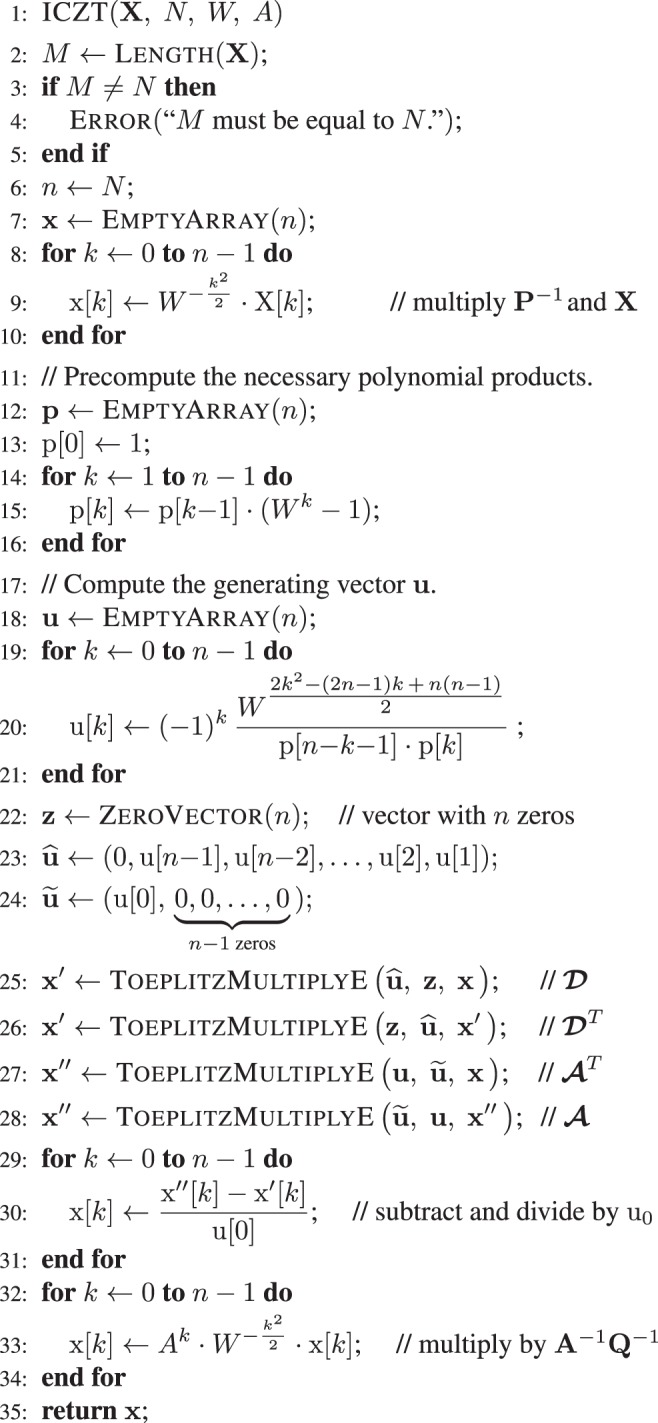
ICZT algorithm. Runs in $O(n\,\log \,n)$ time.

As proven in Supplementary Appendices E and F, the generating vector ${\bf{u}}=({{\rm{u}}}_{0},{{\rm{u}}}_{1},\ldots ,{{\rm{u}}}_{n-1}\,)$ is equal to the first column of ${\hat{{\boldsymbol{W}}}}^{-1}$ and its elements can be computed as follows:

18 $${{\rm{u}}}_{k}={({\hat{{\boldsymbol{W}}}}^{-1})}_{k+1,1}={(-1)}^{k}\frac{\displaystyle {W}^{\frac{{\scriptstyle 2{k}^{2}-(2n-1)k+n(n-1)}}{{\scriptstyle 2}}}}{\displaystyle \mathop{\prod }\limits_{s=1}^{n-k-1}\,({W}^{s}-1)\,\mathop{\prod }\limits_{s=1}^{k}\,({W}^{s}-1)}.$$

The properties of this formula are further analyzed in Supplementary Appendix F using Lagrange polynomials^30,31^.

Combining Eqs (12) and (16) leads to the following matrix equation for the ICZT:

19 $${\bf{x}}=\frac{1}{{{\rm{u}}}_{0}}{{\bf{A}}}^{-1}\,{{\bf{Q}}}^{-1}\,(\pmb{\mathscr{A}}\,{\pmb{\mathscr{A}}}^{T}-{\pmb{\mathscr{D}}}^{T}\,\pmb{\mathscr{D}})\,{{\bf{P}}}^{-1}\,{\bf{X}}.$$

Each matrix in this equation is either a diagonal matrix or a triangular Toeplitz matrix. Thus, the result vector **x** can be computed efficiently using structured matrix multiplication.

Algorithm 2 implements Eq. (19) without storing any matrices in the computer’s memory. It requires $O(n)$ memory and runs in $O(n\,\log \,n)$ time, where $n={2}^{\lceil {\log }_{2}(M+N-1)\rceil }$. There is an alternative version of the algorithm that uses ToeplitzMultiplyP instead of ToeplitzMultiplyE on lines 25–28. It also runs in $O(n\,\log \,n)$, but in that case $n={2}^{\lceil {\log }_{2}max(M,N)\rceil }$. Both algorithms assume that $M=N$.

Algorithm 2 could be optimized by reusing some partial results at the expense of making the code less modular. These optimizations, however, would not affect the overall computational complexity of the algorithm. There is one optimization, however, that is worth mentioning in more detail. The numerical accuracy of both the CZT and the ICZT can be improved if the direction of the chirp contour is reversed when the original contour is a growing logarithmic spiral, i.e., when $|W| < 1$. Contour reversal can be achieved by swapping the start point with the end point. That is, the new contour parameters are given by $W^{\prime} ={W}^{-1}$ and $A^{\prime} =A\,{W}^{-(M-1)}$. Supplementary Appendix G gives more details and proofs. It also describes the CZT-R and ICZT-R algorithms that perform this reversal and are used in some of the experiments.

## Results

Table 1 shows the results of the first experiment in which the chirp contour had the same shape but the number of points on the contour was doubled in each iteration. The numerical accuracy was computed using the CZT–ICZT procedure described in the Methods section. For all rows, the value of the transform parameter *A* was set to 1.1. The value of *W* was set to $\sqrt[M]{1.2}\times \exp (\frac{i2\pi }{M})$. Thus, for all *M*, the points were on the same chirp contour, i.e., a 360° segment of a logarithmic spiral. This was inspired by the way the FFT adds new points when the transform size is doubled (in the FFT case, however, the points are always on the unit circle). Figure 2 shows the chirp contours for $M=32$ and $M=64$.

**Table 1:** Absolute numerical error for one chirp contour with *M* points.

*M*	Condition number *κ*_2_	Size of the Floating-Point Numbers			
		64 bits	128 bits	256 bits	512 bits
32	6.1 × 10^1^	2.9 × 10^−15^	1.7 × 10^−33^	8.0 × 10^−71^	1.1 × 10^−146^
64	8.7 × 10^3^	2.2 × 10^−14^	1.4 × 10^−32^	6.5 × 10^−70^	9.0 × 10^−146^
128	2.4 × 10^8^	3.6 × 10^−12^	2.3 × 10^−30^	9.8 × 10^−68^	1.2 × 10^−143^
256	2.8 × 10^17^	1.8 × 10^−7^	1.1 × 10^−25^	5.7 × 10^−63^	8.1 × 10^−139^
512	1.7 × 10^29^	1.6 × 10^3^	1.3 × 10^−15^	4.7 × 10^−53^	6.7 × 10^−129^
1024	6.8 × 10^53^	1.9 × 10^23^	1.9 × 10^5^	6.2 × 10^−33^	8.8 × 10^−109^
2048	3.4 × 10^110^	7.1 × 10^63^	6.3 × 10^45^	3.3 × 10^8^	3.5 × 10^−68^

For all rows, the chirp contour has the same shape as in Fig. 2, but the number of points varies from *M* = 32 to *M* = 2048. Each row was computed using the CZT–ICZT procedure and averaging the results for 100 randomly selected unit-length input vectors.

Because the matrix **W** is Vandermonde, it is recommended to use double precision or higher^32^ for numerical computations. Therefore, the last four columns of Table 1 show the average error for four different IEEE-754 floating-point precisions^33^. Because some of these high-precision formats are not yet supported by modern CPUs, all floating-point formats were emulated in software using the *mpmath* library^34^.

For small values of *M*, the average numerical error is close to the machine epsilon for the corresponding floating-point precision. For large values of *M*, the numerical errors accumulate and the solutions become less accurate. This can be mitigated by increasing the floating-point precision. With 512 bits the computed vectors were accurate for all values of *M* shown in Table 1. In particular, for $M=2048$ the numerical error was on the order of 10^−68^. In other words, this problem is solvable even for large values of *M*.

The second column in Table 1 shows an estimate for the condition number ${\kappa }_{2}$, which can be viewed as an upper-bound for the sensitivity of the inverse chirp z-transform to perturbations of the input vector^22^. Its value depends on the transform parameters but not on the input vector. The results show that the average error is significantly lower than what can be expected from the condition number. This is consistent with previous observations^35^ that some ill-conditioned Vandermonde systems can be solved with small numerical error.

**Figure 3:**
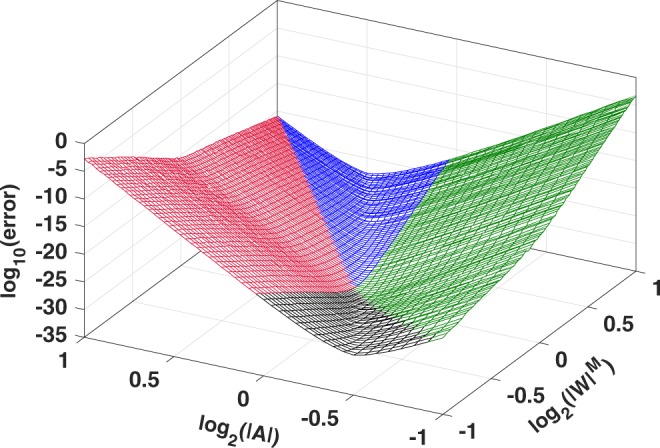
Absolute numerical error for 5,200 chirp contours. The logarithm of the error is shown as a function of $|W{|}^{M}$ and $|A|$ for $M=64$, computed with software emulation of 128-bit floating-point numbers. The lowest point of this surface corresponds to the circular contour used by the FFT and the IFFT.

Figure 3 shows the results from the second experiment in which the magnitudes of *A* and *W*^*M*^ were uniformly sampled in the range $[0.5,2.0]$. That is, 52 evenly-distributed samples for |*A*| and 100 evenly-distributed samples for $|W{|}^{M}$ were selected in that range. This resulted in 5,200 different chirp contours for which the absolute error was computed using the CZT–ICZT procedure. The logarithm of the error was averaged for 10 random input vectors and the results were plotted as a surface. The same 10 unit-length input vectors were used to compute all points on the surface. All results were computed for $M=64$ using software emulation^34^ of 128-bit floating-point numbers in IEEE-754 format^33^.

The results show that the CZT–ICZT procedure returned a vector $\hat{{\bf{x}}}$ that was very close to the original input vector **x** for all 5,200 contours. In other words, when the logarithm of the error is negative, the magnitude of the error is smaller than the magnitude of the input vector (which was of unit length).

**Figure 4:**
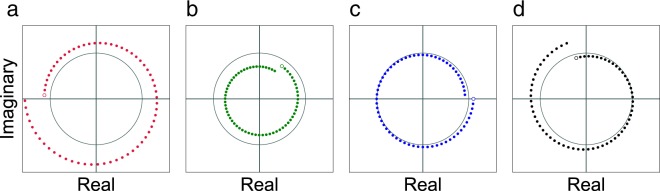
Four 64-point chirp contours, drawn in the complex plane. They show the four contour types defined based on the start point and the end point relative to the unit circle: Out–Out ((**a**) red), In–In ((**b**) green), Out–In ((**c**) blue), and In–Out ((**d**) black). The start point of each contour is indicated with an unfilled circle. The unit circle is drawn in gray.

The points in Fig. 3 are plotted with four colors that correspond to four subsets of the parameter space, which are defined by the start and end point of the chirp contour relative to the unit circle. More specifically, red is used for contours that lie entirely outside the unit circle. Green corresponds to contours that start and end within the unit circle. Blue contours start outside the unit circle but end inside it. Finally, black contours start inside the unit circle but end outside it. Figure 4 shows one example for each of these four contour types.

The polar angle of *A* does not affect the error in this experiment (see Supplementary Appendix H). Thus, to simplify the evaluation, all 5,200 contours in Fig. 3 started on the positive real axis. That is, the polar angle of *A* was set to 0 (e.g., see the blue contour in Fig. 4c).

**Figure 5:**
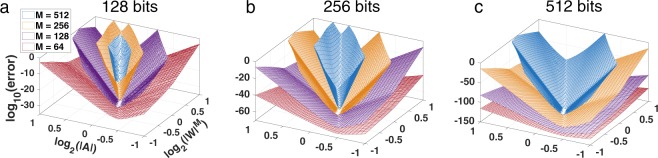
Absolute numerical error of the CZT–ICZT procedure for four values of *M* and three floating-point precisions. The error is shown as a function of $|W{|}^{M}$ and $|A|$ for $M=64,128,256$, and 512, computed using software emulation of IEEE-754 floating-point numbers with: 128 bits (**a**); 256 bits (**b**); and 512 bits (**c**). Increasing the number of bits shifts each surface down. Thus, additional bits reduce the error and increase the subset of the parameter space for which the transforms are numerically accurate (i.e., the vertical coordinate is less than zero). Each surface was computed using 5,200 chirp contours, but some points are not shown because the vertical axis was clipped at zero.

Figure 5 summarizes the results of the third experiment, which extends the second experiment by also varying the number of contour points and the number of bits used to compute the transforms. The three sub-figures were computed with 128, 256, and 512 bits, respectively. The ordering of the surfaces with respect to the vertical axis shows that the numerical error increases as *M* increases. The range of the parameter values for which the absolute numerical error is below 1 (i.e., its logarithm is negative) also shrinks as *M* increases. Conversely, increasing the number of bits lowers the surfaces and increases the size of the parameter region where the error is small. This shows that the problem can be solved for any *M*, given the right number of bits.

Supplementary Appendix I provides additional analysis and gives an error estimation formula. This formula expresses the numerical accuracy in terms of the transform parameters and the number of bits used to compute the transforms. This information can be used to select the number of bits that are sufficient to achieve the desired numerical accuracy.

Finally, it is worth emphasizing that the large scope of this evaluation was made possible by the $O(n\,\log \,n)$ computational complexity of the ICZT algorithm, which is the main contribution of this paper.

## Discussion

The discrete Fourier transform (DFT) and its efficient implementation using the fast Fourier transform (FFT) are used in a large number of applications^36–40^. Because the CZT is a generalization of the DFT and the ICZT is a generalization of the inverse DFT, the number of potential applications of the ICZT algorithm is also very large. So far, only the CZT algorithm had the same computational complexity as the FFT, i.e., $O(n\,\log \,n)$. This paper described the first ICZT algorithm that runs in $O(n\,\log \,n)$ time, which matches the computational complexity of the CZT algorithm and also of the inverse FFT.

In other words, this paper is transformative not only because it implements a transform that generalizes the inverse FFT, but also because the new algorithm has the same run-time complexity as the algorithm that it generalizes. Furthermore, this generalization enables the use of exponentially growing or decaying frequency components (see Fig. 1).

The evaluations in this paper were performed for chirp contours that are logarithmic spirals that span a 360° arc. This was done to preserve the analogy to the FFT and the IFFT. Both the CZT and the ICZT, however, can be computed for chirp contours that span smaller angular arcs or chirp contours with multiple revolutions on or off the unit circle. Future work could analyze the stability and the error properties of the algorithms in those special cases. Future work could also pursue hardware implementations of the ICZT algorithm.

## Methods

The numerical accuracy of the ICZT algorithm was evaluated with three experiments. In the first experiment, the chirp contour was held fixed while the number of points on it was doubled in each iteration. In the second experiment, the number of points was held fixed while the contour parameters were sampled uniformly on a grid. The third experiment varied both the number of points and the contour parameters. All three experiments used the procedure described below.

### CZT–ICZT procedure

The main operation in all experiments consisted of the following five steps: 1) generate each element of a random input vector **x** using uniform sampling in the range $[\,-\,1,1)$; 2) normalize the vector **x** to have unit length; 3) use the CZT algorithm to compute the vector $\hat{{\bf{X}}}$ from the vector **x**; 4) use the ICZT algorithm to compute the vector $\hat{{\bf{x}}}$ from the vector $\hat{{\bf{X}}}$; and 5) compute the absolute numerical error as the Euclidean distance between the vectors ${\bf{x}}$ and $\hat{{\bf{x}}}$. This sequence of steps is repeated several times and the results are averaged to compute the mean error. In all three experiments the transforms were computed for the square case in which $M=N$ for invertibility reasons.

The length of the vector **x** is determined by the transform parameter *M*. In the experiments, *M* was always a power of 2, but this is not a restriction of the algorithms, which can run for any *M*. In other words, the dependency algorithms, which are described in the supplementary information, check the sizes of their input vectors and pad them with zeros when necessary.

### First experiment

The value of *M* was varied from 32 to 2048 such that it was always a power of 2. Each number reported in Table 1 was averaged over 100 random input vectors **x**. These vectors were held fixed for each row of Table 1, which was achieved by using a fixed random seed to initialize the pseudo-random number generator. Each row corresponds to a different value of *M*, which, in the square case, determines the matrix size and also the lengths of the vectors **x**, $\hat{{\bf{X}}}$, and $\hat{{\bf{x}}}$. The input vectors were different for different rows because they had different lengths, i.e., different *M*. The CZT and the ICZT were computed using Algorithms 1 and 2, respectively.

The second column of Table 1 reports the condition number^22^ for the transform matrix ***W*** **A**. It depends on the transform parameters but not on the input vector. The condition number is the same for both the CZT and the ICZT.

### Condition number

The condition number ${\kappa }_{2}$ is equal to the product of the norms of the CZT matrix ***W*** **A** and the ICZT matrix ${({\boldsymbol{W}}{\bf{A}})}^{-1}$. That is, ${\kappa }_{2}=\parallel {\boldsymbol{W}}\,{\bf{A}}\parallel \cdot \parallel {({\boldsymbol{W}}{\bf{A}})}^{-1}\parallel$. This is equivalent to ${\kappa }_{2}={\sigma }_{max}\,/\,{\sigma }_{min}$, where *σ*_max_ is the maximum singular value and *σ*_min_ is the minimum singular value of the matrix ***W*** **A**. The estimates for the values of ${\kappa }_{2}$ were computed using standard double-precision floating-point numbers in IEEE-754 format with the *numpy* library.

### Floating-point precisions

Because the transform matrix can have very high condition numbers, the remaining columns of Table 1 report the average numerical error for four different floating-point precisions, i.e., for 64, 128, 256, and 512 bits. In all cases, the number of precision bits, *p*, was derived according to the IEEE-754 (2008) standard^33^.

That is, for the four storage widths used here the value of *p* was set to 53, 113, 237, and 489, respectively. Because some of these high-precision formats are not yet implemented by modern processors or standard compilers, all floating-point operations were emulated in software using the *mpmath* library^34^. This library implements complex exponentiation in a way that slightly boosts its numerical precision. For example, for 64-bit floating-point numbers the numerical error could be one or two orders of magnitude lower than what can be obtained by using a hardware implementation. This slight shift does not affect the overall behavior of the numerical error as the value of *M* increases.

The code for all experiments was implemented in Python, version 2.7. The *numpy* library was used to generate random numbers for the input vectors. In all cases, 64-bit floating point random numbers were generated and promoted to higher floating-point precisions if necessary.

### Second experiment

In this case, *M* was held fixed at 64, but the transform parameters *A* and *W* were varied. More specifically, 52 values for *A* were uniformly sampled in the interval $[0.5,2]$. Similarly, 100 values of |*W*|^*M*^ were uniformly sampled from the interval $[0.5,2]$. Both 0.5 and 2 were included among the values for $|A|$ and $|W{|}^{M}$. This resulted in 5,200 chirp contours, each specified by an $(A,W)$ pair.

For each $(A,W)$ pair, Fig. 3 shows the average absolute numerical error of the CZT–ICZT procedure computed using 10 random vectors. These 10 vectors were the same for all points of the surface. The results were computed using 128-bit floating-point numbers. As proven in Supplementary Appendix H, the polar angle of *A* does not affect the magnitude of the numerical error in this experiment. Therefore, the experimental design was simplified by setting the polar angle of *A* to zero for all contours. Only the magnitude of *A* was varied. In other words, the starting point of each of the 5,200 contours used to generate Fig. 3 was on the positive real axis between 0.5 and 2.

The construction of the grid includes the point $(0,0)$, which corresponds to $|A|=1$ and $|W|=1$ in the logarithmic space. Thus, the lowest point of the surface in Fig. 3 corresponds to the circular contour used by the FFT and the IFFT. The decimal logarithm of the numerical error in this case is −32.72. For comparison, for this point, the logarithm of the error computed using regular FFT followed by IFFT is −34.2. The difference is due to the fact that Algorithms 1 and 2 use FFT and IFFT multiple times, which increases the error.

### Algorithms that reverse the direction of the chirp contour

To improve the numerical stability, experiment 2 used the CZT-R and ICZT-R algorithms described in Supplementary Appendix G), which reverse the direction of the chirp contour when $|W| < 1$. These algorithms were not used in experiment 1 because all contours used in that experiment were decaying logarithmic spirals (i.e., blue contours), which don’t need to be reversed. Experiment 3 also used the CZT-R and ICZT-R algorithms.

### Third experiment

This experiment systematically varied the number of contour points and the size of the floating-point numbers used to compute the transforms. The results are summarized in Fig. 5, which has three sub-figures for 128, 256, and 512 bits, respectively. Each sub-figure contains 4 surfaces, which correspond to $M=64$, 128, 256, and 512. The lowest surface in Fig. 5a is the same as the surface shown in Fig. 3. All surfaces in all sub-figures were computed using the same discretization of the parameters *A* and *W* that was used in the second experiment. For each surface, the figure shows only the subset of points for which the numerical error does not exceed the magnitude of the unit-length input vector. That is, vertical values above 0 on the logarithmic scale are not shown.

The vertical coordinate of each point in Fig. 5 was computed by averaging the numerical error for 10 unit-length input vectors. The lowest points of the nested surfaces in each sub-figure are very close to each other and are slightly above the machine epsilon for the corresponding floating-point precision. Once again, these points correspond to the circular chirp contours used by the FFT and the IFFT. All three axes in each sub-figure are scaled logarithmically. The units on the vertical axes are different for each of the three sub-figures.

### Chirp contours

In all experiments, a chirp contour is defined as a logarithmic spiral that spans a 360° arc. To preserve the analogy with the FFT and the IFFT, the transform parameter *W* was selected such that doubling *M* keeps the previous contour points the same and distributes the new points between them. More specifically, going from a contour with *M* points to a contour with 2*M* points is accomplished by keeping the original *M* points intact, inserting $M-1$ new points in the middle of each angular interval and adding 1 last point in the middle of the angular interval between the previous end point and the start point. The start point of the contour is equal to *A*. The last point is given by $A{W}^{-(M-\mathrm{1)}}$. An example contour is shown in Fig. 2.

Unlike the start point, which is always fixed, the last point depends on the value of $M$. Because historically^4^ the points on the chirp contour were mapped to the z-transform (negative powers) and not to the power series (positive powers), the end point is assumed to be $A{W}^{-(M-\mathrm{1)}}$. To make it easier to relate points to parameter values, however, Figs 3 and 5 use ${\log }_{2}(|W{|}^{M})$. The reason the power is not $M-1$ is to ensure that there is a one-to-one mapping between chirp contours and grid points for different values of *M*. For example, a vertical line through all four surfaces in Fig. 5c maps to the same logarithmic spiral contour, even though each contour has a different number of points.

### Alternative CZT and ICZT implementation

The paper describes two alternative versions of the ICZT algorithm. The default version is shown in Algorithm 2. All results reported in the paper use this version or the modified version that reverses the chirp contour (see Supplementary Appendix G). The alternative version performs the Toeplitz–vector products on lines 25–28 using a different $O(n\,\log \,n)$ algorithm that is based on Pustylnikov’s decomposition^23,24^ (see Supplementary Appendix C). The results obtained with that algorithm are numerically very similar to those obtained with the default algorithm and are not reported in the paper.

The paper also describes two alternative versions of the CZT algorithm. Algorithm 1 is the default version. The alternative version replaces line 14 in Algorithm 1 with a call to ToeplitzMultiplyP, which uses Pustylnikov’s decomposition and is described in Supplementary Appendix C. The numerical performance of this algorithm is similar to the performance of the default CZT algorithm. These results are also not reported in the paper.

## Supplementary information

Supplementary Information

## Data Availability

The data sets that were collected in order to generate the figures in the paper and in the Supplementary Information are available from the corresponding author on reasonable request.
