# Supplementary material for: Generalizing the inverse FFT off the unit circle
Source: Sci Rep. 2019 Oct 8;9:14443. doi: 10.1038/s41598-019-50234-9 (PMC6783538; doi:10.1038/s41598-019-50234-9)
Supplement: Supplementary file 1 — Supplementary Information [file 41598_2019_50234_MOESM1_ESM.pdf]

Supplementary Information for "Generalizing the inverse FFT off the unit circle"

Vladimir Sukhoy[1] & Alexander Stoytchev[1,*]

Preliminaries

The supplementary information consists of nine appendices that provide additional theoretical proofs, algorithms, and numerical error models for the CZT and the ICZT algorithms. The main paper refers to each appendix using its assigned letter, e.g., Supplementary Appendix B.

The supplementary information also contains 21 theorems, lemmas, and corollaries. The same index counter is used for all three categories, e.g., Lemma 6 comes after Theorem 5, even though Lemma 6 is the first lemma in the text. The numbering starts at 1 because there are no theorems, lemmas, or corollaries in the main paper. To improve readability and to avoid confusion, the numbering of the formulas continues uninterrupted from the main paper.

The appendices and the main text refer to different types of structured matrices. Figure S1 illustrates the general shape of these matrices and shows their generating vectors.

Summary of the Supplementary Appendices

Appendix A gives an example with a 3×3 CZT and ICZT. The example uses expanded matrix notation to visualize the structured matrix equations that describe the CZT and the ICZT. It also shows how the inverse matrix $\hat{\boldsymbol{W}}^{-1}$ can be expressed as the difference between two products of triangular Toeplitz matrices.

Appendix B describes a popular method for multiplying a Toeplitz matrix by a vector in $O(n \log n)$ time that uses circulant matrix embedding. This is the default approach that is used by both the CZT algorithm and the ICZT algorithm.

Appendix C describes another method for multiplying a Toeplitz matrix by a vector that uses Pustylnikov's formula. It also runs in $O(n \log n)$ time and can be used to implement alternative versions of the CZT and the ICZT algorithms.

Appendix D shows how to multiply a circulant matrix by a vector in $O(n \log n)$ time using FFT and IFFT. It also shows how to multiply a skew-circulant matrix by a vector in $O(n \log n)$ time. These subroutines are used by the Toeplitz–vector multiplication algorithms in Appendices B and C.

Appendix E shows how to express the inverse of a symmetric Toeplitz matrix. It proves a special case of the Gohberg–Semencul formula, which uses only one generating vector instead of two vectors. It also proves that this generating vector, $\mathbf{u}$, is equal to the first column of the inverse matrix $\hat{\boldsymbol{W}}^{-1}$.

Appendix F proves the formula for the elements of the generating vector $\mathbf{u}$. This formula and the results from the previous appendix are used by the ICZT algorithm.

Appendix G describes alternative versions of the CZT and the ICZT algorithms that have improved numerical stability for chirp contours that are growing logarithmic spirals. These versions should be used in all cases when the transform parameter W has a magnitude that is less than 1, i.e., $|W| < 1$. Depending on the values of M, A, and W, these algorithms can reduce the numerical error by several orders of magnitude. Experimental results that confirm these findings are also included in this appendix.

Appendix H proves how changing the polar angle of the transform parameter A affects the polar angles of all elements in the CZT input vector $\mathbf{x}$. In addition, it shows how a change in A affects the polar angles of the elements in the ICZT output vector $\mathbf{x}$. This appendix also proves that these changes do not affect the norm of $\mathbf{x}$, which was used to simplify the experimental design described in the Methods section.

Appendix I gives approximation formulas for the absolute numerical error of the CZT and the ICZT algorithms. It also gives error formulas for their sequential application, i.e., CZT followed by ICZT or ICZT followed by CZT. Empirical results that confirm these formulas are also provided in this appendix.

[1] Department of Electrical and Computer Engineering, Iowa State University, Ames, IA 50011, USA. Correspondence and requests for materials should be addressed to A.S. (email: alexs@iastate.edu).

Matrix Type	Matrix Shape	Generating Vector(s)
Diagonal	$\begin{bmatrix} a & 0 & 0 \\ 0 & b & 0 \\ 0 & 0 & c \end{bmatrix}$	$\mathbf{d} = (a, b, c)$
Toeplitz	$\begin{bmatrix} a & b & c \\ d & a & b \\ e & d & a \end{bmatrix}$	$\mathbf{r} = (a, b, c)$<br>$\mathbf{c} = (a, d, e)$
Upper-Triangular Toeplitz	$\begin{bmatrix} a & b & c \\ 0 & a & b \\ 0 & 0 & a \end{bmatrix}$	$\mathbf{r} = (a, b, c)$<br>$\mathbf{c} = (a, 0, 0)$
Lower-Triangular Toeplitz	$\begin{bmatrix} a & 0 & 0 \\ d & a & 0 \\ e & d & a \end{bmatrix}$	$\mathbf{r} = (a, 0, 0)$<br>$\mathbf{c} = (a, d, e)$
Symmetric Toeplitz	$\begin{bmatrix} a & b & c \\ b & a & b \\ c & b & a \end{bmatrix}$	$\mathbf{r} = (a, b, c)$<br>$\mathbf{c} = (a, b, c)$
Circulant	$\begin{bmatrix} a & c & b \\ b & a & c \\ c & b & a \end{bmatrix}$	$\mathbf{r} = (a, c, b)$<br>or<br>$\mathbf{c} = (a, b, c)$
Skew-Circulant	$\begin{bmatrix} a & -c & -b \\ b & a & -c \\ c & b & a \end{bmatrix}$	$\mathbf{r} = (a, -c, -b)$<br>or<br>$\mathbf{c} = (a, \quad b, \quad c)$
Vandermonde	$\begin{bmatrix} a^0 & a^1 & a^2 \\ b^0 & b^1 & b^2 \\ c^0 & c^1 & c^2 \end{bmatrix}$	$\mathbf{v} = (a, b, c)$

Figure S1. Illustration of the matrix shapes and the generating vector(s) for the different types of structured matrices used in this paper. The diagonal matrix requires only one vector, i.e., the main diagonal $\mathbf{d}$. The Toeplitz matrix and most other matrices shown here can be generated using their first row, $\mathbf{r}$, and their first column, $\mathbf{c}$. The upper-triangular Toeplitz matrix can be fully described using only its first row, but keeping both $\mathbf{r}$ and $\mathbf{c}$ makes it possible to use algorithms intended for a more general Toeplitz matrix. Similarly, the lower-triangular Toeplitz matrix can be described using only its first column, but the algorithms described here use both $\mathbf{r}$ and $\mathbf{c}$. This also applies to the symmetric Toeplitz matrix, for which $\mathbf{r} = \mathbf{c}$. The circulant and the skew-circulant matrices can be specified using either their first row or their first column, which leads to alternative versions of the algorithms (here only $\mathbf{c}$ is used). The Vandermonde matrix can be generated using only one vector, $\mathbf{v}$, that is equal to its second column. This is possible because each row of the matrix is a (different) geometric progression that starts from 1, i.e., each element of the generating vector specifies the common ratio for the corresponding geometric progression. The DFT matrix (not shown) is another structured matrix that is a special case of a Vandermonde matrix (see Appendix D for an example).

SUPPLEMENTARY APPENDIX A: EXAMPLE

In the 3-by-3 case, the CZT can be expressed with the following matrix equation:

$$\underbrace{\begin{bmatrix} \mathrm{X}_0 \\ \mathrm{X}_1 \\ \mathrm{X}_2 \end{bmatrix}}_{\mathbf{X}} = \underbrace{\begin{bmatrix} 1 & 1 & 1 \\ 1 & W^1 & W^2 \\ 1 & W^2 & W^4 \end{bmatrix}}_{\boldsymbol{W}} \underbrace{\begin{bmatrix} A^{-0} & 0 & 0 \\ 0 & A^{-1} & 0 \\ 0 & 0 & A^{-2} \end{bmatrix}}_{\mathbf{A}} \underbrace{\begin{bmatrix} \mathrm{x}_0 \\ \mathrm{x}_1 \\ \mathrm{x}_2 \end{bmatrix}}_{\mathbf{x}}. \tag{20}$$

Using Bluestein's substitution[5], we can express the Vandermonde matrix $\boldsymbol{W}$ as a product of a diagonal matrix $\mathbf{P}$, a Toeplitz matrix $\hat{\boldsymbol{W}}$, and another diagonal matrix $\mathbf{Q}$, i.e.,

$$\underbrace{\begin{bmatrix} \mathrm{X}_0 \\ \mathrm{X}_1 \\ \mathrm{X}_2 \end{bmatrix}}_{\mathbf{X}} = \underbrace{\begin{bmatrix} W^{\frac{0^2}{2}} & 0 & 0 \\ 0 & W^{\frac{1^2}{2}} & 0 \\ 0 & 0 & W^{\frac{2^2}{2}} \end{bmatrix}}_{\mathbf{P}} \underbrace{\begin{bmatrix} 1 & W^{-\frac{1}{2}} & W^{-2} \\ W^{-\frac{1}{2}} & 1 & W^{-\frac{1}{2}} \\ W^{-2} & W^{-\frac{1}{2}} & 1 \end{bmatrix}}_{\hat{\boldsymbol{W}}} \underbrace{\begin{bmatrix} W^{\frac{0^2}{2}} & 0 & 0 \\ 0 & W^{\frac{1^2}{2}} & 0 \\ 0 & 0 & W^{\frac{2^2}{2}} \end{bmatrix}}_{\mathbf{Q}} \underbrace{\begin{bmatrix} A^{-0} & 0 & 0 \\ 0 & A^{-1} & 0 \\ 0 & 0 & A^{-2} \end{bmatrix}}_{\mathbf{A}} \underbrace{\begin{bmatrix} \mathrm{x}_0 \\ \mathrm{x}_1 \\ \mathrm{x}_2 \end{bmatrix}}_{\mathbf{x}}. \tag{21}$$

In this case, the expanded matrix equation for the ICZT looks like this:

$$\underbrace{\begin{bmatrix} \mathrm{x}_0 \\ \mathrm{x}_1 \\ \mathrm{x}_2 \end{bmatrix}}_{\mathbf{x}} = \underbrace{\begin{bmatrix} A^{0} & 0 & 0 \\ 0 & A^{1} & 0 \\ 0 & 0 & A^{2} \end{bmatrix}}_{\mathbf{A}^{-1}} \underbrace{\begin{bmatrix} W^{-\frac{0^2}{2}} & 0 & 0 \\ 0 & W^{-\frac{1^2}{2}} & 0 \\ 0 & 0 & W^{-\frac{2^2}{2}} \end{bmatrix}}_{\mathbf{Q}^{-1}} \underbrace{\begin{bmatrix} \hat{\boldsymbol{W}}^{-1}_{1,1} & \hat{\boldsymbol{W}}^{-1}_{1,2} & \hat{\boldsymbol{W}}^{-1}_{1,3} \\ \hat{\boldsymbol{W}}^{-1}_{2,1} & \hat{\boldsymbol{W}}^{-1}_{2,2} & \hat{\boldsymbol{W}}^{-1}_{2,3} \\ \hat{\boldsymbol{W}}^{-1}_{3,1} & \hat{\boldsymbol{W}}^{-1}_{3,2} & \hat{\boldsymbol{W}}^{-1}_{3,3} \end{bmatrix}}_{\hat{\boldsymbol{W}}^{-1}} \underbrace{\begin{bmatrix} W^{-\frac{0^2}{2}} & 0 & 0 \\ 0 & W^{-\frac{1^2}{2}} & 0 \\ 0 & 0 & W^{-\frac{2^2}{2}} \end{bmatrix}}_{\mathbf{P}^{-1}} \underbrace{\begin{bmatrix} \mathrm{X}_0 \\ \mathrm{X}_1 \\ \mathrm{X}_2 \end{bmatrix}}_{\mathbf{X}}. \tag{22}$$

The inverse matrix $\hat{\boldsymbol{W}}^{-1}$ is given in Eq. (79). The ICZT algorithm, however, does not construct this matrix explicitly. As proven in Theorem 5, $\hat{\boldsymbol{W}}^{-1}$ can be expressed as follows:

$$\mathrm{u}_0\,\hat{\boldsymbol{W}}^{-1} = \underbrace{\begin{bmatrix} \mathrm{u}_0 & 0 & 0 \\ \mathrm{u}_1 & \mathrm{u}_0 & 0 \\ \mathrm{u}_2 & \mathrm{u}_1 & \mathrm{u}_0 \end{bmatrix}}_{\boldsymbol{\mathcal{A}}} \underbrace{\begin{bmatrix} \mathrm{u}_0 & \mathrm{u}_1 & \mathrm{u}_2 \\ 0 & \mathrm{u}_0 & \mathrm{u}_1 \\ 0 & 0 & \mathrm{u}_0 \end{bmatrix}}_{\boldsymbol{\mathcal{A}}^T} - \underbrace{\begin{bmatrix} 0 & 0 & 0 \\ \mathrm{u}_2 & 0 & 0 \\ \mathrm{u}_1 & \mathrm{u}_2 & 0 \end{bmatrix}}_{\boldsymbol{\mathcal{D}}^T} \underbrace{\begin{bmatrix} 0 & \mathrm{u}_2 & \mathrm{u}_1 \\ 0 & 0 & \mathrm{u}_2 \\ 0 & 0 & 0 \end{bmatrix}}_{\boldsymbol{\mathcal{D}}}, \tag{23}$$

where the generating vector $\mathbf{u} = (\mathrm{u}_0, \mathrm{u}_1, \mathrm{u}_2)$ is given by Eq. (78). For these values of the elements of $\mathbf{u}$, Eq. (23) has the following form:

$$\mathrm{u}_0\,\hat{\boldsymbol{W}}^{-1} = \underbrace{\begin{bmatrix} \frac{W^3}{(W-1)(W^2-1)} & 0 & 0 \\ -\frac{W^{\frac{3}{2}}}{(W-1)^2} & \frac{W^3}{(W-1)(W^2-1)} & 0 \\ \frac{W^2}{(W-1)(W^2-1)} & -\frac{W^{\frac{3}{2}}}{(W-1)^2} & \frac{W^3}{(W-1)(W^2-1)} \end{bmatrix}}_{\boldsymbol{\mathcal{A}}} \underbrace{\begin{bmatrix} \frac{W^3}{(W-1)(W^2-1)} & -\frac{W^{\frac{3}{2}}}{(W-1)^2} & \frac{W^2}{(W-1)(W^2-1)} \\ 0 & \frac{W^3}{(W-1)(W^2-1)} & -\frac{W^{\frac{3}{2}}}{(W-1)^2} \\ 0 & 0 & \frac{W^3}{(W-1)(W^2-1)} \end{bmatrix}}_{\boldsymbol{\mathcal{A}}^T}$$

$$- \underbrace{\begin{bmatrix} 0 & 0 & 0 \\ \frac{W^2}{(W-1)(W^2-1)} & 0 & 0 \\ -\frac{W^{\frac{3}{2}}}{(W-1)^2} & \frac{W^2}{(W-1)(W^2-1)} & 0 \end{bmatrix}}_{\boldsymbol{\mathcal{D}}^T} \underbrace{\begin{bmatrix} 0 & \frac{W^2}{(W-1)(W^2-1)} & -\frac{W^{\frac{3}{2}}}{(W-1)^2} \\ 0 & 0 & \frac{W^2}{(W-1)(W^2-1)} \\ 0 & 0 & 0 \end{bmatrix}}_{\boldsymbol{\mathcal{D}}} \tag{24}$$

The Toeplitz matrices $\boldsymbol{\mathcal{A}}$, $\boldsymbol{\mathcal{A}}^T$, $\boldsymbol{\mathcal{D}}^T$, and $\boldsymbol{\mathcal{D}}$ are not constructed explicitly either. Instead, the vector $\mathbf{x}$ in Eq. (22), where $\hat{\boldsymbol{W}}^{-1}$ is expressed as in Eq. (24), is computed in $O(n \log n)$ time by exploiting the structure of these matrices.

Supplementary Appendix B: Computing a Toeplitz–Vector Product Using Circulant Matrix Embedding

This appendix describes a popular method for multiplying a square Toeplitz matrix by a vector[22] (see p. 202). This method can be extended to work with rectangular Toeplitz matrices as well. This is the default method that is used by both the CZT algorithm and the ICZT algorithm described in the main paper.

A. Example with $M = N = 3$

Let $\mathbf{T}$ be a 3-by-3 Toeplitz matrix that is generated by its first row $\mathbf{r} = (r_0, r_1, r_2)$ and its first column $\mathbf{c} = (c_0, c_1, c_2)$, where $r_0 = c_0$. We wish to compute the product of $\mathbf{T}$ with the vector $\mathbf{x} = (x_0, x_1, x_2)^T$, i.e.,

$$\mathbf{T}\,\mathbf{x} = \begin{bmatrix} c_0 & r_1 & r_2 \\ c_1 & c_0 & r_1 \\ c_2 & c_1 & c_0 \end{bmatrix} \begin{bmatrix} x_0 \\ x_1 \\ x_2 \end{bmatrix} = \begin{bmatrix} y_0 \\ y_1 \\ y_2 \end{bmatrix} = \mathbf{y}. \tag{25}$$

To do this, we start by embedding the matrix $\mathbf{T}$ into an 8-by-8 circulant matrix $\hat{\mathbf{T}}$ (which is also a Toeplitz matrix). The vector $\mathbf{x}$ is padded with five zeros at the end, which results in an eight-element vector $\hat{\mathbf{x}}$. Then, the vector $\mathbf{y}$ is equal to the first three elements of $\hat{\mathbf{y}} = \hat{\mathbf{T}}\,\hat{\mathbf{x}}$. More formally,

$$\hat{\mathbf{T}}\,\hat{\mathbf{x}} = \left[\begin{array}{ccc|ccccc} c_0 & r_1 & r_2 & 0 & 0 & 0 & c_2 & c_1 \\ c_1 & c_0 & r_1 & r_2 & 0 & 0 & 0 & c_2 \\ c_2 & c_1 & c_0 & r_1 & r_2 & 0 & 0 & 0 \\ \hline 0 & c_2 & c_1 & c_0 & r_1 & r_2 & 0 & 0 \\ 0 & 0 & c_2 & c_1 & c_0 & r_1 & r_2 & 0 \\ 0 & 0 & 0 & c_2 & c_1 & c_0 & r_1 & r_2 \\ r_2 & 0 & 0 & 0 & c_2 & c_1 & c_0 & r_1 \\ r_1 & r_2 & 0 & 0 & 0 & c_2 & c_1 & c_0 \end{array}\right] \begin{bmatrix} x_0 \\ x_1 \\ x_2 \\ 0 \\ 0 \\ 0 \\ 0 \\ 0 \end{bmatrix} = \begin{bmatrix} \hat{y}_0 \\ \hat{y}_1 \\ \hat{y}_2 \\ \hat{y}_3 \\ \hat{y}_4 \\ \hat{y}_5 \\ \hat{y}_6 \\ \hat{y}_7 \end{bmatrix} = \begin{bmatrix} y_0 \\ y_1 \\ y_2 \\ \boxtimes \\ \boxtimes \\ \boxtimes \\ \boxtimes \\ \boxtimes \end{bmatrix} \begin{matrix} \left.\begin{matrix} \\ \\ \\ \end{matrix}\right\} \mathbf{y} \\ \\ \\ \\ \\ \\ \end{matrix} \tag{26}$$

The circulant matrix $\hat{\mathbf{T}}$ is generated by its first column $\hat{\mathbf{c}}$, where $\hat{\mathbf{c}} = (c_0, c_1, c_2, 0, 0, 0, r_2, r_1)$. The number of zeros inserted after c_2 is equal to $8 - 5 = 3$, where 8 is the size of the matrix $\hat{\mathbf{T}}$ and 5 is the number of free parameters in $\mathbf{r}$ and $\mathbf{c}$ ($5 = 3 + 3 - 1$, because $r_0 = c_0$). Given $\hat{\mathbf{c}}$ and $\hat{\mathbf{x}}$, the vector $\hat{\mathbf{y}} = \hat{\mathbf{T}}\,\hat{\mathbf{x}}$ can be computed efficiently as described below. The last five elements of $\hat{\mathbf{y}}$ are computed but are then discarded, which is indicated with $\boxtimes$ in Eq. (26).

B. The General Case for a Rectangular Toeplitz Matrix

Let $\mathbf{T}$ be an M-by-N Toeplitz matrix that is generated by its first row $\mathbf{r} = (r_0, r_1, r_2, \ldots, r_{N-2}, r_{N-1})$ and its first column $\mathbf{c} = (c_0, c_1, c_2, \ldots, c_{M-2}, c_{M-1})$, assuming that $r_0 = c_0$. Also, let $\mathbf{x}$ be a column vector of length N.

To compute $\mathbf{y} = \mathbf{T}\,\mathbf{x}$, we start by embedding $\mathbf{T}$ into an n-by-n circulant matrix $\hat{\mathbf{T}}$, where $n = 2^{\lceil \log_2(M+N-1) \rceil}$. This matrix is generated by its first column $\hat{\mathbf{c}}$, which has n elements that are defined as follows:

$$\hat{\mathbf{c}} = (c_0, c_1, \ldots, c_{M-1}, 0, 0, \ldots, 0, r_{N-1}, \ldots, r_2, r_1). \tag{27}$$

Note that r_0, which is equal to c_0, is not used in this formula. The number of zeros inserted in $\hat{\mathbf{c}}$ between c_{M-1} and r_{N-1} is equal to $n - (M+N-1)$. The vector $\mathbf{x}$ is padded with $n-N$ zeros at the end, which results in a vector $\hat{\mathbf{x}}$ of length n. Given $\hat{\mathbf{c}}$ and $\hat{\mathbf{x}}$, the product $\hat{\mathbf{y}} = \hat{\mathbf{T}}\,\hat{\mathbf{x}}$ can be computed in $O(n \log n)$ time using two FFTs and one IFFT (see Algorithm S4 in Appendix D).

Because the original matrix $\mathbf{T}$ appears as a sub-matrix of $\hat{\mathbf{T}}$ in its upper-left corner and because $\mathbf{x}$ is padded with the appropriate number of zeros, the first M elements of $\hat{\mathbf{y}}$ are equal to $\mathbf{y}$, i.e., $y_k = \hat{y}_k$ for each $k \in \{0, 1, 2, \ldots, M-1\}$.

To summarize, the product of an M-by-N Toeplitz matrix $\mathbf{T}$ and a vector $\mathbf{x}$ of length N can be computed using the following four steps: 1) go from the Toeplitz matrix $\mathbf{T}$, which is generated by the vectors $\mathbf{r}$ and $\mathbf{c}$, to the circulant matrix $\hat{\mathbf{T}}$ by forming its first column $\hat{\mathbf{c}}$ using Eq. (27); 2) pad the vector $\mathbf{x}$ with $n - N$ zeros at the end to get the vector $\hat{\mathbf{x}}$; 3) pass $\hat{\mathbf{c}}$ and $\hat{\mathbf{x}}$ to Algorithm S4, which computes $\hat{\mathbf{y}}$; and 4) set $\mathbf{y}$ to the first M elements of $\hat{\mathbf{y}}$. Note that neither the Toeplitz matrix $\mathbf{T}$ nor the circulant matrix $\hat{\mathbf{T}}$ is constructed explicitly; only their generating vectors are used to perform the necessary computations.

Algorithm S1 gives the pseudo-code for the procedure described above. The computational complexity of this algorithm is $O(n \log n)$. The function name is ToeplitzMultiplyE, where 'E' abbreviates circulant 'embedding'. Algorithm S2 gives the pseudo-code for the helper function ZeroPad that pads an array with zeros at the end such that the new length of the array is equal to n. This function is used in the next appendix as well.

There are several variations of the circulant embedding procedure that are equivalent to the one described above. In some of the other versions the first column $\hat{\mathbf{c}}$ of $\hat{\mathbf{T}}$ is equal to a cyclic shift of the elements of the vector $\hat{\mathbf{c}}$ that is defined in Eq. (27). For example, Pan[26] (see p. 66) constructs $\hat{\mathbf{c}}$ as follows:

$$\hat{\mathbf{c}} = (r_{N-1}, \ldots, r_2, r_1, c_0, c_1, \ldots, c_{M-1}, 0, 0, \ldots, 0). \tag{28}$$

In this case, the $n - (M+N-1)$ zeros are inserted at the end. The vector $\mathbf{x}$ is still padded with $n-N$ zeros at the end. However, the result vector $\mathbf{y}$ is extracted from $\hat{\mathbf{y}}$ as follows:

$$\mathbf{y} = (\hat{y}_{N-1}, \hat{y}_N, \hat{y}_{N+1}, \ldots, \hat{y}_{N+M-2}). \tag{29}$$

For example, if $M = N = 3$, then Eq. (26) could be replaced with the following expression:

$$\hat{\mathbf{T}}\,\hat{\mathbf{x}} = \left[\begin{array}{ccc|ccccc} r_2 & 0 & 0 & 0 & c_2 & c_1 & c_0 & r_1 \\ r_1 & r_2 & 0 & 0 & 0 & c_2 & c_1 & c_0 \\ \hline c_0 & r_1 & r_2 & 0 & 0 & 0 & c_2 & c_1 \\ c_1 & c_0 & r_1 & r_2 & 0 & 0 & 0 & c_2 \\ c_2 & c_1 & c_0 & r_1 & r_2 & 0 & 0 & 0 \\ \hline 0 & c_2 & c_1 & c_0 & r_1 & r_2 & 0 & 0 \\ 0 & 0 & c_2 & c_1 & c_0 & r_1 & r_2 & 0 \\ 0 & 0 & 0 & c_2 & c_1 & c_0 & r_1 & r_2 \end{array}\right] \begin{bmatrix} x_0 \\ x_1 \\ x_2 \\ 0 \\ 0 \\ 0 \\ 0 \\ 0 \end{bmatrix} = \begin{bmatrix} \hat{y}_0 \\ \hat{y}_1 \\ \hat{y}_2 \\ \hat{y}_3 \\ \hat{y}_4 \\ \hat{y}_5 \\ \hat{y}_6 \\ \hat{y}_7 \end{bmatrix} = \begin{bmatrix} \boxtimes \\ \boxtimes \\ y_0 \\ y_1 \\ y_2 \\ \boxtimes \\ \boxtimes \\ \boxtimes \end{bmatrix} \begin{matrix} \\ \\ \left.\begin{matrix} \\ \\ \\ \end{matrix}\right\} \mathbf{y} \\ \\ \\ \\ \end{matrix} \tag{30}$$

The original matrix $\mathbf{T}$ is still embedded in $\hat{\mathbf{T}}$ (indicated with the highlighted region), but it is no longer in the upper-left corner. Similarly, the result vector $\mathbf{y}$, which is of length M, starts at offset $N-1 = 2$ in the vector $\hat{\mathbf{y}}$.

Algorithm S1. Multiply a Toeplitz matrix by a vector using circulant embedding. Runs in $O(n \log n)$ time.

TOEPLITZMULTIPLYE($\mathbf{r}$, $\mathbf{c}$, $\mathbf{x}$)
// Compute the product $\mathbf{y} = \mathbf{T}\mathbf{x}$ of a Toeplitz matrix $\mathbf{T}$
// and a vector $\mathbf{x}$, where $\mathbf{T}$ is specified by its first row
// $\mathbf{r} = (\mathrm{r}[0], \mathrm{r}[1], \mathrm{r}[2], \ldots, \mathrm{r}[N-1])$ and its first column
// $\mathbf{c} = (\mathrm{c}[0], \mathrm{c}[1], \mathrm{c}[2], \ldots, \mathrm{c}[M-1])$, where $\mathrm{r}[0] = \mathrm{c}[0]$.
$N \leftarrow$ LENGTH($\mathbf{r}$);
$M \leftarrow$ LENGTH($\mathbf{c}$);
ASSERT($\mathrm{r}[0] = \mathrm{c}[0]$);
ASSERT(LENGTH($\mathbf{x}$) $= N$);
$n \leftarrow 2^{\lceil \log_2(M+N-1) \rceil}$;
// Form an array $\hat{\mathbf{c}}$ by concatenating $\mathbf{c}$, $n - (M+N-1)$
// zeros, and the reverse of the last $N-1$ elements of $\mathbf{r}$.
$\hat{\mathbf{c}} \leftarrow$ ZEROARRAY(n);
for $k \leftarrow 0$ **to** $M-1$ **do**
    $\hat{\mathrm{c}}[k] \leftarrow \mathrm{c}[k]$;
end for
for $k \leftarrow 1$ **to** $N-1$ **do**
    $\hat{\mathrm{c}}[n-k] \leftarrow \mathrm{r}[k]$;
end for
// $\hat{\mathbf{c}} = (\mathrm{c}[0], \mathrm{c}[1], \ldots, \mathrm{c}[M-1], 0, \ldots, 0, \mathrm{r}[N-1], \ldots, \mathrm{r}[2], \mathrm{r}[1])$;
$\hat{\mathbf{x}} \leftarrow$ ZEROPAD($\mathbf{x}, n$); // call Algorithm S2
$\hat{\mathbf{y}} \leftarrow$ CIRCULANTMULTIPLY($\hat{\mathbf{c}}$, $\hat{\mathbf{x}}$); // call Algorithm S4
// The result is the first M elements of $\hat{\mathbf{y}}$.
$\mathbf{y} \leftarrow$ EMPTYARRAY(M);
for $k \leftarrow 0$ **to** $M - 1$ **do**
    $\mathrm{y}[k] \leftarrow \hat{\mathrm{y}}[k]$;
end for
return $\mathbf{y}$;

Algorithm S2. Pad an array to length n by appending zeros.

ZEROPAD($\mathbf{x}$, n)
$m \leftarrow$ LENGTH($\mathbf{x}$);
ASSERT($m \leq n$);
$\hat{\mathbf{x}} \leftarrow$ EMPTYARRAY(n);
for $k \leftarrow 0$ **to** $m - 1$ **do**
    $\hat{\mathrm{x}}[k] \leftarrow \mathrm{x}[k]$;
end for
for $k \leftarrow m$ **to** $n - 1$ **do**
    $\hat{\mathrm{x}}[k] \leftarrow 0$;
end for
return $\hat{\mathbf{x}}$;

SUPPLEMENTARY APPENDIX C: COMPUTING A TOEPLITZ–VECTOR PRODUCT USING PUSTYLNIKOV'S DECOMPOSITION

This appendix describes another algorithm for multiplying a Toeplitz matrix by a vector that uses Pustylnikov's decomposition[23,24] (see also pages 40 and 66 in references 25 and 26, respectively). This method can also be used by the CZT and the ICZT algorithms described in the main paper.

A. Example with $M = N = 3$

Let $\mathbf{c} = (\mathrm{c}_0, \mathrm{c}_1, \mathrm{c}_2)$ be the first column and $\mathbf{r} = (\mathrm{r}_0, \mathrm{r}_1, \mathrm{r}_2)$ be the first row of a 3-by-3 Toeplitz matrix $\mathbf{T}$, where $\mathrm{c}_0 = \mathrm{r}_0$. Once again, we wish to compute the product of $\mathbf{T}$ with the vector $\mathbf{x} = (\mathrm{x}_0, \mathrm{x}_1, \mathrm{x}_2)^T$, i.e.,

$$\mathbf{T}\,\mathbf{x} = \begin{bmatrix} \mathrm{c}_0 & \mathrm{r}_1 & \mathrm{r}_2 \\ \mathrm{c}_1 & \mathrm{c}_0 & \mathrm{r}_1 \\ \mathrm{c}_2 & \mathrm{c}_1 & \mathrm{c}_0 \end{bmatrix} \begin{bmatrix} \mathrm{x}_0 \\ \mathrm{x}_1 \\ \mathrm{x}_2 \end{bmatrix} = \begin{bmatrix} \mathrm{y}_0 \\ \mathrm{y}_1 \\ \mathrm{y}_2 \end{bmatrix} = \mathbf{y}. \tag{31}$$

The matrix $\mathbf{T}$ is square, but the number of its rows, or columns, is not a power of 2. Thus, the first step is to pad $\mathbf{T}$ and to embed it into a larger 4-by-4 Toeplitz matrix $\hat{\mathbf{T}}$. The matrix $\hat{\mathbf{T}}$ is generated by its first column and its first row, which can be derived from the first column $\mathbf{c}$ of $\mathbf{T}$ by padding it with a single zero and from the first row $\mathbf{r}$ of $\mathbf{T}$ by padding it with a single zero as well. The vector $\mathbf{x}$ is also padded with a zero at the end to produce the vector $\hat{\mathbf{x}}$, which is of size 4. This padding transforms Eq. (31) as follows:

$$\hat{\mathbf{T}}\,\hat{\mathbf{x}} = \left[\begin{array}{ccc|c} \mathrm{c}_0 & \mathrm{r}_1 & \mathrm{r}_2 & 0 \\ \mathrm{c}_1 & \mathrm{c}_0 & \mathrm{r}_1 & \mathrm{r}_2 \\ \mathrm{c}_2 & \mathrm{c}_1 & \mathrm{c}_0 & \mathrm{r}_1 \\ \hline 0 & \mathrm{c}_2 & \mathrm{c}_1 & \mathrm{c}_0 \end{array}\right] \begin{bmatrix} \mathrm{x}_0 \\ \mathrm{x}_1 \\ \mathrm{x}_2 \\ 0 \end{bmatrix} = \begin{bmatrix} \hat{\mathrm{y}}_0 \\ \hat{\mathrm{y}}_1 \\ \hat{\mathrm{y}}_2 \\ \hat{\mathrm{y}}_3 \end{bmatrix} = \begin{bmatrix} \mathrm{y}_0 \\ \mathrm{y}_1 \\ \mathrm{y}_2 \\ \boxtimes \end{bmatrix} \Bigg\} \mathbf{y} \tag{32}$$

The padding is necessary because the multiplication algorithm relies on FFT and IFFT, which in this text are described as recursive algorithms that expect the size of their input vector to be a power of 2.

The next step uses Pustylnikov's decomposition to express the padded Toeplitz matrix $\hat{\mathbf{T}}$ as the sum of two matrices $\hat{\mathbf{T}}'$ and $\hat{\mathbf{T}}''$, which are defined as follows:

$$\hat{\mathbf{T}}' = \begin{bmatrix} \mathrm{c}'_0 & \mathrm{c}'_3 & \mathrm{c}'_2 & \mathrm{c}'_1 \\ \mathrm{c}'_1 & \mathrm{c}'_0 & \mathrm{c}'_3 & \mathrm{c}'_2 \\ \mathrm{c}'_2 & \mathrm{c}'_1 & \mathrm{c}'_0 & \mathrm{c}'_3 \\ \mathrm{c}'_3 & \mathrm{c}'_2 & \mathrm{c}'_1 & \mathrm{c}'_0 \end{bmatrix} = \frac{1}{2} \begin{bmatrix} \mathrm{c}_0 & 0+\mathrm{r}_1 & \mathrm{c}_2+\mathrm{r}_2 & \mathrm{c}_1+0 \\ \mathrm{c}_1+0 & \mathrm{c}_0 & 0+\mathrm{r}_1 & \mathrm{c}_2+\mathrm{r}_2 \\ \mathrm{c}_2+\mathrm{r}_2 & \mathrm{c}_1+0 & \mathrm{c}_0 & 0+\mathrm{r}_1 \\ 0+\mathrm{r}_1 & \mathrm{c}_2+\mathrm{r}_2 & \mathrm{c}_1+0 & \mathrm{c}_0 \end{bmatrix}, \tag{33}$$

$$\hat{\mathbf{T}}'' = \begin{bmatrix} \mathrm{c}''_0 & -\mathrm{c}''_3 & -\mathrm{c}''_2 & -\mathrm{c}''_1 \\ \mathrm{c}''_1 & \mathrm{c}''_0 & -\mathrm{c}''_3 & -\mathrm{c}''_2 \\ \mathrm{c}''_2 & \mathrm{c}''_1 & \mathrm{c}''_0 & -\mathrm{c}''_3 \\ \mathrm{c}''_3 & \mathrm{c}''_2 & \mathrm{c}''_1 & \mathrm{c}''_0 \end{bmatrix} = \frac{1}{2} \begin{bmatrix} \mathrm{c}_0 & \mathrm{r}_1-0 & \mathrm{r}_2-\mathrm{c}_2 & 0-\mathrm{c}_1 \\ \mathrm{c}_1-0 & \mathrm{c}_0 & \mathrm{r}_1-0 & \mathrm{r}_2-\mathrm{c}_2 \\ \mathrm{c}_2-\mathrm{r}_2 & \mathrm{c}_1-0 & \mathrm{c}_0 & \mathrm{r}_1-0 \\ 0-\mathrm{r}_1 & \mathrm{c}_2-\mathrm{r}_2 & \mathrm{c}_1-0 & \mathrm{c}_0 \end{bmatrix}. \tag{34}$$

The matrix $\hat{\mathbf{T}}'$ is circulant. The matrix $\hat{\mathbf{T}}''$ is skew-circulant[28]. Some authors[26,27] use the term f-circulant with $f = -1$ to refer to a skew-circulant matrix.

Using these two matrices, Eq. (32) can be computed in two steps as follows:

$$\hat{\mathbf{T}}\,\hat{\mathbf{x}} = \hat{\mathbf{T}}'\,\hat{\mathbf{x}} + \hat{\mathbf{T}}''\,\hat{\mathbf{x}}$$
$$= \begin{bmatrix} \mathrm{c}'_0 & \mathrm{c}'_3 & \mathrm{c}'_2 & \mathrm{c}'_1 \\ \mathrm{c}'_1 & \mathrm{c}'_0 & \mathrm{c}'_3 & \mathrm{c}'_2 \\ \mathrm{c}'_2 & \mathrm{c}'_1 & \mathrm{c}'_0 & \mathrm{c}'_3 \\ \mathrm{c}'_3 & \mathrm{c}'_2 & \mathrm{c}'_1 & \mathrm{c}'_0 \end{bmatrix} \begin{bmatrix} \mathrm{x}_0 \\ \mathrm{x}_1 \\ \mathrm{x}_2 \\ 0 \end{bmatrix} + \begin{bmatrix} \mathrm{c}''_0 & -\mathrm{c}''_3 & -\mathrm{c}''_2 & -\mathrm{c}''_1 \\ \mathrm{c}''_1 & \mathrm{c}''_0 & -\mathrm{c}''_3 & -\mathrm{c}''_2 \\ \mathrm{c}''_2 & \mathrm{c}''_1 & \mathrm{c}''_0 & -\mathrm{c}''_3 \\ \mathrm{c}''_3 & \mathrm{c}''_2 & \mathrm{c}''_1 & \mathrm{c}''_0 \end{bmatrix} \begin{bmatrix} \mathrm{x}_0 \\ \mathrm{x}_1 \\ \mathrm{x}_2 \\ 0 \end{bmatrix}. \tag{35}$$

Both $\hat{\mathbf{y}}' = \hat{\mathbf{T}}'\,\hat{\mathrm{x}}$ and $\hat{\mathbf{y}}'' = \hat{\mathbf{T}}''\,\hat{\mathrm{x}}$ can be computed efficiently as described below. The partial results $\hat{\mathbf{y}}'$ and $\hat{\mathbf{y}}''$ are then added to get the result vector $\mathbf{y}$, i.e.,

$$\begin{bmatrix} \hat{y}_0' \\ \hat{y}_1' \\ \hat{y}_2' \\ \hat{y}_3' \end{bmatrix} + \begin{bmatrix} \hat{y}_0'' \\ \hat{y}_1'' \\ \hat{y}_2'' \\ \hat{y}_3'' \end{bmatrix} = \begin{bmatrix} \hat{y}_0' + \hat{y}_0'' \\ \hat{y}_1' + \hat{y}_1'' \\ \hat{y}_2' + \hat{y}_2'' \\ \boxtimes \end{bmatrix} = \begin{bmatrix} y_0 \\ y_1 \\ y_2 \\ \boxtimes \end{bmatrix} \Big\} \mathbf{y} \tag{36}$$

The last element of $\hat{\mathbf{y}} = \hat{\mathbf{y}}' + \hat{\mathbf{y}}''$ is not needed (and is not computed), it appears only because of the padding.

B. The General Case for a Rectangular Toeplitz Matrix

In the general case, the matrix $\mathbf{T}$ is an M-by-N Toeplitz matrix, the vector $\mathbf{x}$ is of size N, and the result vector $\mathbf{y}$ is of size M. The matrix $\mathbf{T}$ is generated by its first column $\mathbf{c} = (c_0, c_1, \ldots, c_{M-1})$ and its first row $\mathbf{r} = (r_0, r_1, \ldots, r_{N-1})$, where it is assumed that $c_0 = r_0$.

The first step is to go from the matrix $\mathbf{T}$ to the matrix $\hat{\mathbf{T}}$ of size n-by-n, where $n = 2^{\lceil \log_2 \max(M,N) \rceil}$. That is, $\hat{\mathbf{T}}$ is a square Toeplitz matrix and n is a power of two. The first column of $\hat{\mathbf{T}}$ can be constructed by padding $\mathbf{c}$ with $n{-}M$ zeros. Similarly, the first row of $\hat{\mathbf{T}}$ can be constructed by padding $\mathbf{r}$ with $n{-}N$ zeros. The vector $\mathbf{x}$ is also padded with $n{-}N$ zeros to create the vector $\hat{\mathbf{x}}$.

Given the padded vectors $\mathbf{c}$ and $\mathbf{r}$, which are now both of length n, we can use Pustylnikov's formula to calculate the first column $\mathbf{c}' = (c_0', c_1', \ldots, c_{n-1}')$ of the circulant matrix $\hat{\mathbf{T}}'$ and the first column $\mathbf{c}'' = (c_0'', c_1'', \ldots, c_{n-1}'')$ of the skew-circulant matrix $\hat{\mathbf{T}}''$ as follows:

$$c_k' = \frac{1}{2}\begin{cases} c_0, & \text{if } k = 0, \\ c_k{+}r_{n-k}, & \text{if } k \in \{1, 2, \ldots, n-1\}, \end{cases} \tag{37}$$

$$c_k'' = \frac{1}{2}\begin{cases} c_0, & \text{if } k = 0, \\ c_k{-}r_{n-k}, & \text{if } k \in \{1, 2, \ldots, n-1\}. \end{cases} \tag{38}$$

In other words, $\hat{\mathbf{T}}'$ and $\hat{\mathbf{T}}''$ have the following form:

$$\hat{\mathbf{T}}' = \begin{bmatrix} c_0' & c_{n-1}' & c_{n-2}' & \cdots & c_2' & c_1' \\ c_1' & c_0' & c_{n-1}' & \cdots & c_3' & c_2' \\ c_2' & c_1' & c_0' & \cdots & c_4' & c_3' \\ \vdots & \vdots & \vdots & \ddots & \vdots & \vdots \\ c_{n-2}' & c_{n-3}' & c_{n-4}' & \cdots & c_0' & c_{n-1}' \\ c_{n-1}' & c_{n-2}' & c_{n-3}' & \cdots & c_1' & c_0' \end{bmatrix}, \tag{39}$$

$$\hat{\mathbf{T}}'' = \begin{bmatrix} c_0'' & -c_{n-1}'' & -c_{n-2}'' & \cdots & -c_2'' & -c_1'' \\ c_1'' & c_0'' & -c_{n-1}'' & \cdots & -c_3'' & -c_2'' \\ c_2'' & c_1'' & c_0'' & \cdots & -c_4'' & -c_3'' \\ \vdots & \vdots & \vdots & \ddots & \vdots & \vdots \\ c_{n-2}'' & c_{n-3}'' & c_{n-4}'' & \cdots & c_0'' & -c_{n-1}'' \\ c_{n-1}'' & c_{n-2}'' & c_{n-3}'' & \cdots & c_1'' & c_0'' \end{bmatrix}. \tag{40}$$

Similarly to the example above, the product $\hat{\mathbf{y}} = \hat{\mathbf{T}}\,\hat{\mathbf{x}}$ can be computed in two steps:

$$\hat{\mathbf{y}} = \hat{\mathbf{T}}\,\hat{\mathbf{x}} = (\hat{\mathbf{T}}' + \hat{\mathbf{T}}'')\,\hat{\mathbf{x}} = \hat{\mathbf{T}}'\,\hat{\mathbf{x}} + \hat{\mathbf{T}}''\,\hat{\mathbf{x}}. \tag{41}$$

The product $\hat{\mathbf{y}}' = \hat{\mathbf{T}}'\,\hat{\mathbf{x}}$ is computed in $O(n \log n)$ time using Algorithm S4. The product $\hat{\mathbf{y}}'' = \hat{\mathbf{T}}''\,\hat{\mathbf{x}}$ is also computed in $O(n \log n)$ time using Algorithm S7. Finally, the first M elements of $\hat{\mathbf{y}}'$ and $\hat{\mathbf{y}}''$ are added together to compute the result vector $\mathbf{y}$, i.e.,

$$\mathbf{y} = (\hat{y}_0' + \hat{y}_0'',\ \hat{y}_1' + \hat{y}_1'',\ \ldots,\ \hat{y}_{M-1}' + \hat{y}_{M-1}''). \tag{42}$$

Algorithm S3 gives the pseudo-code for the procedure described above. The function name is TOEPLITZMULTIPLYP, where 'P' abbreviates 'Pustylnikov'. The algorithm runs in $O(n \log n)$ time. Once again, neither $\hat{\mathbf{T}}'$ nor $\hat{\mathbf{T}}''$ is constructed explicitly; only the generating vectors $\mathbf{c}'$ and $\mathbf{c}''$ are computed, which correspond to their first columns. The last $n{-}M$ elements of $\hat{\mathbf{y}}'$ and $\hat{\mathbf{y}}''$ are computed, but they are not used to compute $\mathbf{y}$.

In contrast to the circulant embedding approach described in Appendix B, Algorithm S3 does not embed $\mathbf{T}$ into only one square matrix with $n = 2^{\lceil \log_2 (M+N-1) \rceil}$. Instead, it uses two square matrices, each with $n = 2^{\lceil \log_2 \max(M,N) \rceil}$.

Algorithm S3. Multiply a Toeplitz matrix by a vector using Pustylnikov's decomposition. Runs in $O(n \log n)$ time.

```
TOEPLITZMULTIPLYP(r, c, x)
// Compute the product y = T x of a Toeplitz matrix T
// and a vector x where T is specified by its first row
// r = (r[0], r[1], r[2], ..., r[N−1]) and its first column
// c = (c[0], c[1], c[2], ..., c[M−1]), where r[0] = c[0].
N ← LENGTH(r);
M ← LENGTH(c);
ASSERT(r[0] = c[0]);
ASSERT(LENGTH(x) = N);
n ← 2^⌈log2(max(M,N))⌉;
if N ≠ n then
    r ← ZEROPAD(r, n);
    x ← ZEROPAD(x, n);
end if
if M ≠ n then
    c ← ZEROPAD(c, n);
end if
c′ ← EMPTYARRAY(n);
c″ ← EMPTYARRAY(n);
c′[0] ← 0.5 · c[0];
c″[0] ← 0.5 · c[0];
for k ← 1 to n − 1 do
    c′[k] ← 0.5 · (c[k] + r[n − k]);
    c″[k] ← 0.5 · (c[k] − r[n − k]);
end for
y′ ← CIRCULANTMULTIPLY(c′, x);
y″ ← SKEWCIRCULANTMULTIPLY(c″, x);
y ← EMPTYARRAY(M);
for k ← 0 to M − 1 do
    y[k] ← y′[k] + y″[k];
end for
return y;
```

Supplementary Appendix D: Multiplying a Circulant or a Skew-Circulant Matrix by a Vector Using FFT and IFFT

The algorithm described in Appendix B relies on another algorithm for computing the product of a circulant matrix with a vector in $O(n \log n)$ time. In addition to this, the algorithm described in Appendix C needs to compute the product of a skew-circulant matrix with a vector in $O(n \log n)$ time. This appendix describes both of these algorithms for n-by-n matrices, where n is a power of two. It is assumed that appropriate padding has already been applied (i.e., see lines 10–21 in Algorithm S1 and lines 10–17 in Algorithm S3).

A. Multiplying a Circulant Matrix by a Vector

A circulant matrix is a square matrix with columns that are generated by successive circular shifts of its first column.

Let $\mathbf{G}$ be a 4-by-4 circulant matrix that is generated by its first column $\mathbf{c} = (\mathrm{c}_0, \mathrm{c}_1, \mathrm{c}_2, \mathrm{c}_3)$, i.e.,

$$\mathbf{G} = \begin{bmatrix} \mathrm{c}_0 & \mathrm{c}_3 & \mathrm{c}_2 & \mathrm{c}_1 \\ \mathrm{c}_1 & \mathrm{c}_0 & \mathrm{c}_3 & \mathrm{c}_2 \\ \mathrm{c}_2 & \mathrm{c}_1 & \mathrm{c}_0 & \mathrm{c}_3 \\ \mathrm{c}_3 & \mathrm{c}_2 & \mathrm{c}_1 & \mathrm{c}_0 \end{bmatrix}. \tag{43}$$

The matrix $\mathbf{G}$ can be diagonalized[28] (see p. 73) using the DFT matrix $\mathbf{F}$ and the inverse DFT matrix $\mathbf{F}^{-1}$. In the 4-by-4 case these two matrices have the following form:

$$\mathbf{F} = \begin{bmatrix} 1 & 1 & 1 & 1 \\ 1 & \omega & \omega^2 & \omega^3 \\ 1 & \omega^2 & \omega^4 & \omega^6 \\ 1 & \omega^3 & \omega^6 & \omega^9 \end{bmatrix} = \begin{bmatrix} 1 & 1 & 1 & 1 \\ 1 & -i & -1 & i \\ 1 & -1 & 1 & -1 \\ 1 & i & -1 & -i \end{bmatrix}, \tag{44}$$

$$\mathbf{F}^{-1} = \frac{1}{4} \begin{bmatrix} 1 & 1 & 1 & 1 \\ 1 & \omega^{-1} & \omega^{-2} & \omega^{-3} \\ 1 & \omega^{-2} & \omega^{-4} & \omega^{-6} \\ 1 & \omega^{-3} & \omega^{-6} & \omega^{-9} \end{bmatrix} = \frac{1}{4} \begin{bmatrix} 1 & 1 & 1 & 1 \\ 1 & i & -1 & -i \\ 1 & -1 & 1 & -1 \\ 1 & -i & -1 & i \end{bmatrix}, \tag{45}$$

where $\omega = \mathrm{e}^{-\frac{2\pi i}{4}} = \cos(-\frac{\pi}{2}) + i \sin(-\frac{\pi}{2}) = -i$. Both matrices are symmetric, i.e., each is equal to its own transpose: $\mathbf{F}^T = \mathbf{F}$ and $(\mathbf{F}^{-1})^T = \mathbf{F}^{-1}$.

The matrix $\mathbf{G}$ can be expressed, i.e., diagonalized, using the following product of three matrices:

$$\mathbf{G} = \mathbf{F}^{-1} \boldsymbol{\Lambda} \, \mathbf{F}. \tag{46}$$

The diagonal matrix $\boldsymbol{\Lambda}$ is defined as follows:

$$\boldsymbol{\Lambda} = \mathrm{diag}(\boldsymbol{\lambda}) = \begin{bmatrix} \lambda_0 & 0 & 0 & 0 \\ 0 & \lambda_1 & 0 & 0 \\ 0 & 0 & \lambda_2 & 0 \\ 0 & 0 & 0 & \lambda_2 \end{bmatrix}. \tag{47}$$

Furthermore, the vector $\boldsymbol{\lambda} = (\lambda_0, \lambda_1, \lambda_2, \lambda_3)^T$, which contains the eigenvalues of $\mathbf{G}$, is equal to the product of the DFT matrix $\mathbf{F}$ with the column vector $\mathbf{c}$, i.e., $\boldsymbol{\lambda} = \mathbf{Fc}$.

The expanded form of Eq. (46) is shown below:

$$\begin{aligned} \mathbf{G} &= \mathbf{F}^{-1} \boldsymbol{\Lambda} \, \mathbf{F} \\ &= \frac{1}{4} \begin{bmatrix} 1 & 1 & 1 & 1 \\ 1 & i & -1 & -i \\ 1 & -1 & 1 & -1 \\ 1 & -i & -1 & i \end{bmatrix} \begin{bmatrix} \lambda_0 & 0 & 0 & 0 \\ 0 & \lambda_1 & 0 & 0 \\ 0 & 0 & \lambda_2 & 0 \\ 0 & 0 & 0 & \lambda_3 \end{bmatrix} \begin{bmatrix} 1 & 1 & 1 & 1 \\ 1 & -i & -1 & i \\ 1 & -1 & 1 & -1 \\ 1 & i & -1 & -i \end{bmatrix}. \end{aligned} \tag{48}$$

The vector $\boldsymbol{\lambda}$ has the following structure:

$$\boldsymbol{\lambda} = \mathbf{Fc} = \begin{bmatrix} 1 & 1 & 1 & 1 \\ 1 & -i & -1 & i \\ 1 & -1 & 1 & -1 \\ 1 & i & -1 & -i \end{bmatrix} \begin{bmatrix} \mathrm{c}_0 \\ \mathrm{c}_1 \\ \mathrm{c}_2 \\ \mathrm{c}_3 \end{bmatrix} = \begin{bmatrix} \mathrm{c}_0 + \mathrm{c}_1 + \mathrm{c}_2 + \mathrm{c}_3 \\ \mathrm{c}_0 - i\mathrm{c}_1 - \mathrm{c}_2 + i\mathrm{c}_3 \\ \mathrm{c}_0 - \mathrm{c}_1 + \mathrm{c}_2 - \mathrm{c}_3 \\ \mathrm{c}_0 + i\mathrm{c}_1 - \mathrm{c}_2 - i\mathrm{c}_3 \end{bmatrix} = \begin{bmatrix} \lambda_0 \\ \lambda_1 \\ \lambda_2 \\ \lambda_3 \end{bmatrix}. \tag{49}$$

The product between the circulant matrix $\mathbf{G}$ and the vector $\mathbf{x} = (\mathrm{x}_0, \mathrm{x}_1, \mathrm{x}_2, \mathrm{x}_3)^T$ can be expressed as follows:

$$\begin{aligned} \mathbf{y} = \mathbf{Gx} &= \mathbf{F}^{-1} \boldsymbol{\Lambda} \, \mathbf{F} \, \mathbf{x} \\ &= \mathbf{F}^{-1} \big(\mathrm{diag}(\boldsymbol{\lambda}) \, (\mathbf{Fx}) \big) \\ &= \mathbf{F}^{-1} \big(\mathrm{diag}(\mathbf{Fc}) \, (\mathbf{Fx}) \big). \end{aligned} \tag{50}$$

The same approach works[28] for an n-by-n matrix $\mathbf{G}$. Thus, the vector $\mathbf{y}$ can be computed efficiently using two FFTs and one IFFT as follows:

$$\mathbf{y} = \mathrm{IFFT}\big(\mathrm{FFT}(\mathbf{c}) \times \mathrm{FFT}(\mathbf{x}) \big), \tag{51}$$

where $\times$ denotes element-by-element multiplication. Algorithm S4 implements this formula. It runs in $O(n \log n)$ time.

A circulant matrix can also be generated by circular shifts of its first row instead of its first column. Thus, there is an alternative formula for $\mathbf{y}$ that diagonalizes $\mathbf{G}$ using its first row $\mathbf{r} = (\mathrm{c}_0, \mathrm{c}_{n-1}, \mathrm{c}_{n-2}, \ldots, \mathrm{c}_1)$, i.e.,

$$\begin{aligned} \mathbf{y} = \mathbf{Gx} &= \mathbf{F}^{-1} \, \boldsymbol{\Lambda} \, \mathbf{F} \, \mathbf{x} \\ &= n \, \mathbf{F}^{-1} \big(\mathrm{diag}(\mathbf{r} \, \mathbf{F}^{-1}) \, (\mathbf{Fx}) \big) \\ &= n \, \mathbf{F}^{-1} \big(\mathrm{diag}(\mathbf{F}^{-1} \mathbf{r}^T) \, (\mathbf{Fx}) \big). \end{aligned} \tag{52}$$

This formula can also be computed in $O(n \log n)$ time using one FFT and two IFFTs as follows:

$$\mathbf{y} = n \ \mathrm{IFFT}\big(\mathrm{IFFT}(\mathbf{r}) \times \mathrm{FFT}(\mathbf{x}) \big). \tag{53}$$

Note that in this case there is an extra multiplication by n for all elements of the result vector $\mathbf{y}$. This is due to the normalization by $1/n$ in the definition of $\mathbf{F}^{-1}$ (see Eq. (45)).

For the sake of completeness, Algorithm S5 and Algorithm S6 give the pseudo-code for the *Fast Fourier Transform* (FFT) and the *Inverse Fast Fourier Transform* (IFFT). Both the FFT and the IFFT run in $O(n \log n)$ time[11,14].

Algorithm S4. Multiply a circulant matrix by a vector.

```
CirculantMultiply(c, x) // runs in O(n log n) time
// Compute the product y = Gx of a circulant matrix G
// and a vector x, where G is generated by its first column
// c = (c[0], c[1], ..., c[n−1]).
n ← Length(c);
Assert(Length(x) = n);
C ← FFT(c);
X ← FFT(x);
Y ← EmptyArray(n);
for k ← 0 to n − 1 do
    Y[k] ← C[k] · X[k];
end for
y ← IFFT(Y);
return y;
```

Algorithm S5. FFT algorithm. Runs in $O(n \log n)$ time.

```
FFT(x)
n ← LENGTH(x);
if n = 1 then
    return x;
end if
x_E ← (x[0], x[2], ..., x[n−2]);        // even
x_O ← (x[1], x[3], ..., x[n−1]);        // odd
y′ ← FFT(x_E);
y″ ← FFT(x_O);
y ← EMPTYARRAY(n);
for k ← 0 to n/2 − 1 do
    w ← e^(−i2πk/n);
    y[k]         ← y′[k] + w · y″[k];
    y[k + (n/2)] ← y′[k] − w · y″[k];
end for
return y;
```

Algorithm S6. IFFT algorithm. Runs in $O(n \log n)$ time.

```
IFFT(y)
n ← LENGTH(y);
if n = 1 then
    return y;
end if
y_E ← (y[0], y[2], ..., y[n−2]);        // even
y_O ← (y[1], y[3], ..., y[n−1]);        // odd
x′ ← IFFT(y_E);
x″ ← IFFT(y_O);
x ← EMPTYARRAY(n);
for k ← 0 to n/2 − 1 do
    w ← e^(i2πk/n);
    x[k]         ← (x′[k] + w · x″[k])/2;
    x[k + (n/2)] ← (x′[k] − w · x″[k])/2;
end for
return x;
```

B. Multiplying a Skew-Circulant Matrix by a Vector

A skew-circulant matrix is a square matrix obtained by negating each element above the main diagonal of a circulant matrix[28]. A skew-circulant matrix is also often called an f-circulant matrix with $f = -1$, e.g., see references 26 and 27.

Let $\mathbf{S}$ be a 4-by-4 skew-circulant matrix that is generated by its first column $\mathbf{c} = (c_0, c_1, c_2, c_3)$, i.e.,

$$\mathbf{S} = \begin{bmatrix} c_0 & -c_3 & -c_2 & -c_1 \\ c_1 & c_0 & -c_3 & -c_2 \\ c_2 & c_1 & c_0 & -c_3 \\ c_3 & c_2 & c_1 & c_0 \end{bmatrix}. \tag{54}$$

The matrix $\mathbf{S}$ can be expressed as the following product[26,28] of three matrices:

$$\mathbf{S} = \mathbf{H}^{-1} \hat{\mathbf{G}}\, \mathbf{H}, \tag{55}$$

where $\hat{\mathbf{G}}$ is a circulant matrix and both $\mathbf{H}$ and $\mathbf{H}^{-1}$ are diagonal matrices.

The matrix $\mathbf{H}$ is defined as follows:

$$\mathbf{H} = \operatorname{diag}(\sigma^0, \sigma^1, \sigma^2, \sigma^3) = \begin{bmatrix} 1 & 0 & 0 & 0 \\ 0 & e^{-\frac{1i\pi}{4}} & 0 & 0 \\ 0 & 0 & e^{-\frac{2i\pi}{4}} & 0 \\ 0 & 0 & 0 & e^{-\frac{3i\pi}{4}} \end{bmatrix}, \tag{56}$$

where $\sigma = e^{-\frac{i\pi}{n}}$. The inverse matrix $\mathbf{H}^{-1}$ is equal to:

$$\mathbf{H}^{-1} = \operatorname{diag}(\sigma^0, \sigma^{-1}, \sigma^{-2}, \sigma^{-3}) = \begin{bmatrix} 1 & 0 & 0 & 0 \\ 0 & e^{\frac{1i\pi}{4}} & 0 & 0 \\ 0 & 0 & e^{\frac{2i\pi}{4}} & 0 \\ 0 & 0 & 0 & e^{\frac{3i\pi}{4}} \end{bmatrix}. \tag{57}$$

The circulant matrix $\hat{\mathbf{G}}$ is generated by its first column $\hat{\mathbf{c}}$, which is equal to the product of $\mathbf{H}$ with the column vector $\mathbf{c}$, i.e., $\hat{\mathbf{c}} = \mathbf{H}\,\mathbf{c}$. Thus, $\hat{\mathbf{G}}$ has the following form:

$$\hat{\mathbf{G}} = \begin{bmatrix} c_0\,\sigma^0 & c_3\,\sigma^3 & c_2\,\sigma^2 & c_1\,\sigma^1 \\ c_1\,\sigma^1 & c_0\,\sigma^0 & c_3\,\sigma^3 & c_2\,\sigma^2 \\ c_2\,\sigma^2 & c_1\,\sigma^1 & c_0\,\sigma^0 & c_3\,\sigma^3 \\ c_3\,\sigma^3 & c_2\,\sigma^2 & c_1\,\sigma^1 & c_0\,\sigma^0 \end{bmatrix}. \tag{58}$$

Combining Eq. (55) with Eq. (50) leads to the following formula for computing the product of the skew-circulant matrix $\mathbf{S}$ with the vector $\mathbf{x} = (x_0, x_1, x_2, x_3)^T$:

$$\begin{aligned} \mathbf{y} = \mathbf{S}\mathbf{x} = \mathbf{H}^{-1}\hat{\mathbf{G}}\,\mathbf{H}\,\mathbf{x} &= \mathbf{H}^{-1}\,\mathbf{F}^{-1}\,\hat{\mathbf{\Lambda}}\,\mathbf{F}\,\mathbf{H}\,\mathbf{x} \\ &= \mathbf{H}^{-1}\big(\mathbf{F}^{-1}\operatorname{diag}(\mathbf{F}\,\mathbf{H}\,\mathbf{c})\,(\mathbf{F}\,\mathbf{H}\,\mathbf{x})\big) \\ &= \mathbf{H}^{-1}\big(\mathbf{F}^{-1}\operatorname{diag}(\mathbf{F}\,\hat{\mathbf{c}})\,(\mathbf{F}\,\hat{\mathbf{x}})\big), \end{aligned} \tag{59}$$

where $\hat{\mathbf{c}} = \mathbf{H}\,\mathbf{c}$ and $\hat{\mathbf{x}} = \mathbf{H}\,\mathbf{x}$.

The same method can be applied if $\mathbf{S}$ is an n-by-n skew-circulant matrix[28]. In this case, Eq. (59) can be computed efficiently using two FFTs and one IFFT, i.e.,

$$\mathbf{y} = \mathbf{H}^{-1}\big(\operatorname{IFFT}\big(\operatorname{FFT}(\hat{\mathbf{c}}) \times \operatorname{FFT}(\hat{\mathbf{x}})\big)\big), \tag{60}$$

where $\times$ denotes elementwise multiplication. Algorithm S7 implements this approach. It runs in $O(n \log n)$ time.

Because $\mathbf{H}$ and $\mathbf{H}^{-1}$ are diagonal matrices, multiplying each of them by a vector reduces to scaling the elements of the vector, which can be done in $O(n)$ time. This scaling does not affect the computational complexity of Algorithm S7.

Algorithm S7. Multiply a skew-circulant matrix by a vector.

```
SKEWCIRCULANTMULTIPLY(c, x) // in O(n log n) time
n ← LENGTH(c);
ASSERT(LENGTH(x) = n);
ĉ ← EMPTYARRAY(n);
x̂ ← EMPTYARRAY(n);
for k ← 0 to n − 1 do
    ĉ[k] ← c[k] · e^(−ikπ/n);        // ĉ = H c
    x̂[k] ← x[k] · e^(−ikπ/n);        // x̂ = H x
end for
y ← CIRCULANTMULTIPLY(ĉ, x̂);   // call Algorithm S4
for k ← 0 to n − 1 do
    y[k] ← y[k] · e^(ikπ/n);         // multiply by H^−1
end for
return y;
```

Supplementary Appendix E: Expressing the Inverse Matrix $\hat{\boldsymbol{W}}^{-1}$

This appendix shows how to express the inverse matrix $\hat{\boldsymbol{W}}^{-1}$ using structured matrices so that any product between this matrix and a vector can be computed in $O(n \log n)$ time.

Let $\mathbf{T}$ be an n-by-n Toeplitz matrix generated by the vector $\mathbf{r} = (\mathrm{r}_0, \mathrm{r}_1, \mathrm{r}_2, \dots, \mathrm{r}_{n-1})$ that specifies its first row and by the vector $\mathbf{c} = (\mathrm{c}_0, \mathrm{c}_1, \mathrm{c}_2, \dots, \mathrm{c}_{n-1})$ that specifies its first column, where it is assumed that $\mathrm{r}_0 = \mathrm{c}_0$. The inverse matrix $\mathbf{T}^{-1}$ may not be Toeplitz. Nevertheless, the Gohberg–Semencul formula[19,20] makes it possible to express $\mathbf{T}^{-1}$ using upper-triangular and lower-triangular Toeplitz matrices.

In other words, the formula states that there exist a vector $\mathbf{u} = (\mathrm{u}_0, \mathrm{u}_1, \mathrm{u}_2, \dots, \mathrm{u}_{n-1})$ such that $\mathrm{u}_0 \neq 0$ and a vector $\mathbf{v} = (\mathrm{v}_0, \mathrm{v}_1, \mathrm{v}_2, \dots, \mathrm{v}_{n-1})$ that satisfy the following matrix equation:

$$
\mathrm{u}_0\mathbf{T}^{-1} = \underbrace{\begin{bmatrix} \mathrm{u}_0 & 0 & 0 & \dots & 0 & 0 \\ \mathrm{u}_1 & \mathrm{u}_0 & 0 & \dots & 0 & 0 \\ \mathrm{u}_2 & \mathrm{u}_1 & \mathrm{u}_0 & \dots & 0 & 0 \\ \vdots & \vdots & \vdots & \ddots & \vdots & \vdots \\ \mathrm{u}_{n-2} & \mathrm{u}_{n-3} & \mathrm{u}_{n-4} & \dots & \mathrm{u}_0 & 0 \\ \mathrm{u}_{n-1} & \mathrm{u}_{n-2} & \mathrm{u}_{n-3} & \dots & \mathrm{u}_1 & \mathrm{u}_0 \end{bmatrix}}_{\boldsymbol{\mathcal{A}}} \underbrace{\begin{bmatrix} \mathrm{v}_{n-1} & \mathrm{v}_{n-2} & \mathrm{v}_{n-3} & \dots & \mathrm{v}_1 & \mathrm{v}_0 \\ 0 & \mathrm{v}_{n-1} & \mathrm{v}_{n-2} & \dots & \mathrm{v}_2 & \mathrm{v}_1 \\ 0 & 0 & \mathrm{v}_{n-1} & \dots & \mathrm{v}_3 & \mathrm{v}_2 \\ \vdots & \vdots & \vdots & \ddots & \vdots & \vdots \\ 0 & 0 & 0 & \dots & \mathrm{v}_{n-1} & \mathrm{v}_{n-2} \\ 0 & 0 & 0 & \dots & 0 & \mathrm{v}_{n-1} \end{bmatrix}}_{\boldsymbol{\mathcal{C}}}
$$
$$
- \underbrace{\begin{bmatrix} 0 & 0 & 0 & \dots & 0 & 0 \\ \mathrm{v}_0 & 0 & 0 & \dots & 0 & 0 \\ \mathrm{v}_1 & \mathrm{v}_0 & 0 & \dots & 0 & 0 \\ \vdots & \vdots & \vdots & \ddots & \vdots & \vdots \\ \mathrm{v}_{n-3} & \mathrm{v}_{n-4} & \mathrm{v}_{n-5} & \dots & 0 & 0 \\ \mathrm{v}_{n-2} & \mathrm{v}_{n-3} & \mathrm{v}_{n-4} & \dots & \mathrm{v}_0 & 0 \end{bmatrix}}_{\boldsymbol{\mathcal{B}}} \underbrace{\begin{bmatrix} 0 & \mathrm{u}_{n-1} & \mathrm{u}_{n-2} & \dots & \mathrm{u}_2 & \mathrm{u}_1 \\ 0 & 0 & \mathrm{u}_{n-1} & \dots & \mathrm{u}_3 & \mathrm{u}_2 \\ 0 & 0 & 0 & \dots & \mathrm{u}_4 & \mathrm{u}_3 \\ \vdots & \vdots & \vdots & \ddots & \vdots & \vdots \\ 0 & 0 & 0 & \dots & 0 & \mathrm{u}_{n-1} \\ 0 & 0 & 0 & \dots & 0 & 0 \end{bmatrix}}_{\boldsymbol{\mathcal{D}}}
$$
$$
= \boldsymbol{\mathcal{A}}\boldsymbol{\mathcal{C}} - \boldsymbol{\mathcal{B}}\boldsymbol{\mathcal{D}}. \quad (61)
$$

The Gohberg–Semencul formula uses two vectors $\mathbf{u}$ and $\mathbf{v}$ to describe the inverse of a square Toeplitz matrix. As proven below, however, the vector $\mathbf{u}$ is not unique in that formula and can be scaled by a non-zero constant without affecting the inverse matrix. In turn, this result is used to prove that if the Toeplitz matrix is symmetric, then its inverse can be generated using only one specifically scaled vector $\mathbf{u}$. Furthermore, this vector is equal to the first column of the inverse matrix.

Theorem 1. ***(The vector*** $\mathbf{u}$ ***is not unique.)*** *Let* $\mathbf{T}$ *be a non-singular n-by-n Toeplitz matrix. Let* $\mathbf{u} = \big(\mathrm{u}_0, \mathrm{u}_1, \dots, \mathrm{u}_{n-1}\big)$ *and* $\mathbf{v} = \big(\mathrm{v}_0, \mathrm{v}_1, \dots, \mathrm{v}_{n-1}\big)$ *be two vectors that determine the inverse matrix* $\mathbf{T}^{-1}$ *in the Gohberg–Semencul formula, i.e.,*

$$\mathrm{u}_0\mathbf{T}^{-1} = \boldsymbol{\mathcal{A}}\boldsymbol{\mathcal{C}} - \boldsymbol{\mathcal{B}}\boldsymbol{\mathcal{D}}, \quad (62)$$

where $\boldsymbol{\mathcal{A}}$, $\boldsymbol{\mathcal{B}}$, $\boldsymbol{\mathcal{C}}$, *and* $\boldsymbol{\mathcal{D}}$ *are specified in* Eq. (61).

Then, the Gohberg–Semencul formula also holds for all vectors $\hat{\mathbf{u}}$ *obtained by multiplying the vector* $\mathbf{u}$ *with a non-zero scaling coefficient. More formally, for each* $\alpha \neq 0$, *the following equation holds:*

$$\hat{\mathrm{u}}_0\mathbf{T}^{-1} = \hat{\boldsymbol{\mathcal{A}}}\boldsymbol{\mathcal{C}} - \boldsymbol{\mathcal{B}}\hat{\boldsymbol{\mathcal{D}}}, \quad (63)$$

where $\hat{\boldsymbol{\mathcal{A}}} = \alpha\boldsymbol{\mathcal{A}}$, $\hat{\boldsymbol{\mathcal{D}}} = \alpha\boldsymbol{\mathcal{D}}$, *and*

$$\hat{\mathbf{u}} = (\hat{\mathrm{u}}_0, \hat{\mathrm{u}}_1, \dots, \hat{\mathrm{u}}_{n-1}) = (\alpha\mathrm{u}_0, \alpha\mathrm{u}_1, \dots, \alpha\mathrm{u}_{n-1}) = \alpha\mathbf{u}. \quad (64)$$

Proof. Because $\alpha \neq 0$, it can be factored out and canceled from both sides of Eq. (63), which leads to Eq. (62). That is,

$$\underbrace{\cancel{\alpha}\mathrm{u}_0}_{\hat{\mathrm{u}}_0}\mathbf{T}^{-1} = \underbrace{(\cancel{\alpha}\boldsymbol{\mathcal{A}})}_{\hat{\boldsymbol{\mathcal{A}}}}\boldsymbol{\mathcal{C}} - \boldsymbol{\mathcal{B}}\underbrace{(\cancel{\alpha}\boldsymbol{\mathcal{D}})}_{\hat{\boldsymbol{\mathcal{D}}}} = \cancel{\alpha}(\boldsymbol{\mathcal{A}}\boldsymbol{\mathcal{C}} - \boldsymbol{\mathcal{B}}\boldsymbol{\mathcal{D}}). \quad (65)$$

□

Theorem 2. *Let* $\mathbf{T}$ *be a non-singular Toeplitz matrix and let* $\mathbf{T}^{-1}$ *be its inverse that is generated by the vectors* $\mathbf{u}$ *and* $\mathbf{v}$ *in the Gohberg–Semencul formula. Then, the first row of* $\mathbf{T}^{-1}$ *is equal to the reverse of the vector* $\mathbf{v}$.

Proof. Let $\mathbf{a} = (\mathrm{u}_0, 0, \dots, 0)$ be the first row of $\boldsymbol{\mathcal{A}}$ and let $\mathbf{b} = (0, 0, \dots, 0)$ be the first row of $\boldsymbol{\mathcal{B}}$ in Eq. (61). Then, the first row of the inverse matrix $\mathbf{T}^{-1}$ can be expressed as follows:

$$\frac{1}{\mathrm{u}_0}\big(\mathbf{a}\boldsymbol{\mathcal{C}} - \underbrace{\mathbf{b}\boldsymbol{\mathcal{D}}}_{\mathbf{0}}\big) = \frac{1}{\cancel{\mathrm{u}_0}}\big(\cancel{\mathrm{u}_0}\,\mathrm{v}_{n-1}, \cancel{\mathrm{u}_0}\,\mathrm{v}_{n-2}, \dots, \cancel{\mathrm{u}_0}\,\mathrm{v}_0\big). \quad (66)$$

□

Theorem 3. *Let* $\mathbf{T}^{-1}$ *be the inverse of an n-by-n Toeplitz matrix* $\mathbf{T}$. *Then, the first column of* $\mathbf{T}^{-1}$ *can be expressed as the product of the vector* $\mathbf{u}$ *with the scalar* ${}^{\mathrm{v}_{n-1}}\!/\!_{\mathrm{u}_0}$.

Proof. Let $\mathbf{c} = \big(\mathrm{v}_{n-1}, 0, \dots, 0\big)^T$ be the first column of $\boldsymbol{\mathcal{C}}$ and let $\mathbf{d} = \big(0, 0, \dots, 0\big)^T$ be the first column of $\boldsymbol{\mathcal{D}}$ in Eq. (61). Then, the first column of $\mathbf{T}^{-1}$ is equal to:

$$\frac{1}{\mathrm{u}_0}\big(\boldsymbol{\mathcal{A}}\mathbf{c} - \underbrace{\boldsymbol{\mathcal{B}}\mathbf{d}}_{\mathbf{0}}\big) = \frac{\mathrm{v}_{n-1}}{\mathrm{u}_0}\mathbf{u}. \quad (67)$$

□

Theorem 4. *Let* $\hat{\boldsymbol{W}}$ *be a symmetric non-singular Toeplitz matrix. Let* $\mathbf{u}$ *and* $\mathbf{v}$ *be two vectors that generate the inverse matrix* $\hat{\boldsymbol{W}}^{-1}$ *using the Gohberg–Semencul formula such that* $\mathrm{u}_0 = \mathrm{v}_{n-1}$. *Then, the vector* $\mathbf{u}$ *is equal to the reverse of the vector* $\mathbf{v}$, *i.e.,*

$$\mathbf{u} = (\mathrm{u}_0, \mathrm{u}_1, \dots, \mathrm{u}_{n-1}) = (\mathrm{v}_{n-1}, \mathrm{v}_{n-2}, \dots, \mathrm{v}_0). \quad (68)$$

Proof. Because the matrix $\hat{\boldsymbol{W}}$ is symmetric, its inverse is also symmetric. This implies that the first column of $\hat{\boldsymbol{W}}^{-1}$ is equal to its first row. Combining the condition that $\mathrm{u}_0 = \mathrm{v}_{n-1}$ with Theorem 2 and Theorem 3 shows that Eq. (68) holds. More formally,

$$\underbrace{\frac{\mathrm{v}_{n-1}}{\mathrm{u}_0}}_{1}\mathbf{u} = \big(\mathrm{v}_{n-1}, \mathrm{v}_{n-2}, \dots, \mathrm{v}_0\big)^T. \quad (69)$$

□

Theorem 5. *Let* $\hat{\boldsymbol{W}}$ *be a symmetric non-singular Toeplitz matrix. Then, there is a vector* $\mathbf{u} = (\mathrm{u}_0, \mathrm{u}_1, \dots, \mathrm{u}_{n-1})$ *in which* $\mathrm{u}_0 \neq 0$ *such that* $\hat{\boldsymbol{W}}^{-1}$ *can be expressed as follows:*

$$\hat{\boldsymbol{W}}^{-1} = \frac{1}{\mathrm{u}_0}\Big(\boldsymbol{\mathcal{A}}\boldsymbol{\mathcal{A}}^T - \boldsymbol{\mathcal{D}}^T\boldsymbol{\mathcal{D}}\Big). \quad (70)$$

Moreover, the vector $\mathbf{u}$ *is equal to the first column of the inverse matrix* $\hat{\boldsymbol{W}}^{-1}$.

Proof. Theorem 1 implies that $\mathbf{u}$ can be scaled by a factor α. In particular, if we set $\alpha = {}^{\mathrm{v}_{n-1}}\!/\!_{\mathrm{u}_0}$, then the value of u_0 after scaling is equal to v_{n-1}. Then, Theorem 4 implies that the scaled vector $\mathbf{u}$ is equal to the reverse of the vector $\mathbf{v}$. Plugging these values into Eq. (61) leads to Eq. (70). Since $\mathrm{u}_0 = \mathrm{v}_{n-1}$, Theorem 3 implies that $\mathbf{u}$ is the first column of $\hat{\boldsymbol{W}}^{-1}$. □

Supplementary Appendix F: Expressing the Generating Vector $\mathbf{u}$

A general formula for the elements of the vector $\mathbf{u}$ can be derived from special cases for the first several values of n and then proven by induction for all n. Because $\mathbf{u}$ determines $\hat{\boldsymbol{W}}^{-1}$, this is sufficient to formulate the ICZT algorithm using structured matrix multiplication.

A. Special Cases of the Formula for the Generating Vector $\mathbf{u}$

1) Special Case for $n = 1$: In this degenerate case both $\hat{\boldsymbol{W}}$ and $\hat{\boldsymbol{W}}^{-1}$ are equal to $[\,1\,]$. Also, $\mathbf{u} = (\mathrm{u}_0) = (1)$.

2) Special Case for $n = 2$: Suppose that $n = 2$. Then, $\hat{\boldsymbol{W}}$ is a 2-by-2 matrix that is defined by Eq. (9). That is,

$$\hat{\boldsymbol{W}} = \begin{bmatrix} W^{-\frac{(0-0)^2}{2}} & W^{-\frac{(0-1)^2}{2}} \\ W^{-\frac{(1-0)^2}{2}} & W^{-\frac{(1-1)^2}{2}} \end{bmatrix} = \begin{bmatrix} 1 & W^{-\frac{1}{2}} \\ W^{-\frac{1}{2}} & 1 \end{bmatrix}, \quad (71)$$

which is well-defined for each $W \in \mathbb{C} \setminus \{0\}$.

By definition, the product of a matrix with its inverse is equal to the identity matrix, i.e., $\hat{\boldsymbol{W}}\,\hat{\boldsymbol{W}}^{-1} = \mathbf{I}$. Theorem 5 implies that the first column of $\hat{\boldsymbol{W}}^{-1}$ is equal to a two-element vector $\mathbf{u} = (\mathrm{u}_0, \mathrm{u}_1)^T$. Therefore, the elements of $\mathbf{u}$ can be expressed as functions of the transform parameter W by solving the following linear system:

$$\underbrace{\begin{bmatrix} 1 & W^{-\frac{1}{2}} \\ W^{-\frac{1}{2}} & 1 \end{bmatrix}}_{\hat{\boldsymbol{W}}} \underbrace{\begin{bmatrix} \mathrm{u}_0 \\ \mathrm{u}_1 \end{bmatrix}}_{\mathbf{u}} = \begin{bmatrix} 1 \\ 0 \end{bmatrix}. \quad (72)$$

This leads to the following formulas for u_0 and u_1:

$$\mathrm{u}_0 = \frac{W}{W-1}, \qquad \mathrm{u}_1 = -\frac{W^{\frac{1}{2}}}{W-1}. \quad (73)$$

To summarize, the column vector $\mathbf{u}$, which is equal to the first column of the inverse matrix $\hat{\boldsymbol{W}}^{-1}$, can be expressed as:

$$\mathbf{u} = \left[\frac{W}{W-1}, -\frac{W^{\frac{1}{2}}}{W-1}\right]^T. \quad (74)$$

Plugging $\mathbf{u}$ into Eq. (70) leads to the correct value for $\hat{\boldsymbol{W}}^{-1}$:

$$\begin{aligned} \hat{\boldsymbol{W}}^{-1} &= \frac{1}{\mathrm{u}_0}\left(\begin{bmatrix} \mathrm{u}_0 & 0 \\ \mathrm{u}_1 & \mathrm{u}_0 \end{bmatrix}\begin{bmatrix} \mathrm{u}_0 & \mathrm{u}_1 \\ 0 & \mathrm{u}_0 \end{bmatrix} - \begin{bmatrix} 0 & 0 \\ \mathrm{u}_1 & 0 \end{bmatrix}\begin{bmatrix} 0 & \mathrm{u}_1 \\ 0 & 0 \end{bmatrix}\right) \\ &= \frac{1}{\mathrm{u}_0}\left(\begin{bmatrix} \mathrm{u}_0\,\mathrm{u}_0 & \mathrm{u}_0\mathrm{u}_1 \\ \mathrm{u}_1\,\mathrm{u}_0 & \cancel{\mathrm{u}_1\,\mathrm{u}_1} + \mathrm{u}_0\,\mathrm{u}_0 \end{bmatrix} - \begin{bmatrix} 0 & 0 \\ 0 & \cancel{\mathrm{u}_1\,\mathrm{u}_1} \end{bmatrix}\right) \\ &= \begin{bmatrix} \frac{W}{W-1} & -\frac{W^{\frac{1}{2}}}{W-1} \\ -\frac{W^{\frac{1}{2}}}{W-1} & \frac{W}{W-1} \end{bmatrix}, \qquad W \notin \{0, 1\}. \quad (75) \end{aligned}$$

3) Special Case for $n = 3$: In this case, $\hat{\boldsymbol{W}}$ is a 3-by-3 Toeplitz matrix. From Eq. (9) we know that each element of $\hat{\boldsymbol{W}}$ is a function of the transform parameter W. More formally,

$$\hat{\boldsymbol{W}} = \begin{bmatrix} W^{-\frac{(0-0)^2}{2}} & W^{-\frac{(0-1)^2}{2}} & W^{-\frac{(0-2)^2}{2}} \\ W^{-\frac{(1-0)^2}{2}} & W^{-\frac{(1-1)^2}{2}} & W^{-\frac{(1-2)^2}{2}} \\ W^{-\frac{(2-0)^2}{2}} & W^{-\frac{(2-1)^2}{2}} & W^{-\frac{(2-2)^2}{2}} \end{bmatrix} = \begin{bmatrix} 1 & W^{-\frac{1}{2}} & W^{-2} \\ W^{-\frac{1}{2}} & 1 & W^{-\frac{1}{2}} \\ W^{-2} & W^{-\frac{1}{2}} & 1 \end{bmatrix}. \quad (76)$$

Similarly to the 2-by-2 case, the vector $\mathbf{u}$ is a solution to the following linear system:

$$\underbrace{\begin{bmatrix} 1 & W^{-\frac{1}{2}} & W^{-2} \\ W^{-\frac{1}{2}} & 1 & W^{-\frac{1}{2}} \\ W^{-2} & W^{-\frac{1}{2}} & 1 \end{bmatrix}}_{\hat{\boldsymbol{W}}} \underbrace{\begin{bmatrix} \mathrm{u}_0 \\ \mathrm{u}_1 \\ \mathrm{u}_2 \end{bmatrix}}_{\mathbf{u}} = \begin{bmatrix} 1 \\ 0 \\ 0 \end{bmatrix}. \quad (77)$$

In this case, the elements of $\mathbf{u}$ can be expressed in terms of the transform parameter W as shown below:

$$\mathbf{u} = \begin{bmatrix} \frac{W^3}{(W-1)(W^2-1)} \\ -\frac{W^{\frac{3}{2}}}{(W-1)(W-1)} \\ \frac{W^2}{(W-1)(W^2-1)} \end{bmatrix}. \quad (78)$$

Plugging this vector into Eq. (70) leads to the inverse matrix $\hat{\boldsymbol{W}}^{-1}$ (see Appendix A). In contrast to the 2-by-2 case, the 3-by-3 inverse matrix is no longer Toeplitz because the elements along its main diagonal are not identical, i.e.,

$$\hat{\boldsymbol{W}}^{-1} = \begin{bmatrix} \frac{W^3}{(W-1)(W^2-1)} & -\frac{W^{\frac{3}{2}}}{(W-1)^2} & \frac{W^2}{(W-1)(W^2-1)} \\ -\frac{W^{\frac{3}{2}}}{(W-1)^2} & \frac{W^2+1}{(W-1)^2} & -\frac{W^{\frac{3}{2}}}{(W-1)^2} \\ \frac{W^2}{(W-1)(W^2-1)} & -\frac{W^{\frac{3}{2}}}{(W-1)^2} & \frac{W^3}{(W-1)(W^2-1)} \end{bmatrix}. \quad (79)$$

Repeating this process for other values of n allowed us to derive a general formula for the elements of $\mathbf{u}$. The next two sections state and prove this formula.

B. General Formula for the Generating Vector $\mathbf{u}$

In the general case, for each $k \in \{0, 1, 2, \ldots, n-1\}$, the elements of the vector $\mathbf{u}$ can be computed using the following formula:

$$\mathrm{u}_k = \left(\hat{\boldsymbol{W}}^{-1}\right)_{k+1,1} = (-1)^k \frac{W^{\frac{2k^2-(2n-1)k+n(n-1)}{2}}}{\prod_{s=1}^{n-k-1}(W^s-1)\prod_{s=1}^{k}(W^s-1)}, \quad (80)$$

which is proven in the next section. For each k, the value of u_k can be computed in $O(1)$ using precomputed values of the products of polynomials that appear in the denominator of Eq. (80). These values can be computed in a separate loop that runs in $O(n)$.

The vector $\mathbf{u}$ can then be plugged into Eq. (70) to compute the inverse matrix $\hat{\boldsymbol{W}}^{-1}$. It can also be used as an input to $O(n \log n)$ algorithms that compute the product of $\hat{\boldsymbol{W}}^{-1}$ with a vector, without actually storing $\hat{\boldsymbol{W}}^{-1}$ in memory. This approach is used by the $O(n \log n)$ ICZT algorithm that is shown in Algorithm 2.

C. Proof of the General Formula for the Generating Vector $\mathbf{u}$

This section shows that for each $n \in \mathbb{N}$ the vector $\mathbf{u}$, which is defined by Eq. (80), is indeed the generating vector for the inverse matrix $\hat{\boldsymbol{W}}^{-1}$. Theorem 5 implies that this can be done by showing that $\mathbf{u}$ is the first column of $\hat{\boldsymbol{W}}^{-1}$.

In the proofs it is necessary to distinguish between the vector $\mathbf{u} = (\mathrm{u}_0, \mathrm{u}_1, \ldots, \mathrm{u}_{n-1})$ of length n and the vector $\mathbf{u} = (\mathrm{u}_0, \mathrm{u}_1, \ldots, \mathrm{u}_{n-1}, \mathrm{u}_n)$ of length $n+1$. Thus, the number of dimensions will be indicated with a left superscript for both $\mathbf{u}$ and its elements. Using this notation, the k-th element of ${}^n\mathbf{u} = \{{}^n\mathrm{u}_0, {}^n\mathrm{u}_1, \ldots, {}^n\mathrm{u}_{n-1}\}$ is given by:

$$ {}^n\mathrm{u}_k = (-1)^k \frac{W^{\frac{2k^2-(2n-1)k+n(n-1)}{2}}}{\prod_{s=1}^{n-k-1}(W^s-1)\prod_{s=1}^{k}(W^s-1)}. \tag{81} $$

The following lemma expresses ${}^{n+1}\mathrm{u}_k$ in terms of ${}^n\mathrm{u}_k$.

Lemma 6. *Let $n \in \mathbb{N}$ and let $k \in \{0, 1, 2, \ldots, n-1\}$. Then, ${}^{n+1}\mathrm{u}_k$ can be derived from ${}^n\mathrm{u}_k$ as follows:*

$$ {}^{n+1}\mathrm{u}_k = {}^n\mathrm{u}_k \, \frac{W^{n-k}}{W^{n-k}-1}. \tag{82} $$

Proof. Replacing n with $n+1$ in Eq. (81) leads to:

$$ {}^{n+1}\mathrm{u}_k = (-1)^k \frac{W^{\frac{2k^2-(2(n+1)-1)k+(n+1)(n+1-1)}{2}}}{\prod_{s=1}^{(n+1)-k-1}(W^s-1)\prod_{s=1}^{k}(W^s-1)}. \tag{83} $$

Let $p/2$ and $q/2$ be the powers of W in the numerators of Eqs. (81) and (83), respectively. Then, q is equal to:

$$ \begin{aligned} q &= 2k^2 - \big(2(n+1)-1\big)k + (n+1)(n+1-1) \\ &= 2k^2 - (2n+2-1)k + (n-1+2)n \\ &= \underbrace{2k^2 - (2n-1)k + n(n-1)}_{p} + 2(n-k). \end{aligned} \tag{84} $$

Therefore,

$$ W^{\frac{q}{2}} = W^{\frac{p}{2}} W^{n-k}. \tag{85} $$

Eqs. (81) and (83) have similar denominators except that the first product in Eq. (81) goes from 1 to $n-k-1$, while in Eq. (83) it goes from 1 to $n-k$. Thus, the denominator of Eq. (83) has one extra term, which is equal to $(W^{n-k}-1)$. Combining this with Eq. (85) leads to Eq. (82). ☐

The next lemma expresses ${}^n\mathrm{u}_{k-1}$ in terms of ${}^n\mathrm{u}_k$.

Lemma 7. *Let $n \in \mathbb{N}$ and let $k \in \{1, 2, \ldots, n-1\}$. Then, ${}^n\mathrm{u}_{k-1}$ can be expressed as follows:*

$$ {}^n\mathrm{u}_{k-1} = -{}^n\mathrm{u}_k \, \frac{W^k-1}{W^{n-k}-1} \, W^{n-2k+\frac{1}{2}}. \tag{86} $$

Proof. Replacing k with $k-1$ in Eq. (81) leads to the following:

$$ \mathrm{u}_{k-1} = (-1)^{k-1} \frac{W^{\frac{2(k-1)^2-(2n-1)(k-1)+n(n-1)}{2}}}{\prod_{s=1}^{n-(k-1)-1}(W^s-1)\prod_{s=1}^{k-1}(W^s-1)}. \tag{87} $$

Let $p/2$ be the power of W in the numerator of Eq. (81) and $q/2$ be the power of W in the numerator of Eq. (87). Then, q can be expressed in terms of p using the following formula:

$$ \begin{aligned} q &= 2(k-1)^2 - (2n-1)(k-1) + n(n-1) \\ &= 2k^2 - 4k + 2 - (2n-1)k + 2n - 1 + n(n-1) \\ &= \underbrace{2k^2 - (2n-1)k + n(n-1)}_{p} + 2n - 4k + 1. \end{aligned} \tag{88} $$

Therefore,

$$ W^{\frac{q}{2}} = W^{\frac{p}{2}} \, W^{n-2k+\frac{1}{2}}. \tag{89} $$

The first product in the denominator of Eq. (87) is equal to:

$$ \prod_{s=1}^{n-(k-1)-1}(W^s-1) = (W^{n-k}-1)\prod_{s=1}^{n-k-1}(W^s-1). \tag{90} $$

In this form, it is almost the same as the corresponding product in Eq. (81), except for the extra term $(W^{n-k}-1)$. The second product in the denominator of Eq. (87) is equal to:

$$ \prod_{s=1}^{k-1}(W^s-1) = \frac{1}{(W^k-1)}\prod_{s=1}^{k}(W^s-1), \tag{91} $$

which has one more term, i.e., $1/(W^k-1)$, than the corresponding product in Eq. (81).

Combining Eqs. (89), (90), and (91) leads to Eq. (86). ☐

The following lemma states a recursive formula for ${}^{n+1}\mathrm{u}_k$, which is expressed in terms of ${}^n\mathrm{u}_k$ and ${}^n\mathrm{u}_{k-1}$.

Lemma 8. *For each $n \in \mathbb{N}$ and each $k \in \{1, 2, \ldots, n-1\}$, the value of ${}^{n+1}\mathrm{u}_k$ can be expressed as follows:*

$$ {}^{n+1}\mathrm{u}_k = \frac{{}^n\mathrm{u}_k \, W^n \; - \; {}^n\mathrm{u}_{k-1} \, W^{k-\frac{1}{2}}}{W^n-1}. \tag{92} $$

Proof. Using Lemma 6, the left-hand side of Eq. (92) can be expressed in terms of ${}^n\mathrm{u}_k$ as follows:

$$ {}^{n+1}\mathrm{u}_k = {}^n\mathrm{u}_k \, \frac{W^{n-k}}{W^{n-k}-1}. \tag{93} $$

Using Lemma 7, which expresses ${}^n\mathrm{u}_{k-1}$ in terms of ${}^n\mathrm{u}_k$, the right-hand side of Eq. (92) can be stated as shown below:

$$ \frac{{}^n\mathrm{u}_k W^n - (-{}^n\mathrm{u}_k)\,\dfrac{W^k-1}{W^{n-k}-1}\,W^{n-2k+\frac{1}{2}}W^{k-\frac{1}{2}}}{W^n-1}. \tag{94} $$

The rest of the proof shows that Eq. (94) is equal to Eq. (93).

The expression in Eq. (94) can be simplified as follows:

$$ {}^n\mathrm{u}_k \left[\frac{W^n(W^{n-k}-1) + (W^k-1)W^{n-k}}{(W^{n-k}-1)(W^n-1)}\right]. \tag{95} $$

Expanding the numerator leads to:

$$ {}^n\mathrm{u}_k \, \frac{W^nW^{n-k} - \cancel{W^n} + \cancel{W^n} - W^{n-k}}{(W^{n-k}-1)(W^n-1)}. \tag{96} $$

Finally, we get

$$ {}^n\mathrm{u}_k \, \frac{W^{n-k}\cancel{(W^n-1)}}{(W^{n-k}-1)\cancel{(W^n-1)}}, \tag{97} $$

which completes the proof. ☐

Let ${}^{n}P(x)$ be the following polynomial:

$$ {}^{n}P(x) = \sum_{k=0}^{n-1} {}^{n}p_k\, x^k = \sum_{k=0}^{n-1} {}^{n}\mathrm{u}_k\, W^{-\frac{k^2}{2}} x^k, \tag{98} $$

where the k-th coefficient ${}^{n}p_k$ is obtained by multiplying the k-th element of the vector ${}^{n}\mathbf{u}$ by $W^{-\frac{k^2}{2}}$. In other words,

$$ {}^{n}p_k = {}^{n}\mathrm{u}_k\, W^{-\frac{k^2}{2}}. \tag{99} $$

Let ${}^{n}F(x)$ be another polynomial that is defined as follows:

$$ {}^{n}F(x) = \sum_{j=0}^{n-1} y_j\, f_j(x), \tag{100} $$

where

$$ f_j(x) = \prod_{\substack{0\le k<n\\ k\ne j}} \frac{(x - x_k)}{(x_j - x_k)}. \tag{101} $$

That is, ${}^{n}F(x)$ is the Lagrange polynomial[30,31] that interpolates the n points $(x_0, y_0), (x_1, y_1), \ldots, (x_{n-1}, y_{n-1})$. In particular, if $x_0 = W^0, x_1 = W^1, \ldots, x_{n-1} = W^{n-1}$, then Eq. (101) becomes:

$$ f_j(x) = \prod_{\substack{0\le k<n\\ k\ne j}} \frac{(x - W^k)}{(W^j - W^k)}. \tag{102} $$

Furthermore, if $y_0 = 1$ and $y_1 = y_2 = \cdots = y_{n-1} = 0$, then only $f_0(x)$ contributes to ${}^{n}F(x)$ and Eq. (100) simplifies to only one product that will be denoted with ${}^{n}L(x)$, i.e.,

$$ {}^{n}L(x) = \prod_{k=1}^{n-1} \frac{(x - W^k)}{(1 - W^k)}. \tag{103} $$

For $n{+}1$, the formula for ${}^{n+1}P(x)$ uses the elements of ${}^{n+1}\mathbf{u}$ instead of the elements of ${}^{n}\mathbf{u}$. Also, the formula for ${}^{n+1}L(x)$ includes one more fractional term. More formally,

$$ {}^{n+1}P(x) = \sum_{k=0}^{n} {}^{n+1}p_k\, x^k = \sum_{k=0}^{n} {}^{n+1}\mathrm{u}_k\, W^{-\frac{k^2}{2}} x^k, \tag{104} $$

$$ {}^{n+1}L(x) = \prod_{k=1}^{n} \frac{(x - W^k)}{(1 - W^k)} = {}^{n}L(x)\, \frac{(x - W^n)}{(1 - W^n)}. \tag{105} $$

We will use mathematical induction to show that ${}^{n}P(x) = {}^{n}L(x)$ for each $n \in \mathbb{N}$. The following lemma proves that ${}^{1}P(x) = {}^{1}L(x)$, which is the base case of the induction.

Lemma 9. *Both ${}^{1}P(x)$ and ${}^{1}L(x)$ are equal to 1, i.e.,*

$$ {}^{1}P(x) = {}^{1}L(x) = 1. \tag{106} $$

Proof. The product that defines ${}^{n}L(x)$ in Eq. (103) becomes degenerate when $n = 1$ and evaluates to 1, i.e., ${}^{1}L(x) = 1$. Similarly, for $n = 1$, Eq. (98) simplifies to:

$$ {}^{1}P(x) = \sum_{k=0}^{1-1} {}^{1}\mathrm{u}_k\, W^{-\frac{k^2}{2}} x^k = {}^{1}\mathrm{u}_0\, W^{-\frac{0^2}{2}} x^0 = {}^{1}\mathrm{u}_0. \tag{107} $$

This formula evaluates to 1, which follows from Eq. (81), i.e.,

$$ {}^{1}P(x) = (-1)^0 \frac{W^{\frac{0-0+0}{2}}}{\prod_{s=1}^{1-0-1}(W^s - 1)\prod_{s=1}^{0}(W^s - 1)} = 1. \tag{108} $$

□

The following lemma proves an inductive formula that expresses the coefficients of the Lagrange polynomial ${}^{n+1}L(x)$ in terms of the coefficients of ${}^{n}L(x)$.

Lemma 10. *Let ${}^{n}\ell_k$ be the k-th coefficient of the Lagrange polynomial ${}^{n}L(x)$ that is defined in* Eq. (103)*, i.e.,*

$$ {}^{n}L(x) = \sum_{k=0}^{n-1} {}^{n}\ell_k\, x^k = \prod_{k=1}^{n-1} \frac{(x - W^k)}{(1 - W^k)}. \tag{109} $$

Then, for each $n \in \mathbb{N}$ and each $k \in \{0, 1, 2, \ldots, n\}$, the value of ${}^{n+1}\ell_k$ in ${}^{n+1}L(n)$ is given by the following formula:

$$ {}^{n+1}\ell_k = \begin{cases} \dfrac{{}^{n}\ell_0\, W^n}{W^n - 1}, & \text{if } k = 0,\\[2mm] \dfrac{{}^{n}\ell_k\, W^n - {}^{n}\ell_{k-1}}{W^n - 1}, & \text{if } k \in \{1, 2, \ldots, n{-}1\},\\[2mm] -\dfrac{{}^{n}\ell_{n-1}}{W^n - 1}, & \text{if } k = n. \end{cases} \tag{110} $$

Proof. Expanding Eq. (105) in terms of ${}^{n}\ell_k$ leads to:

$$ \begin{aligned} {}^{n+1}L(x) &= {}^{n}L(x)\, \frac{(x - W^n)}{(1 - W^n)} = \sum_{k=0}^{n-1} {}^{n}\ell_k\; x^k\, \frac{(x - W^n)}{(1 - W^n)} \\ &= -\sum_{k=0}^{n-1} \frac{{}^{n}\ell_k}{W^n - 1}\, x^{k+1} + \sum_{k=0}^{n-1} \frac{{}^{n}\ell_k\, W^n}{W^n - 1}\, x^k. \end{aligned} \tag{111} $$

The terms in Eq. (111) can be grouped by the powers of x. The coefficients of the resulting polynomial are equal to the coefficients ${}^{n+1}\ell_k$ of ${}^{n+1}L(x)$. More formally,

$$ \begin{aligned} {}^{n+1}L(x) &= -\frac{{}^{n}\ell_k}{W^n{-}1}\, x^n - \sum_{k=0}^{n-2} \frac{{}^{n}\ell_k}{W^n{-}1}\, x^{k+1} + \sum_{k=0}^{n-1} \frac{{}^{n}\ell_k W^n}{W^n{-}1} x^k \\ &= -\frac{{}^{n}\ell_{n-1}}{W^n - 1}\, x^n - \sum_{k=1}^{n-1} \frac{{}^{n}\ell_{k-1}}{W^n{-}1}\, x^k \\ &\quad + \sum_{k=1}^{n-1} \frac{{}^{n}\ell_k\, W^n}{W^n - 1}\, x^k + \frac{{}^{n}\ell_0\, W^n}{W^n - 1}\, x^0 \\ &= \underbrace{\frac{-{}^{n}\ell_{n-1}}{W^n{-}1}}_{{}^{n+1}\ell_n} x^n + \sum_{k=1}^{n-1} \underbrace{\frac{{}^{n}\ell_k\, W^n - {}^{n}\ell_{k-1}}{W^n - 1}}_{{}^{n+1}\ell_k} x^k + \underbrace{\frac{{}^{n}\ell_0\, W^n}{W^n{-}1}}_{{}^{n+1}\ell_0} x^0 \\ &= \sum_{k=0}^{n} {}^{n+1}\ell_k\, x^k. \end{aligned} \tag{112} $$

□

The next lemma expresses the coefficients of ${}^{n+1}P(x)$ in terms of the coefficients of ${}^{n}P(x)$.

Lemma 11. *Let $n \in \mathbb{N}$ and $k \in \{0, 1, 2, \ldots, n\}$. Then, each coefficient of the polynomial ${}^{n+1}P(x)$ can be computed using the following formula:*

$$ {}^{n+1}p_k = \begin{cases} \dfrac{{}^{n}p_0\, W^n}{W^n - 1}, & \text{if } k = 0,\\[2mm] \dfrac{{}^{n}p_k\, W^n - {}^{n}p_{k-1}}{W^n - 1}, & \text{if } k \in \{1, 2, \ldots, n{-}1\},\\[2mm] -\dfrac{{}^{n}p_{n-1}}{W^n - 1}, & \text{if } k = n. \end{cases} \tag{113} $$

Proof. The three cases of Eq. (113) are considered separately.

1) Suppose that $k=0$. Then, Eq. (99) implies that ${}^{n+1}p_0$ can be expressed as follows:

$$ {}^{n+1}p_0 = {}^{n+1}\mathrm{u}_0 \, W^{-\frac{0^2}{2}} = {}^{n+1}\mathrm{u}_0. \tag{114}$$

Using Lemma 6 this derivation can be continued as follows:

$$ {}^{n+1}p_0 = {}^{n}\mathrm{u}_0 \, \frac{W^{n-0}}{W^{n-0}-1} = \underbrace{{}^{n}\mathrm{u}_0 \, W^{-\frac{0^2}{2}}}_{{}^{n}p_0} \frac{W^n}{W^n-1}, \tag{115}$$

which proves the first case in Eq. (113).

2) Suppose that $k \in \{1,2,\ldots,n-1\}$. Then, Lemma 8 and Eq. (99) imply that ${}^{n+1}p_k$ can be expressed as follows:

$$ {}^{n+1}p_k = {}^{n+1}\mathrm{u}_k \, W^{-\frac{k^2}{2}} = \frac{{}^{n}\mathrm{u}_k \, W^n - {}^{n}\mathrm{u}_{k-1} \, W^{k-\frac{1}{2}}}{W^n-1} \, W^{-\frac{k^2}{2}}. \tag{116}$$

Using basic algebra, this can be simplified as follows:

$$ \begin{aligned} {}^{n+1}p_k &= \frac{{}^{n}\mathrm{u}_k \, W^{-\frac{k^2}{2}} \, W^n - {}^{n}\mathrm{u}_{k-1} W^{-\frac{k^2}{2}+k-\frac{1}{2}}}{W^n-1} \\ &= \frac{{}^{n}p_k \, W^n - {}^{n}\mathrm{u}_{k-1} W^{-\frac{(k-1)^2}{2}}}{W^n-1} \\ &= \frac{{}^{n}p_k \, W^n - {}^{n}p_{k-1}}{W^n-1}. \end{aligned} \tag{117}$$

3) Suppose that $k=n$. Then, using Eqs. (81) and (99), ${}^{n+1}p_n$ can be expressed as follows:

$$ \begin{aligned} {}^{n+1}p_n &= {}^{n+1}\mathrm{u}_n W^{-\frac{n^2}{2}} \\ &= (-1)^n \frac{W^{\frac{2n^2-(2(n+1)-1)n+(n+1)(n+1-1)}{2}}}{\prod_{s=1}^{(n+1)-n-1}(W^s-1) \prod_{s=1}^{n}(W^s-1)} W^{-\frac{n^2}{2}} \\ &= (-1)^1(-1)^{n-1} \frac{W^{\frac{n^2}{2}}}{\prod_{s=1}^{n}(W^s-1)} W^{-\frac{n^2}{2}}. \end{aligned} \tag{118}$$

Because $n^2 = (n-1)^2 + 2n - 1$, this derivation can be continued as shown below:

$$ \begin{aligned} {}^{n+1}p_n &= -\frac{(-1)^{n-1} W^{\frac{(n-1)^2}{2}} \, W^{\frac{(2n-1)}{2}}}{(W^n-1)\prod_{s=1}^{n-1}(W^s-1)} W^{-\frac{(n-1)^2}{2}} \, W^{-\frac{(2n-1)}{2}} \\ &= -\underbrace{\frac{(-1)^{n-1} W^{\frac{2(n-1)^2-(2n-1)(n-1)+n(n-1)}{2}}}{\prod_{s=1}^{n-(n-1)-1}(W^s-1) \prod_{s=1}^{n-1}(W^s-1)}}_{{}^{n}\mathrm{u}_{n-1}} \cdot \frac{W^{-\frac{(n-1)^2}{2}}}{W^n-1}. \end{aligned} \tag{119}$$

Therefore,

$$ {}^{n+1}p_n = -\frac{{}^{n}\mathrm{u}_{n-1} \, W^{-\frac{(n-1)^2}{2}}}{W^n-1} = -\frac{{}^{n}p_{n-1}}{W^n-1}. \tag{120}$$

□

The following theorem completes the inductive argument by showing that ${}^{n}L(x) = {}^{n}P(x)$ for each integer n.

Theorem 12. *For each $n \in \mathbb{N}$ and each $x \in \mathbb{C}$, the values of ${}^{n}L(x)$ and ${}^{n}P(x)$ are equal, i.e.,*

$$ {}^{n}L(x) = {}^{n}P(x). \tag{121}$$

Proof. The proof is by mathematical induction. Lemma 9 proved Eq. (121) for $n=1$, which forms the base case of the induction. The inductive step is formed by Lemma 11 and Lemma 10, which showed that ${}^{n+1}L(x)$ can be derived from the coefficients of ${}^{n}L(x)$ using the same formula that derives ${}^{n+1}P(x)$ from the coefficients of ${}^{n}P(x)$. This implies that ${}^{n+1}P(x) = {}^{n+1}L(x)$ whenever ${}^{n}L(x) = {}^{n}P(x)$.

Taken together, the base case and the inductive step complete the inductive proof and show that ${}^{n}L(x) = {}^{n}P(x)$ for each $n \in \mathbb{N}$ and each $x \in \mathbb{C}$. □

The next theorem combines the results from this section to show that the vector $\mathbf{u}$ is indeed the first column of the inverse matrix $\hat{\boldsymbol{W}}^{-1}$. This result follows from Theorem 12 when x is a power of W, i.e., $x \in \{W^0, W^1, W^2, \ldots, W^{n-1}\}$.

Theorem 13. *The vector $\mathbf{u} = (\mathrm{u}_0, \mathrm{u}_1, \ldots, \mathrm{u}_{n-1})$ is equal to the first column of the inverse matrix $\hat{\boldsymbol{W}}^{-1}$, i.e.,*

$$ \hat{\boldsymbol{W}}\mathbf{u} = \begin{bmatrix} W^{-\frac{(0-0)^2}{2}} & W^{-\frac{(0-1)^2}{2}} & \ldots & W^{-\frac{(0-(n-1))^2}{2}} \\ W^{-\frac{(1-0)^2}{2}} & W^{-\frac{(1-1)^2}{2}} & \ldots & W^{-\frac{(1-(n-1))^2}{2}} \\ \vdots & \vdots & \ddots & \vdots \\ W^{-\frac{((n-1)-0)^2}{2}} & W^{-\frac{((n-1)-1)^2}{2}} & \ldots & W^{-\frac{((n-1)-(n-1))^2}{2}} \end{bmatrix} \begin{bmatrix} \mathrm{u}_0 \\ \mathrm{u}_1 \\ \vdots \\ \mathrm{u}_{n-1} \end{bmatrix} = \begin{bmatrix} 1 \\ 0 \\ \vdots \\ 0 \end{bmatrix}. \tag{122}$$

Proof. Eq. (122) can be restated as follows:

$$ (\hat{\boldsymbol{W}}\mathbf{u})_j = \sum_{k=0}^{n-1} \mathrm{u}_k \, W^{-\frac{(k-j)^2}{2}} = \begin{cases} 1, & \text{if } j=0, \\ 0, & \text{if } j \in \{1,2,\ldots,n-1\}. \end{cases} \tag{123}$$

The sum in Eq. (123) can be expressed using ${}^{n}P(W^j)$, i.e.,

$$ \begin{aligned} \sum_{k=0}^{n-1} \mathrm{u}_k \, W^{-\frac{(k-j)^2}{2}} &= \sum_{k=0}^{n-1} \mathrm{u}_k \, W^{-\frac{k^2}{2}} (W^j)^k \, W^{-\frac{j^2}{2}} \\ &= {}^{n}P(W^j) \, W^{-\frac{j^2}{2}}. \end{aligned} \tag{124}$$

Theorem 12 showed that ${}^{n}P(x) = {}^{n}L(x)$. Therefore,

$$ \begin{aligned} (\hat{\boldsymbol{W}}\mathbf{u})_j &= {}^{n}P(W^j) \, W^{-\frac{j^2}{2}} = {}^{n}L(W^j) \, W^{-\frac{j^2}{2}} \\ &= \prod_{k=1}^{n-1} \frac{(W^j - W^k)}{(1-W^k)} \, W^{-\frac{j^2}{2}}. \end{aligned} \tag{125}$$

If $j=0$, then Eq. (125) evaluates to 1. Alternatively, if $j \in \{1,2,\ldots,n-1\}$, then it evaluates to 0 because there is at least one zero factor in the product. More formally,

$$ (\hat{\boldsymbol{W}}\mathbf{u})_j = \prod_{k=1}^{n-1} \frac{(W^j - W^k)}{(1-W^k)} W^{-\frac{j^2}{2}} = \begin{cases} 1, & \text{if } j=0, \\ 0, & \text{if } 1 \le j \le n-1, \end{cases} \tag{126}$$

which shows that Eq. (122) holds. □

Supplementary Appendix G: CZT and ICZT Algorithms that Reverse the Contour Direction When $|W| < 1$

This appendix describes alternative versions of the CZT and the ICZT algorithms that improve the numerical accuracy of the computed transforms. This is achieved by reversing the direction of the chirp contour when $|W| < 1$ and keeping the original direction when $|W| \geq 1$. The parameters for the reversed contour are $W' = W^{-1}$ and $A' = A\,W^{-(M-1)}$.

This appendix also proves that this parameter adjustment does not affect the mathematical definitions of the CZT and the ICZT. In other words, the modified algorithms compute the same transforms, but the numerical precision of their results can improve by several orders of magnitude when the magnitude of the transform parameter W is less than 1.

The following lemma shows that the Vandermonde matrix $\boldsymbol{W}$ associated with the parameter W can be mapped to the Vandermonde matrix $\boldsymbol{W}'$ associated with $W' = W^{-1}$.

Lemma 14. *Let W be a non-zero complex number and let $\boldsymbol{W}$ be the M-by-N Vandermonde matrix defined by* Eq. (3)*, i.e., the element in the i-th row and j-th column of $\boldsymbol{W}$ is:*

$$\mathrm{W}_{i,j} = W^{ij}, \tag{127}$$

for each $i \in \{0, 1, \ldots, M-1\}$ and each $j \in \{0, 1, \ldots, N-1\}$. Also, let $\boldsymbol{W}'$ be the Vandermonde matrix specified by the same formula but using W^{-1} instead of W. That is,

$$\boldsymbol{W}' = \begin{bmatrix} W^{-0\cdot 0} & W^{-1\cdot 0} & W^{-2\cdot 0} & \cdots & W^{-(N-1)\cdot 0} \\ W^{-0\cdot 1} & W^{-1\cdot 1} & W^{-2\cdot 1} & \cdots & W^{-(N-1)\cdot 1} \\ W^{-0\cdot 2} & W^{-1\cdot 2} & W^{-2\cdot 2} & \cdots & W^{-(N-1)\cdot 2} \\ \vdots & \vdots & \vdots & \ddots & \vdots \\ W^{-0\cdot(M-1)} & W^{-1\cdot(M-1)} & W^{-2\cdot(M-1)} & \cdots & W^{-(N-1)\cdot(M-1)} \end{bmatrix}. \tag{128}$$

Furthermore, let $\boldsymbol{S}$ be the N-by-N diagonal matrix generated by the last row of the matrix $\boldsymbol{W}$, i.e.,

$$\boldsymbol{S} = \operatorname{diag}\left(W^{0\cdot(M-1)}, W^{1\cdot(M-1)}, \ldots, W^{(N-1)\cdot(M-1)}\right). \tag{129}$$

Then, the matrix $\boldsymbol{W}$ can be expressed as the following triple matrix product:

$$\boldsymbol{W} = \boldsymbol{J}\,\boldsymbol{W}'\,\boldsymbol{S}, \tag{130}$$

where $\boldsymbol{J}$ denotes the M-by-M exchange matrix, i.e.,

$$\boldsymbol{J} = \begin{bmatrix} 0 & \cdots & 0 & 1 \\ 0 & \cdots & 1 & 0 \\ \vdots & ⋰ & \vdots & \vdots \\ 1 & \cdots & 0 & 0 \end{bmatrix}. \tag{131}$$

Proof. The matrix product $\boldsymbol{W}'\boldsymbol{S}$ has the following form:

$$\boldsymbol{W}'\boldsymbol{S} = \begin{bmatrix} W^{0\cdot(M-1)} & W^{1\cdot(M-1)} & W^{2\cdot(M-1)} & \cdots & W^{(N-1)\cdot(M-1)} \\ W^{0\cdot(M-2)} & W^{1\cdot(M-2)} & W^{2\cdot(M-2)} & \cdots & W^{(N-1)\cdot(M-2)} \\ W^{0\cdot(M-3)} & W^{1\cdot(M-3)} & W^{2\cdot(M-3)} & \cdots & W^{(N-1)\cdot(M-3)} \\ \vdots & \vdots & \vdots & \ddots & \vdots \\ W^{0\cdot 0} & W^{1\cdot 0} & W^{2\cdot 0} & \cdots & W^{(N-1)\cdot 0} \end{bmatrix}, \tag{132}$$

which follows from the definitions of $\boldsymbol{W}'$ and $\boldsymbol{S}$. Each element of this matrix is given by the following formula:

$$(\boldsymbol{W}'\boldsymbol{S})_{i,j} = \sum_{k=0}^{N-1} \mathrm{W}'_{i,k}\,\mathrm{S}_{k,j} = W^{-ij}\,W^{j(M-1)} = W^{j(M-1-i)}, \tag{133}$$

for each $i \in \{0, 1, \ldots, M-1\}$ and each $j \in \{0, 1, \ldots, N-1\}$.

This implies that the matrix product $\boldsymbol{W}'\boldsymbol{S}$ is equal to the matrix obtained by reversing the rows of the matrix $\boldsymbol{W}$. This operation can be formalized using the exchange matrix $\boldsymbol{J}$, i.e.,

$$\boldsymbol{J}\boldsymbol{W} = \boldsymbol{W}'\boldsymbol{S}. \tag{134}$$

Because the matrix $\boldsymbol{J}$ is its own inverse, it follows that Eq. (130) can be derived by pre-multiplying both sides of the previous equation by $\boldsymbol{J}$, i.e., $\boldsymbol{W} = \boldsymbol{J}\boldsymbol{J}\boldsymbol{W} = \boldsymbol{J}\boldsymbol{W}'\boldsymbol{S}$. □

The next lemma shows how the diagonal matrix $\mathbf{A}$, which is associated with the parameter A, can be mapped to the diagonal matrix $\mathbf{A}'$ that is associated with $A' = A\,W^{-(M-1)}$.

Lemma 15. *Let A and W be two non-zero complex numbers. Let $\mathbf{A}$ be the diagonal matrix defined by* Eq. (8)*, i.e.,*

$$\mathbf{A} = \operatorname{diag}\left(A^{-0}, A^{-1}, \ldots, A^{-(N-1)}\right). \tag{135}$$

Also, let $\mathbf{A}'$ be the following N-by-N diagonal matrix:

$$\mathbf{A}' = \operatorname{diag}\left(A^{-0}\,W^{0(M-1)}, \ldots, A^{-(N-1)}\,W^{(N-1)(M-1)}\right). \tag{136}$$

Then, the matrix $\mathbf{A}'$ can be expressed as the product of the matrix $\mathbf{A}$ and the matrix $\boldsymbol{S}$ defined by Eq. (129)*, i.e.,*

$$\mathbf{A}' = \boldsymbol{S}\,\mathbf{A} = \mathbf{A}\,\boldsymbol{S}. \tag{137}$$

Proof. Both $\mathbf{A}$ and $\boldsymbol{S}$ are diagonal matrices. This implies that both matrices are invariant with respect to transposition. Thus,

$$\boldsymbol{S}\,\mathbf{A} = \boldsymbol{S}^T\mathbf{A}^T = (\mathbf{A}\,\boldsymbol{S})^T = \mathbf{A}\,\boldsymbol{S}. \tag{138}$$

In other words, each diagonal element of the matrix product $\boldsymbol{S}\,\mathbf{A}$ is equal to the product of the corresponding elements of $\boldsymbol{S}$ and $\mathbf{A}$; all other elements of $\boldsymbol{S}\,\mathbf{A}$ are zero. Thus,

$$(\boldsymbol{S}\,\mathbf{A})_{i,j} = \begin{cases} A^{-j}\,W^{j(M-1)}, & \text{if } i = j, \\ 0, & \text{if } i \neq j. \end{cases} \tag{139}$$

Therefore, $\boldsymbol{S}\,\mathbf{A} = \mathbf{A}'$, which completes the proof. □

The following theorem proves that computing the CZT without contour reversal is mathematically equivalent to computing the CZT with contour reversal and then reversing the order of the elements in the output vector.

Theorem 16. *Let M and N be two positive integers and let $A, W \in \mathbb{C} \setminus \{0\}$ be two non-zero complex numbers. Let $\mathbf{x} = (\mathrm{x}_0, \mathrm{x}_1, \mathrm{x}_2, \ldots, \mathrm{x}_{N-1}) \in \mathbb{C}^N$ be a complex input vector for a CZT that is parametrized by M, W, and A. Let $\mathbf{X}$ be the CZT output vector. More formally,*

$$\mathbf{X} = \mathrm{CZT}(\mathbf{x}, M, W, A). \tag{140}$$

Let $A' = A\,W^{-(M-1)}$ and $W' = W^{-1}$ be the parameters for the reversed chirp contour. Let $\mathbf{X}'$ be the output vector of a CZT that is parametrized by M, W', and A'. That is,

$$\mathbf{X}' = \mathrm{CZT}(\mathbf{x},\, M,\, W',\, A'\,). \tag{141}$$

Then, $\mathbf{X}$ can be obtained by reversing the order of the elements in $\mathbf{X}'$. More formally,

$$\mathbf{X} = (\mathrm{X}_0, \mathrm{X}_1, \ldots, \mathrm{X}_{M-1}) = (\mathrm{X}'_{M-1}, \mathrm{X}'_{M-2}, \ldots, \mathrm{X}'_0) = \boldsymbol{J}\mathbf{X}'. \tag{142}$$

In other words, reversing the direction of the chirp contour reverses the order of the elements in the output vector.

Proof. Eq. (140) has the following matrix form:

$$\mathbf{X} = \boldsymbol{W}\,\mathbf{A}\,\mathbf{x}, \tag{143}$$

where $\boldsymbol{W}$ is the Vandermonde matrix defined by Eq. (3) and $\mathbf{A}$ is the diagonal matrix defined by equation Eq. (8). Similarly, the vector $\mathbf{X}'$ can be expressed as follows:

$$\mathbf{X}' = \boldsymbol{W}'\,\mathbf{A}'\,\mathbf{x}. \tag{144}$$

From Lemma 14 it follows that the matrix $\boldsymbol{W}$ in Eq. (143) can be replaced with the matrix product $\boldsymbol{J}\,\mathbf{W}'\,\boldsymbol{S}$, i.e.,

$$\mathbf{X} = \boldsymbol{J}\,\boldsymbol{W}'\,\boldsymbol{S}\,\mathbf{A}\,\mathbf{x}. \tag{145}$$

From Lemma 15 it follows that $\mathbf{A} = \boldsymbol{S}^{-1}\,\mathbf{A}'$. Therefore,

$$\mathbf{X} = \boldsymbol{J}\,\boldsymbol{W}'\cancel{\boldsymbol{S}}\cancel{\boldsymbol{S}^{-1}}\mathbf{A}'\,\mathbf{x} = \boldsymbol{J}\,\boldsymbol{W}'\mathbf{A}'\,\mathbf{x} = \boldsymbol{J}\,\mathbf{X}', \tag{146}$$

which proves that the vector $\mathbf{X}$ can be obtained by reversing the elements of the vector $\mathbf{X}'$. □

The next theorem proves that computing the ICZT without contour reversal is theoretically equivalent to computing the ICZT with reversal of both the contour and the input vector. The theorem is stated for the square case, i.e., when $M = N$.

Theorem 17. *Let N be a positive integer and let A and W be two non-zero complex numbers. Let $\mathbf{X} = (\mathrm{X}_0, \mathrm{X}_1, \ldots, \mathrm{X}_{N-1})$ be a complex vector that is used as an input to an ICZT parametrized by N, W, and A. Let $\mathbf{x}$ be the result of this transform. More formally,*

$$\mathbf{x} = \mathrm{ICZT}(\mathbf{X}, N, W, A). \tag{147}$$

Let $A' = A\,W^{-(M-1)}$ and $W' = W^{-1}$ be the parameters for the reversed chirp contour, where $M = N$. Let $\mathbf{x}'$ be the output vector of the ICZT parameterized by N, W', and A' for which the input vector is set to $\boldsymbol{J}\mathbf{X}$. That is,

$$\mathbf{x}' = \mathrm{ICZT}(\boldsymbol{J}\mathbf{X}, N, W', A'\,). \tag{148}$$

Then, $\mathbf{x}$ and $\mathbf{x}'$ are equal, i.e., $\mathbf{x} = \mathbf{x}'$.

Proof. This result can be proven by expressing $\mathbf{x}$ and $\mathbf{x}'$ in matrix form and showing that the two expressions are equal.

From Eqs. (130) and (137) it follows that $\boldsymbol{W}' = \boldsymbol{J}\,\boldsymbol{W}\,\boldsymbol{S}^{-1}$ and $\mathbf{A}' = \boldsymbol{S}\,\mathbf{A}$. Thus, the vector $\mathbf{x}'$ is equal to:

$$\begin{aligned}\mathbf{x}' &= (\mathbf{A}')^{-1}(\boldsymbol{W}')^{-1}\boldsymbol{J}\,\mathbf{X}\\ &= (\boldsymbol{S}\,\mathbf{A})^{-1}(\boldsymbol{J}\,\boldsymbol{W}\,\boldsymbol{S}^{-1})^{-1}\boldsymbol{J}\mathbf{X}\\ &= \mathbf{A}^{-1}\cancel{\boldsymbol{S}^{-1}}\cancel{\boldsymbol{S}}\boldsymbol{W}^{-1}\cancel{\boldsymbol{J}}\cancel{\boldsymbol{J}}\mathbf{X}\\ &= \mathbf{A}^{-1}\boldsymbol{W}^{-1}\mathbf{X}.\end{aligned} \tag{149}$$

Similarly, the vector $\mathbf{x}$ can be derived by inverting the matrix expression for the CZT, i.e.,

$$\mathbf{x} = (\boldsymbol{W}\,\mathbf{A})^{-1}\mathbf{X} = \mathbf{A}^{-1}\boldsymbol{W}^{-1}\mathbf{X}. \tag{150}$$

This completes the proof, because Eqs. (149) and (150) are equal. □

Algorithm S8 gives the pseudo-code for the alternative version of the CZT algorithm that reverses the direction of the chirp contour when $|W| < 1$. It is implemented as a wrapper around Algorithm 1. Similarly, Algorithm S9 shows the pseudo-code for the alternative version of the ICZT algorithm, which also reflects the direction of the chirp contour when $|W| < 1$. The computational complexity of both algorithms remains unchanged, i.e., it is still $O(n \log n)$.

For improved numerical accuracy, lines 6 and 7 in both Algorithm S8 and Algorithm S9 should be computed with higher precision if possible. The reason is that even a small numerical error in the computed values of A' and W' affects all subsequent operations.

Algorithm S8. CZT algorithm that reverses the direction of the chirp contour when $|W| < 1$. Runs in $O(n \log n)$ time.

1: CZT–R($\mathbf{x}$, M, W, A)
2: **if** $|W| \geq 1$ **then**
3: $\quad\mathbf{X} \leftarrow \mathrm{CZT}(\mathbf{x},\, M,\, W,\, A)$; // original contour
4: **else**
5: $\quad$// Swap the start and the end point of the chirp contour.
6: $\quad A' \leftarrow A\,W^{-(M-1)}$;
7: $\quad W' \leftarrow 1/W$;
8: $\quad\mathbf{X}' \leftarrow \mathrm{CZT}(\mathbf{x},\, M,\, W',\, A'\,)$; // reversed contour
9: $\quad\mathbf{X} \leftarrow$ EmptyArray(M);
10: $\quad$**for** $k \leftarrow 0$ **to** $M-1$ **do** // reverse the output vector
11: $\quad\quad \mathrm{X}[\,k\,] \leftarrow \mathrm{X}'[M-1-k]$;
12: $\quad$**end for**
13: **end if**
14: **return** $\mathbf{X}$;

Algorithm S9. ICZT algorithm that reverses the direction of the chirp contour when $|W| < 1$. Runs in $O(n \log n)$ time.

1: ICZT–R($\mathbf{X}$, N, W, A)
2: $M \leftarrow$ Length($\mathbf{X}$);
3: **if** $|W| \geq 1$ **then**
4: $\quad\mathbf{x} \leftarrow \mathrm{ICZT}(\mathbf{X},\, N,\, W,\, A)$; // original contour
5: **else**
6: $\quad A' \leftarrow A\,W^{-(M-1)}$;
7: $\quad W' \leftarrow 1/W$;
8: $\quad\mathbf{X}' \leftarrow$ EmptyArray(M);
9: $\quad$**for** $k \leftarrow 0$ **to** $M-1$ **do** // reverse the input vector
10: $\quad\quad \mathrm{X}'[\,k\,] \leftarrow \mathrm{X}[M-1-k]$;
11: $\quad$**end for**
12: $\quad\mathbf{x} \leftarrow \mathrm{ICZT}(\mathbf{X}',\, N,\, W',\, A'\,)$; // reversed contour
13: **end if**
14: **return** $\mathbf{x}$;

The following example illustrates Theorem 16. In the 3-by-3 case, the CZT can be expressed as follows:

$$\underbrace{\begin{bmatrix} \mathrm{X}_0 \\ \mathrm{X}_1 \\ \mathrm{X}_2 \end{bmatrix}}_{\mathbf{X}} = \underbrace{\begin{bmatrix} 1 & 1 & 1 \\ 1 & W^1 & W^2 \\ 1 & W^2 & W^4 \end{bmatrix}}_{\boldsymbol{W}} \underbrace{\begin{bmatrix} A^{-0} & 0 & 0 \\ 0 & A^{-1} & 0 \\ 0 & 0 & A^{-2} \end{bmatrix}}_{\mathbf{A}} \underbrace{\begin{bmatrix} \mathrm{x}_0 \\ \mathrm{x}_1 \\ \mathrm{x}_2 \end{bmatrix}}_{\mathbf{x}}. \tag{151}$$

Previously, it was shown that $\boldsymbol{W} = \boldsymbol{J}\boldsymbol{W}'\boldsymbol{S}$, $\mathbf{A} = \boldsymbol{S}^{-1}\mathbf{A}'$, and $\mathbf{X}' = \boldsymbol{W}'\,\mathbf{A}'\,\mathbf{x}$. Thus, Eq. (151) can be stated as follows:

$$\begin{aligned}
\underbrace{\begin{bmatrix} \mathrm{X}_0 \\ \mathrm{X}_1 \\ \mathrm{X}_2 \end{bmatrix}}_{\mathbf{X}} &= \underbrace{\begin{bmatrix} 0 & 0 & 1 \\ 0 & 1 & 0 \\ 1 & 0 & 0 \end{bmatrix}}_{\boldsymbol{J}} \underbrace{\begin{bmatrix} 1 & 1 & 1 \\ 1 & W^{-1} & W^{-2} \\ 1 & W^{-2} & W^{-4} \end{bmatrix}}_{\boldsymbol{W}'} \underbrace{\begin{bmatrix} W^0 & 0 & 0 \\ 0 & W^2 & 0 \\ 0 & 0 & W^4 \end{bmatrix}}_{\boldsymbol{S}} \underbrace{\begin{bmatrix} W^{-0} & 0 & 0 \\ 0 & W^{-2} & 0 \\ 0 & 0 & W^{-4} \end{bmatrix}}_{\boldsymbol{S}^{-1}} \underbrace{\begin{bmatrix} A^{-0}\,W^0 & 0 & 0 \\ 0 & A^{-1}\,W^2 & 0 \\ 0 & 0 & A^{-2}\,W^4 \end{bmatrix}}_{\mathbf{A}'} \underbrace{\begin{bmatrix} \mathrm{x}_0 \\ \mathrm{x}_1 \\ \mathrm{x}_2 \end{bmatrix}}_{\mathbf{x}} \\
&= \underbrace{\begin{bmatrix} 0 & 0 & 1 \\ 0 & 1 & 0 \\ 1 & 0 & 0 \end{bmatrix}}_{\boldsymbol{J}} \underbrace{\begin{bmatrix} 1 & 1 & 1 \\ 1 & W^{-1} & W^{-2} \\ 1 & W^{-2} & W^{-4} \end{bmatrix}}_{\boldsymbol{W}'} \underbrace{\begin{bmatrix} A^{-0}\,W^0 & 0 & 0 \\ 0 & A^{-1}\,W^2 & 0 \\ 0 & 0 & A^{-2}\,W^4 \end{bmatrix}}_{\mathbf{A}'} \underbrace{\begin{bmatrix} \mathrm{x}_0 \\ \mathrm{x}_1 \\ \mathrm{x}_2 \end{bmatrix}}_{\mathbf{x}} \\
&= \underbrace{\begin{bmatrix} 0 & 0 & 1 \\ 0 & 1 & 0 \\ 1 & 0 & 0 \end{bmatrix}}_{\boldsymbol{J}} \underbrace{\begin{bmatrix} \mathrm{X}'_0 \\ \mathrm{X}'_1 \\ \mathrm{X}'_2 \end{bmatrix}}_{\mathbf{X}'}.
\end{aligned} \tag{152}$$

To summarize, if the CZT is computed for a reversed chirp contour, i.e., $\mathbf{X}' = \boldsymbol{W}'\,\mathbf{A}'\,\mathbf{x}$, then the output vector $\mathbf{X} = \boldsymbol{W}\,\mathbf{A}\,\mathbf{x}$ for the original contour can be computed by reversing the order of the elements in the vector $\mathbf{X}'$, i.e., $\mathbf{X} = \boldsymbol{J}\,\mathbf{X}'$.

The next example illustrates Theorem 17, which showed that $\mathbf{x}' = (\mathbf{A}')^{-1}\,(\boldsymbol{W}')^{-1}\,\boldsymbol{J}\,\mathbf{X} = \mathbf{A}^{-1}\,\boldsymbol{W}^{-1}\mathbf{X} = \mathbf{x}$. For the 3-by-3 ICZT case, this equation has the following form:

$$\begin{aligned}
\underbrace{\begin{bmatrix} \mathrm{x}'_0 \\ \mathrm{x}'_1 \\ \mathrm{x}'_2 \end{bmatrix}}_{\mathbf{x}'} &= \underbrace{\begin{bmatrix} A^0\,W^{-0} & 0 & 0 \\ 0 & A^1\,W^{-2} & 0 \\ 0 & 0 & A^2\,W^{-4} \end{bmatrix}}_{(\mathbf{A}')^{-1}} \underbrace{\begin{bmatrix} (\boldsymbol{W}')^{-1}_{1,1} & (\boldsymbol{W}')^{-1}_{1,2} & (\boldsymbol{W}')^{-1}_{1,3} \\ (\boldsymbol{W}')^{-1}_{2,1} & (\boldsymbol{W}')^{-1}_{2,2} & (\boldsymbol{W}')^{-1}_{2,3} \\ (\boldsymbol{W}')^{-1}_{3,1} & (\boldsymbol{W}')^{-1}_{3,2} & (\boldsymbol{W}')^{-1}_{3,3} \end{bmatrix}}_{(\boldsymbol{W}')^{-1}} \underbrace{\begin{bmatrix} 0 & 0 & 1 \\ 0 & 1 & 0 \\ 1 & 0 & 0 \end{bmatrix}}_{\boldsymbol{J}} \underbrace{\begin{bmatrix} \mathrm{X}_0 \\ \mathrm{X}_1 \\ \mathrm{X}_2 \end{bmatrix}}_{\mathbf{X}} \\
&= \underbrace{\begin{bmatrix} A^0 & 0 & 0 \\ 0 & A^1 & 0 \\ 0 & 0 & A^2 \end{bmatrix}}_{\mathbf{A}^{-1}} \underbrace{\begin{bmatrix} W^{-0} & 0 & 0 \\ 0 & W^{-2} & 0 \\ 0 & 0 & W^{-4} \end{bmatrix}}_{\boldsymbol{S}^{-1}} \underbrace{\begin{bmatrix} W^0 & 0 & 0 \\ 0 & W^2 & 0 \\ 0 & 0 & W^4 \end{bmatrix}}_{\boldsymbol{S}} \underbrace{\begin{bmatrix} \boldsymbol{W}^{-1}_{1,1} & \boldsymbol{W}^{-1}_{1,2} & \boldsymbol{W}^{-1}_{1,3} \\ \boldsymbol{W}^{-1}_{2,1} & \boldsymbol{W}^{-1}_{2,2} & \boldsymbol{W}^{-1}_{2,3} \\ \boldsymbol{W}^{-1}_{3,1} & \boldsymbol{W}^{-1}_{3,2} & \boldsymbol{W}^{-1}_{3,3} \end{bmatrix}}_{\boldsymbol{W}^{-1}} \underbrace{\begin{bmatrix} 0 & 0 & 1 \\ 0 & 1 & 0 \\ 1 & 0 & 0 \end{bmatrix}}_{\boldsymbol{J}} \underbrace{\begin{bmatrix} 0 & 0 & 1 \\ 0 & 1 & 0 \\ 1 & 0 & 0 \end{bmatrix}}_{\boldsymbol{J}} \underbrace{\begin{bmatrix} \mathrm{X}_0 \\ \mathrm{X}_1 \\ \mathrm{X}_2 \end{bmatrix}}_{\mathbf{X}} \\
&= \underbrace{\begin{bmatrix} A^0 & 0 & 0 \\ 0 & A^1 & 0 \\ 0 & 0 & A^2 \end{bmatrix}}_{\mathbf{A}^{-1}} \underbrace{\begin{bmatrix} \boldsymbol{W}^{-1}_{1,1} & \boldsymbol{W}^{-1}_{1,2} & \boldsymbol{W}^{-1}_{1,3} \\ \boldsymbol{W}^{-1}_{2,1} & \boldsymbol{W}^{-1}_{2,2} & \boldsymbol{W}^{-1}_{2,3} \\ \boldsymbol{W}^{-1}_{3,1} & \boldsymbol{W}^{-1}_{3,2} & \boldsymbol{W}^{-1}_{3,3} \end{bmatrix}}_{\boldsymbol{W}^{-1}} \underbrace{\begin{bmatrix} \mathrm{X}_0 \\ \mathrm{X}_1 \\ \mathrm{X}_2 \end{bmatrix}}_{\mathbf{X}} = \underbrace{\begin{bmatrix} \mathrm{x}_0 \\ \mathrm{x}_1 \\ \mathrm{x}_2 \end{bmatrix}}_{\mathbf{x}}.
\end{aligned} \tag{153}$$

This result follows from $(\mathbf{A}')^{-1} = \mathbf{A}^{-1}\,\boldsymbol{S}^{-1}$ and $(\boldsymbol{W}')^{-1} = (\boldsymbol{J}\,\boldsymbol{W}\boldsymbol{S}^{-1})^{-1} = \boldsymbol{S}\,\boldsymbol{W}^{-1}\boldsymbol{J}$, and also from the fact that the matrix $\boldsymbol{J}$ is its own inverse. In other words, when computing the ICZT for the reversed chirp contour, the elements of the input vector need to be reversed in order to get the same result as the ICZT for the original chirp contour.

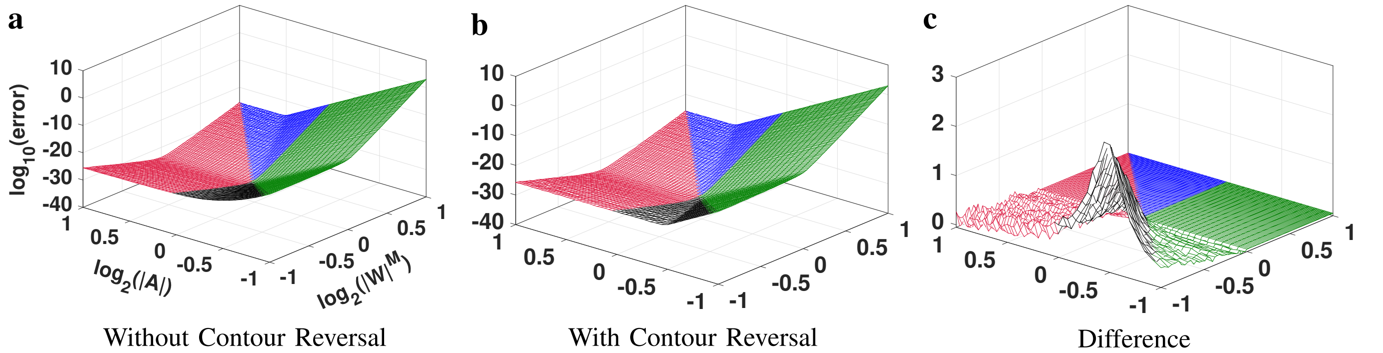

Figure S2. Absolute numerical error of the CZT algorithm, computed without contour reversal (**a**) and with contour reversal (**b**). The difference between (**a**) and (**b**) is shown in (**c**). The surfaces were generated for $M = 64$ as functions of the transform parameters A and W, which were discretized as described in the Methods section. Each of the 5,200 points on each surface was computed by averaging the numerical errors over 10 random unit-length input vectors.

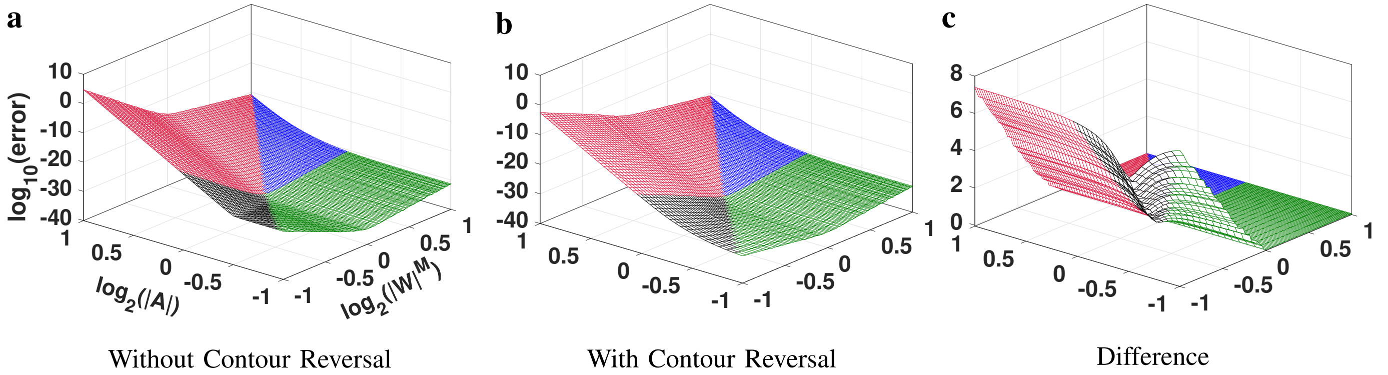

Figure S3. Absolute numerical error of the ICZT algorithm, computed without contour reversal (**a**) and with contour reversal (**b**). The difference between (**a**) and (**b**) is shown in (**c**). The surfaces were generated for $M = 64$ as functions of the transform parameters A and W, which were discretized as described in the Methods section. Each of the 5,200 points on each surface was computed by averaging the numerical errors over 10 random unit-length input vectors.

Figure S2 compares the numerical accuracy of the CZT algorithm with and without contour reversal. The surface in Fig. S2a was computed with Algorithm 1, which does not reverse the contour. The surface in Fig. S2b was computed with Algorithm S8, which reverses the direction of the chirp contour when $|W| < 1$. The difference between these two surfaces is shown in Fig. S2c, where positive values indicate that the error in (a) is higher than the error in (b).

The numerical error is equal to $\|\hat{\mathbf{X}} - \mathbf{X}\|$, where $\hat{\mathbf{X}}$ is the computed output vector and $\mathbf{X}$ is the true but unknown output vector. In this experiment, $\hat{\mathbf{X}}$ was computed using 128-bit floating-point numbers and $\mathbf{X}$ was computed with 1024-bit numbers. In both cases, the mpmath library[34] was used to software-emulate these high-precision floating-point numbers. The values of $\mathbf{X}$ for both Fig. S2a and Fig. S2b were computed with Algorithm 1, i.e., without contour reversal.

Figure S3 summarizes the results of a similar experiment for the ICZT. The error is given by $\|\hat{\mathbf{x}} - \mathbf{x}\|$, where $\hat{\mathbf{x}}$ is the computed output vector and $\mathbf{x}$ is the true but unknown output vector. The value of $\hat{\mathbf{x}}$ was computed with 128-bit precision in Fig. S3a and Fig. S3b, using Algorithm 2 and Algorithm S9, respectively. For both of these figures, $\mathbf{x}$ was computed with 1024-bit precision using only Algorithm 2, i.e., ICZT without contour reversal. Figure S3c shows the pointwise difference between the two surfaces, i.e., $(\mathbf{c}) = (\mathbf{a}) - (\mathbf{b})$. The difference is zero when $|W| > 1$. For contours with $|W| < 1$, however, the difference is positive and can be as high as 7 orders of magnitude in this case, i.e., when $M = 64$.

These results may be explained by an asymmetry in the condition number of the matrix $\hat{\boldsymbol{W}}$ (see Eq. (9)). For $|W| < 1$, the condition number grows much faster than for $|W| > 1$.

In log space, the contour reversal can be expressed as:

$$\begin{bmatrix} \log W' \\ \log A' \end{bmatrix} = \begin{bmatrix} -1 & 0 \\ -(M-1) & 1 \end{bmatrix} \begin{bmatrix} \log W \\ \log A \end{bmatrix}. \tag{154}$$

The eigenvectors of this matrix are $(0, 1)$ and $(1, \frac{M-1}{2})$. The surfaces in Figs. S2 and S3 are bent along two lines that correspond to these two eigenvectors. The first line is $\log_2 |W|^M = 0$; the second line is $\log_2 |A| = \frac{M-1}{2M} \log_2 |W|^M$.

To summarize, this appendix showed that the numerical accuracy of both the CZT and the ICZT can be improved by several orders of magnitude for chirp contours that are growing logarithmic spirals, i.e., when $|W| < 1$. In those cases, the revised algorithms reverse the direction of the chirp contour. Furthermore, this contour reversal must be accompanied by a reversal of the order of the elements in the vector $\mathbf{X}$, which is either the CZT output vector or the ICZT input vector.

To make the contour reversal rules easier to remember, one can apply the following guidelines that use the four contour colors from Fig. 4. A blue contour should never be reversed because it is always a decaying logarithmic spiral. A green contour should be reversed only if it describes a growing spiral and left unchanged if it describes a decaying spiral. Similarly, a red contour should be reversed if it describes a growing spiral and left unchanged otherwise. Finally, a black contour (i.e., one that starts inside the unit circle and ends outside the unit circle) should always be reversed, which transforms it into a blue contour. In short, all chirp contours that are growing spirals should be reversed.

Supplementary Appendix H: Link Between the Polar Angle of the Parameter A and the Vector $\mathbf{x}$ in Both the CZT and the ICZT

Theorem 18. *Let W be a non-zero complex number. Also, let A and B be two non-zero complex numbers such that $|A| = |B| = \mu$. Moreover, let $\alpha = \arg(A)$ be the polar angle of A and let $\beta = \arg(B)$ be the polar angle of B, i.e.,*

$$A = |A|\, e^{i\alpha} = \mu\, e^{i\alpha}, \tag{155}$$

$$B = |B|\, e^{i\beta} = \mu\, e^{i\beta}. \tag{156}$$

Finally, let M and N be two positive integers.

Let $\mathbf{x} = (x_0, x_1, x_2, \ldots, x_{N-1}) \in \mathbb{C}^N$ be a complex vector of length N. Let $\mathbf{X}^A = (X_0^A, X_1^A, X_2^A, \ldots, X_{M-1}^A) \in \mathbb{C}^M$ be the complex vector of length M that is equal to the CZT of $\mathbf{x}$ parametrized by M, W, and A. More formally,

$$\mathbf{X}^A = \mathrm{CZT}(\mathbf{x}, M, W, A) = \boldsymbol{W}\, \mathbf{A}\, \mathbf{x}, \tag{157}$$

where $\boldsymbol{W}$ is the Vandermonde matrix generated by the parameter W as defined by Eq. (3) *and $\mathbf{A}$ is the diagonal matrix generated by the first N negative integer powers of the parameter A starting from A^{-0}, i.e.,*

$$\mathbf{A} = \mathrm{diag}\left(A^{-0}, A^{-1}, A^{-2}, \ldots, A^{-(N-1)}\right). \tag{158}$$

Similarly, let $\mathbf{X}^B = (X_0^B, X_1^B, X_2^B, \ldots, X_{M-1}^B) \in \mathbb{C}^M$ be the complex vector of size M that is equal to the CZT of $\mathbf{x}$ parametrized by M, W, and B. That is,

$$\mathbf{X}^B = \mathrm{CZT}(\mathbf{x}, M, W, B) = \boldsymbol{W}\, \mathbf{B}\, \mathbf{x}, \tag{159}$$

where $\mathbf{B}$ is a diagonal matrix generated by the first N negative integer powers of the parameter B, i.e.,

$$\mathbf{B} = \mathrm{diag}\left(B^{-0}, B^{-1}, B^{-2}, \ldots, B^{-(N-1)}\right). \tag{160}$$

Then, the vector $\mathbf{X}^B$ can be expressed as the CZT of the vector $\mathbf{x}' = \mathbf{D}\,\mathbf{x}$ where the transform is parametrized by M, W, and A and where the diagonal matrix $\mathbf{D}$ is defined as follows:

$$\mathbf{D} = \mathrm{diag}\left(e^{0i(\alpha-\beta)}, e^{1i(\alpha-\beta)}, \ldots, e^{(N-1)i(\alpha-\beta)}\right). \tag{161}$$

In other words,

$$\mathbf{X}^B = \mathrm{CZT}(\mathbf{D}\,\mathbf{x}, M, W, A) = \boldsymbol{W}\,\mathbf{A}\,\mathbf{D}\,\mathbf{x}. \tag{162}$$

Proof. The proof follows from the definitions of the matrices $\mathbf{A}$ and $\mathbf{B}$. Combining Eqs. (158) and (155) leads to:

$$\mathbf{A} = \mathrm{diag}\left(\mu^{-0} e^{-0i\alpha}, \mu^{-1} e^{-1i\alpha}, \ldots, \mu^{-(N-1)} e^{-(N-1)i\alpha}\right). \tag{163}$$

Similarly, combining Eqs. (160) and (156) leads to:

$$\mathbf{B} = \mathrm{diag}\left(\mu^{-0} e^{-0i\beta}, \mu^{-1} e^{-1i\beta}, \ldots, \mu^{-(N-1)}\, e^{-(N-1)i\beta}\right). \tag{164}$$

Therefore, $\mathbf{B} = \mathbf{A}\mathbf{D}$. This implies that:

$$\mathbf{X}^B = \boldsymbol{W}\,\mathbf{B}\,\mathbf{x} = \boldsymbol{W}\,\mathbf{A}\,\mathbf{D}\,\mathbf{x}, \tag{165}$$

which proves Eq. (162). □

Corollary 19. *Changing the polar angle of the CZT transform parameter A from α to β is equivalent to changing the elements of the input vector $\mathbf{x}$ by adding $k(\alpha-\beta)$ to the polar angle of each x_k. This change does not affect the norm of $\mathbf{x}$.*

Proof. This corollary follows from Eq. (162). That is, multiplying the input vector $\mathbf{x}$ by the diagonal matrix $\mathbf{D}$, which is generated by a vector of complex exponentials, changes only the polar angles of the elements of $\mathbf{x}$ and leaves their magnitudes unchanged.

More formally, let $x_k = |x_k| e^{i\theta_k}$ be the polar form of x_k for each $k \in \{0, 1, 2, \ldots, N-1\}$. Then,

$$(\mathbf{D}\,\mathbf{x})_k = e^{ki(\alpha-\beta)}\, x_k = |x_k|\, e^{i(\theta_k + k(\alpha-\beta))}. \tag{166}$$

That is, the real term $k(\alpha-\beta)$ is added to the polar angle of x_k, which does not change the Euclidean norm of $\mathbf{x}$. □

Theorem 20. *Let W be a non-zero complex number and let A and B be two non-zero complex numbers such that their magnitudes are equal, i.e., $|A| = |B| = \mu > 0$. Let α be the polar angle of A and let β be the polar angle of B. More formally,*

$$A = |A|\, e^{i\alpha} = \mu\, e^{i\alpha}, \tag{167}$$

$$B = |B|\, e^{i\beta} = \mu\, e^{i\beta}. \tag{168}$$

Let $\mathbf{X} = (X_0, X_1, X_2, \ldots, X_{M-1})$ be a complex vector of length M. Let $\mathbf{x}^A = (x_0^A, x_1^A, x_2^A, \ldots, x_{N-1}^A)$ be the complex vector of length N that is equal to the ICZT of $\mathbf{X}$ parametrized by N, W, and A. That is,

$$\mathbf{x}^A = \mathrm{ICZT}(\mathbf{X}, N, W, A) = \mathbf{A}^{-1}\, \boldsymbol{W}^{-1}\, \mathbf{X}, \tag{169}$$

where $\boldsymbol{W}$ is the Vandermonde matrix defined by Eq. (3) *and $\mathbf{A}$ is the diagonal matrix defined by* Eq. (158).

Similarly, let $\mathbf{x}^B = (x_0^B, x_1^B, x_2^B, \ldots, x_{N-1}^B)$ be the complex vector of length N that is equal to the ICZT of $\mathbf{X}$ parametrized by N, W, and B, i.e.,

$$\mathbf{x}^B = \mathrm{ICZT}(\mathbf{X}, N, W, B) = \mathbf{B}^{-1}\, \boldsymbol{W}^{-1}\, \mathbf{X}, \tag{170}$$

where $\mathbf{B}$ is the diagonal matrix defined by Eq. (160).

Then, $\mathbf{x}^B = \mathbf{D}^{-1}\,\mathbf{x}^A$, where $\mathbf{D}$ is the diagonal matrix defined by Eq. (161).

Proof. Previously, it was proven that $\mathbf{B} = \mathbf{A}\mathbf{D}$. Thus,

$$\mathbf{B}^{-1} = \mathbf{D}^{-1}\mathbf{A}^{-1}. \tag{171}$$

Plugging this expression into Eq. (170) completes the proof, i.e.,

$$\mathbf{x}^B = \mathbf{B}^{-1}\boldsymbol{W}^{-1}\mathbf{X} = \mathbf{D}^{-1}\mathbf{A}^{-1}\boldsymbol{W}^{-1}\mathbf{X} = \mathbf{D}^{-1}\,\mathbf{x}^A. \tag{172}$$

□

Corollary 21. *Changing the polar angle of the ICZT transform parameter A from α to β is equivalent to changing the elements of the output vector $\mathbf{x}$ by subtracting $k(\alpha-\beta)$ from the polar angle of each x_k. This does not affect the norm of $\mathbf{x}$.*

Proof. This corollary follows from Eq. (170), which implies that the polar angle of x_k^B can be obtained from the polar angle of x_k^A by subtracting $k(\alpha-\beta)$. In other words,

$$x_k^B = e^{-ki(\alpha-\beta)}\, x_k^A = |x_k^A|\, e^{i(\theta_k^A - k(\alpha-\beta))}, \tag{173}$$

where θ_k^A is the polar angle of x_k^A. Therefore, $\|\mathbf{x}^B\| = \|\mathbf{x}^A\|$. □

Supplementary Appendix I: Approximation Formulas for the Numerical Error

This appendix states formulas that approximate the absolute numerical error of the CZT algorithm, the ICZT algorithm, and their sequential applications. The formulas are given for the square case in which $M = N$. The formulas apply to the modified algorithms described in Appendix G that reverse the direction of the chirp contour when $|W| < 1$. Finally, these formulas are suitable only for the case when the chirp contour is a logarithmic spiral that spans a 360° arc.

The error formulas are stated using the following four terms:

$$t_{1,k} = W^{\frac{k^2}{2}} A^{-k}, \tag{174}$$

$$t_{2,k} = W^{-\frac{k^2}{2}}, \tag{175}$$

$$t_{3,k} = W^{\frac{k^2}{2}}, \tag{176}$$

$$t_{4,k} = W^{-\frac{k^2}{2}} A^{k}, \tag{177}$$

which are defined for each $k \in \{0, 1, 2, \ldots, N-1\}$. The first term corresponds to the factors used on line 7 of Algorithm 1. The second term is used on lines 8 and 11 of Algorithm 1 and on line 9 of Algorithm 2. The third term is used on line 16 of Algorithm 1. The fourth term is used on line 33 of Algorithm 2.

All of these lines are inside loops that perform N iterations (in the square case). Thus, each term specifies the k-th element of a vector. The error formulas use four aggregate terms that are computed by taking the logarithm of the Euclidean norm of these four vectors, i.e.,

$$T_1 = \log \sqrt{\sum_{k=0}^{N-1} |t_{1,k}|^2} = \frac{1}{2} \log \sum_{k=0}^{N-1} |W|^{k^2} |A|^{-2k}, \tag{178}$$

$$T_2 = \log \sqrt{\sum_{k=0}^{N-1} |t_{2,k}|^2} = \frac{1}{2} \log \sum_{k=0}^{N-1} |W|^{-k^2}, \tag{179}$$

$$T_3 = \log \sqrt{\sum_{k=0}^{N-1} |t_{3,k}|^2} = \frac{1}{2} \log \sum_{k=0}^{N-1} |W|^{k^2}, \tag{180}$$

$$T_4 = \log \sqrt{\sum_{k=0}^{N-1} |t_{4,k}|^2} = \frac{1}{2} \log \sum_{k=0}^{N-1} |W|^{-k^2} |A|^{2k}. \tag{181}$$

The error formulas that cover the ICZT algorithm also use the following three terms that are computed from the elements of the vector $\mathbf{u}$, which is defined by Eq. (18):

$$U_1 = \log \sqrt{\sum_{k=1}^{N-1} |\mathrm{u}_k|^2} = \frac{1}{2} \log \sum_{k=1}^{N-1} |\mathrm{u}_k|^2, \tag{182}$$

$$U_2 = \log \sqrt{\sum_{k=0}^{N-1} |\mathrm{u}_k|^2} = \frac{1}{2} \log \sum_{k=0}^{N-1} |\mathrm{u}_k|^2, \tag{183}$$

$$U_3 = -\log |\mathrm{u}_0|. \tag{184}$$

The term U_1 is equal to the logarithm of the Euclidean norm of a vector formed by all elements of the vector $\mathbf{u}$ except u_0. It maps to lines 25 and 26 of Algorithm 2. The term U_2 is similar, but it uses all elements of the vector $\mathbf{u}$, including u_0. It maps to lines 27 and 28 of Algorithm 2. Finally, U_3 is equal to the negative logarithm of the magnitude of u_0 (by definition, u_0 cannot be zero). It maps to line 30 of Algorithm 2.

All error formulas also include an offset that depends on N and p, where p is the number of precision bits in the IEEE-754 floating-point numbers[33] that are used to compute the transforms. For computations with 128, 256, 512, and 1024 bits, the value of p is equal to 113, 237, 489, and 997, respectively. This offset determines the base level of the error surface. It is defined as follows:

$$B = -\log(2^p N) = -p \log 2 - \log N. \tag{185}$$

For this evaluation, the terms in Eqs. (174)–(177) and the elements of the vector $\mathbf{u}$ were computed with very high precision (1024 bits). This was done to reduce the effect of the numerical error in these terms on the overall numerical error. All other computations were performed using 128 bits.

A. Error Formula for the CZT Algorithm

The CZT maps the input vector $\mathbf{x}$ to the output vector $\hat{\mathbf{X}}$. The absolute numerical error is given by:

$$E = \|\hat{\mathbf{X}} - \mathbf{X}\|, \tag{186}$$

where $\mathbf{X}$ is the true but unknown output vector of the transform. Figure S4a plots the decimal logarithm of E as a function of A and W. This surface can be approximated with the following error formula:

$$\log E \approx T_1 + T_2 + T_3 + B + \log \|\mathbf{x}\|. \tag{187}$$

This formula fits the empirical data very well, as indicated by the R^2 values in the second column of Table S1. Furthermore, the quality of the fit improves as N increases.

B. Error Formula for the ICZT Algorithm

The ICZT algorithm maps $\mathbf{X}$ to $\hat{\mathbf{x}}$. Its absolute numerical error is given by:

$$E = \|\hat{\mathbf{x}} - \mathbf{x}\|, \tag{188}$$

where $\hat{\mathbf{x}}$ is the computed output vector and $\mathbf{x}$ is the true but unknown output vector. Figure S4b plots the decimal logarithm of E as a function of A and W. The error can be predicted using the following formula:

$$\log E \approx T_2 + T_4 + U_1 + U_2 + U_3 + B + \log \|\mathbf{X}\|. \tag{189}$$

The goodness of fit is reported in Table S1 for different N.

N	CZT	ICZT	CZT–ICZT	ICZT–CZT
64	0.99963	0.99976	0.99846	0.99970
128	0.99994	0.99988	0.99900	0.99987
256	0.99998	0.99995	0.99966	0.99997
512	0.99999	0.99996	0.99992	0.99998

Table S1. The R^2 values for fitting error models to empirically derived error surfaces. Each point on each surface was computed by averaging the numerical errors over 10 random unit-length input vectors. The discretizations for A and W are the same as in the main text. Perfect fits correspond to $R^2 = 1$. In all four cases, the R^2 values are very close to 1 and improve as N increases.

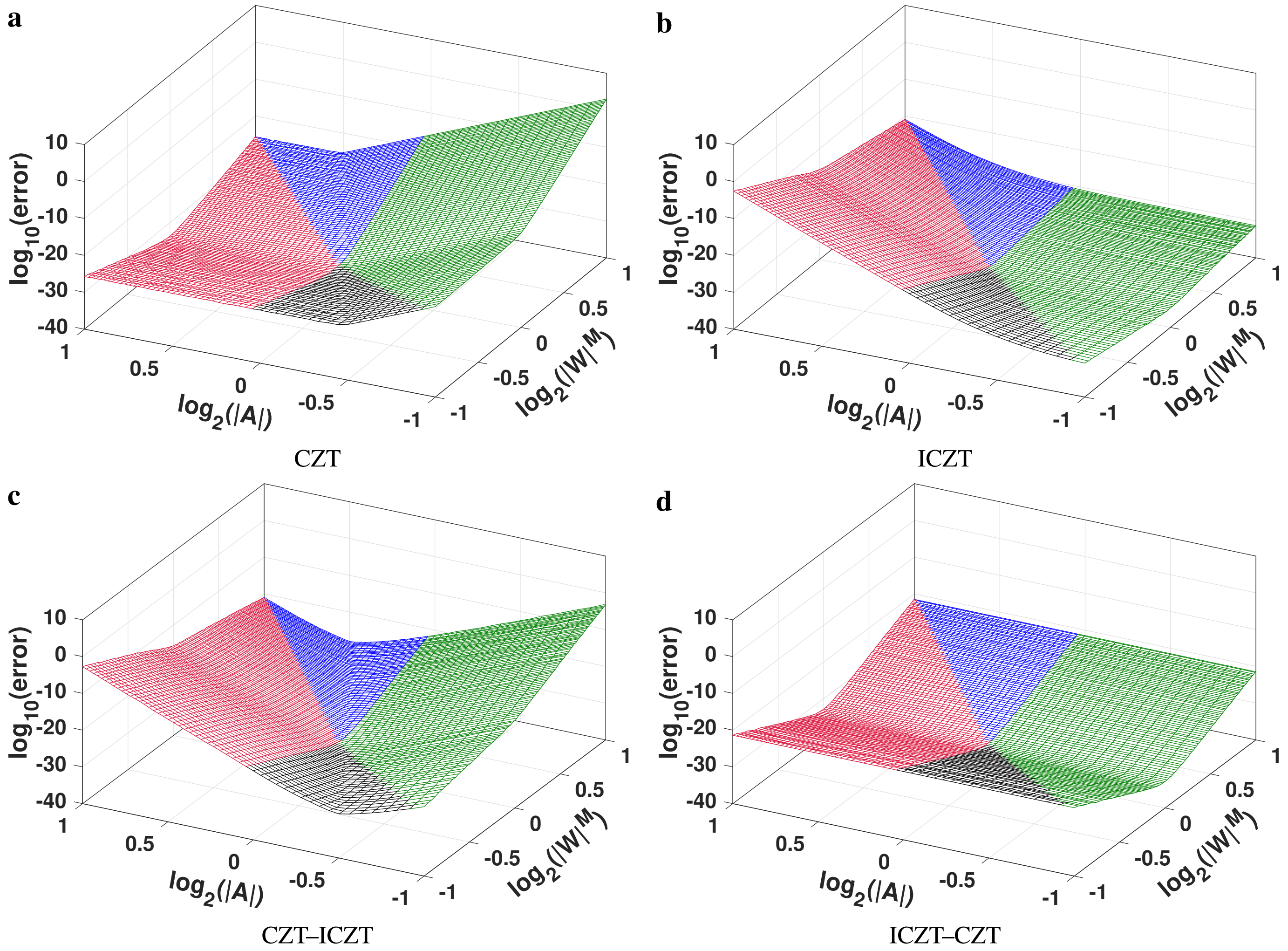

Figure S4. The absolute numerical error for $N = M = 64$ as a function of A and W, shown for: (**a**) the CZT transform; (**b**) the ICZT transform; (**c**) the CZT followed by the ICZT; and (**d**) the ICZT followed by the CZT. These surfaces were computed using the modified algorithms that reverse the direction of the chirp contour when $|W| < 1$, i.e., using Algorithm S8 and Algorithm S9. The points in all surfaces represent the average error for 10 randomly-generated unit-length input vectors. The ground truth output vectors were approximated using 1024-bit floating-point numbers. All other computations used 128-bit numbers. Each surface consists of 5,200 points generated using the discretization described in the Methods section. That is, 52 evenly-distributed points in the range $[0.5, 2.0]$ for $|A|$ and 100 evenly-distributed points in the same range for $|W|^M$. All three axes in each of the four plots are scaled logarithmically.

C. Error Formula for the CZT Followed by the ICZT

For the CZT–ICZT procedure, the absolute numerical error is equal to the Euclidean distance between the original input vector $\mathbf{x}$ and the computed vector $\hat{\mathbf{x}}$. More formally,

$$E = \|\hat{\mathbf{x}} - \mathbf{x}\|. \tag{190}$$

Figure S4c plots E as a function of the transform parameters A and W. The error values can be analytically approximated using the following formula:

$$\log E \approx T_1 + T_2 + T_4 + U_1 + U_2 + U_3 + B + \log\|\mathbf{x}\|. \tag{191}$$

The goodness of fit of this expression is evaluated in Table S1.

This formula can be viewed as a sum of the terms in Eqs. (187) and (189). Because the output scaling on line 16 of Algorithm 1 is immediately canceled by the input scaling on line 9 of Algorithm 2, the corresponding terms T_3 and T_2 are excluded from Eq. (191). The term $\log\|\mathbf{X}\|$ is excluded as well. Also, the term B is counted only once.

D. Error Formula for the ICZT Followed by the CZT

The absolute error of the ICZT–CZT procedure is given by:

$$E = \|\hat{\mathbf{X}} - \mathbf{X}\|, \tag{192}$$

where $\mathbf{X}$ is the true output vector and $\hat{\mathbf{X}}$ is the computed output vector. Figure S4d visualizes the decimal logarithm of the error, which can be approximated as follows:

$$\log E \approx 2T_2 + T_3 + U_1 + U_2 + U_3 + B + \log\|\mathbf{X}\|. \tag{193}$$

This formula can be viewed as a sum of the terms in Eqs. (187) and (189) except for B, which is included only once, and $\log\|\mathbf{x}\|$, which is excluded. Also, terms T_1 and T_4 are excluded because the output scaling performed on line 33 of the ICZT algorithm is immediately undone by the input scaling on line 7 of the CZT algorithm. This implies that the absolute numerical error is independent of A, which is confirmed by Fig. S4d. Furthermore, the last column of Table S1 shows that Eq. (193) approximates the empirical error very well.
